# Collateral Sensitivity to β-Lactam Drugs in Drug-Resistant Tuberculosis Is Driven by the Transcriptional Wiring of BlaI Operon Genes

**DOI:** 10.1128/mSphere.00245-21

**Published:** 2021-05-28

**Authors:** A. S. Trigos, B. W. Goudey, J. Bedő, T. C. Conway, N. G. Faux, K. L. Wyres

**Affiliations:** a Peter MacCallum Cancer Centre, Melbourne, Victoria, Australia; b Sir Peter MacCallum Department of Oncology, The University of Melbourne, Parkville, Victoria, Australia; c Melbourne Data Analytics Platform, The University of Melbourne, North Melbourne, Victoria, Australia; d The School of Computing and Information Systems, The University of Melbourne, Parkville, Victoria, Australia; e Centre For Epidemiology and Biostatistics, The University of Melbourne, Parkville, Victoria, Australia; f The Florey Institute of Neuroscience and Mental Health, The University of Melbourne, Parkville, Victoria, Australia; g Department of Infectious Diseases, Central Clinical School, Monash University, Melbourne, Victoria, Australia; h Bioinformatics Division, The Walter and Eliza Hall Institute of Medical Research, Parkville, Victoria, Australia; University of Nebraska Medical Center

**Keywords:** β-lactams, antimicrobial resistance, bioinformatics, collateral sensitivity, *in silico*, network analysis, paradoxical hypersensitivity, tuberculosis

## Abstract

The evolution of resistance to one antimicrobial can result in enhanced sensitivity to another, known as “collateral sensitivity.” This underexplored phenomenon opens new therapeutic possibilities for patients infected with pathogens unresponsive to classical treatments. Intrinsic resistance to β-lactams in Mycobacterium tuberculosis (the causative agent of tuberculosis) has traditionally curtailed the use of these low-cost and easy-to-administer drugs for tuberculosis treatment. Recently, β-lactam sensitivity has been reported in strains resistant to classical tuberculosis therapy, resurging the interest in β-lactams for tuberculosis. However, a lack of understanding of the molecular underpinnings of this sensitivity has delayed exploration in the clinic. We performed gene expression and network analyses and *in silico* knockout simulations of genes associated with β-lactam sensitivity and genes associated with resistance to classical tuberculosis drugs to investigate regulatory interactions and identify key gene mediators. We found activation of the key inhibitor of β-lactam resistance, *blaI*, following classical drug treatment as well as transcriptional links between genes associated with β-lactam sensitivity and those associated with resistance to classical treatment, suggesting that regulatory links might explain collateral sensitivity to β-lactams. Our results support M. tuberculosis β-lactam sensitivity as a collateral consequence of the evolution of resistance to classical tuberculosis drugs, mediated through changes to transcriptional regulation. These findings support continued exploration of β-lactams for the treatment of patients infected with tuberculosis strains resistant to classical therapies.

**IMPORTANCE** Tuberculosis remains a significant cause of global mortality, with strains resistant to classical drug treatment considered a major health concern by the World Health Organization. Challenging treatment regimens and difficulty accessing drugs in low-income communities have led to a high prevalence of strains resistant to multiple drugs, making the development of alternative therapies a priority. Although Mycobacterium tuberculosis is naturally resistant to β-lactam drugs, previous studies have shown sensitivity in strains resistant to classical drug treatment, but we currently lack understanding of the molecular underpinnings behind this phenomenon. We found that genes involved in β-lactam susceptibility are activated after classical drug treatment resulting from tight regulatory links with genes involved in drug resistance. Our study supports the hypothesis that β-lactam susceptibility observed in drug-resistant strains results from the underlying regulatory network of M. tuberculosis, supporting further exploration of the use of β-lactams for tuberculosis treatment.

## INTRODUCTION

Collateral antimicrobial sensitivity occurs when the evolution of resistance to one or more antimicrobials directly or indirectly causes increased sensitivity to unrelated antimicrobials (1). There are now numerous examples of this phenomenon in the literature (2, 3), and while direct mechanisms are sometimes evident based on our understanding of individual genes or pathways (4), there is a lack of knowledge to explain collateral sensitivity between drugs of unrelated function. An improved understanding of such mechanisms can inform novel treatment strategies that limit or delay the development of resistance (1).

Tuberculosis (TB) remains a significant cause of global mortality, causing an estimated 1.4 million deaths annually (5). It can be successfully treated through combination antimicrobial therapy targeting the causal pathogen, Mycobacterium tuberculosis. However, successful treatment is hampered by the continuing rise of antimicrobial-resistant M. tuberculosis, particularly strains resistant to multiple drugs (5). According to the World Health Organization, multidrug resistance among M. tuberculosis strains is defined as resistance to isoniazid and rifampicin, with or without resistance to other first-line drugs, and extensive drug resistance is defined as resistance to isoniazid and rifampicin plus any fluoroquinolone and any of the three second-line injectable drugs (amikacin, capreomycin, and kanamycin).

The potential application of clinical regimens including β-lactams for the treatment of TB is of particular interest due to the comparative low-cost, ease of treatment, and accessibility of these drugs (6). However, M. tuberculosis has generally been considered intrinsically resistant to β-lactams due to (i) the inclusion of nonclassical peptidoglycan linkages in its cell wall, by a combination of distinct penicillin binding proteins (7, 8), and (ii) the presence of BlaC β-lactamases that break down β-lactams with drug-specific efficiencies (9–11). However, sensitivity to certain subclasses of β-lactams in M. tuberculosis, namely, faropenem (12) and carbapenems (7, 13), was recently reported, although coadministration with a β-lactamase inhibitor, such as clavulanate in a combination known as Augmentin, is likely needed to be effective *in vivo* (6, 10, 14). Unfortunately, patient treatment trials of this combination have been far from promising (15, 16), and recent reports of novel β-lactamases (17) have added another layer of complexity to the use of β-lactams in the clinic.

Multidrug- or extensively drug-resistant clinical M. tuberculosis strains and isolates experimentally evolved to be resistant to aminoglycosides have been shown to exhibit enhanced sensitivity to the β-lactams with and without the addition of β-lactamase inhibitors (18–20), suggesting that β-lactam sensitivity may be associated with the evolution of resistance to classical TB drugs as a result of a process of collateral sensitivity. Therefore, exploration of the potential mechanism(s) linking β-lactam sensitivity with resistance to the classical drugs may help elucidate the biological underpinnings of β-lactam sensitivity.

Technological and algorithmic advances have facilitated the high-throughput measurement of gene expression as well as the inference and analysis of large-scale protein-protein interaction and DNA-protein interaction networks for M. tuberculosis, which can facilitate systems-level investigations into the transcriptional and regulatory mechanisms. These methods have been successfully adopted in the study of M. tuberculosis infection (21), latency (22), and response to the β-lactam plus β-lactamase inhibitor treatment (23) and for the identification of novel drug resistance mechanisms (24, 25). However, a systems-level understanding of collateral sensitivity in drug-resistant M. tuberculosis is currently lacking.

Here, we leverage network and transcriptomic analyses for the exploration of collateral β-lactam sensitivity in M. tuberculosis. We combine gene expression analyses with protein-protein interaction, gene regulatory network data, and functional *in silico* growth simulations. Our analyses suggest that collateral β-lactam sensitivity is the result of direct transcriptional regulation between genes associated with β-lactam sensitivity and those mediating resistance to classical TB drugs. This wiring promotes the inhibition of β-lactamases as a response to drug treatment, with genes of the BlaI operon that inhibit the blaC β-lactamases (*blaI*, *sigC*, and *atpH*), playing key roles.

## RESULTS

### Treatment with classical TB drugs induces the expression of β-lactamase inhibitors.

If β-lactam sensitivity in M. tuberculosis is truly a consequence of classical drug resistance (i.e., truly collateral), we expect that genes/proteins implicated in β-lactam sensitivity (β-lactam^s^ genes) (see Table S1 in the supplemental material) should have close biochemical and/or regulatory connections to those that are implicated in classical drug resistance (DR genes) (see Table S2). We hypothesized that such connectivity may be detected as differential expression of β-lactam^s^ genes in response to classical drug treatment. Therefore, we investigated the differential expression of 199 genes with reported involvement in β-lactam sensitivity in M. tuberculosis (or the closely related species Mycobacterium smegmatis or Mycobacterium bovis) in response to incubation with classical TB drugs (ethambutol [EMB], ethionamide [ETH], two fluoroquinolones [FLQs]; levofloxacin and ofloxacin), (aminoglycosides [AMI], streptomycin [SM], isoniazid [INH], pyrazinamide [PZA], and rifampicin [RIF]) (26). Since we aimed to find commonalities between drug treatments, gene expression data across single-agent drug treatments were pooled. Given that many of the reported β-lactam^s^ genes have not yet been subjected to experimental validation, we primarily focused on a subset of 63 high-confidence genes (Table S1), which included canonical genes such as *blaI*, *bla*, and *atpA-G*, which were shown to have β-lactamase activity, are downstream of *blaI*, or have been identified through functional assays as being associated with sensitivity to β-lactams. We excluded genes solely identified through synthetic lethality or transposon mutant screens or with circumstantial evidence of involvement (see Materials and Methods). However, all analyses were repeated using the full set of 199 genes, reported in Table S6.

10.1128/mSphere.00245-21.5 TABLE S1 Genes associated with β-lactam susceptibility. Download Table S1, DOCX file, 0.1 MB. Copyright © 2021 Trigos et al. 2021 Trigos et al. https://creativecommons.org/licenses/by/4.0/ This content is distributed under the terms of the Creative Commons Attribution 4.0 International license.

10.1128/mSphere.00245-21.6 TABLE S2 Genes involved in classical resistance. Download Table S2, DOCX file, 0.1 MB. Copyright © 2021 Trigos et al. 2021 Trigos et al. https://creativecommons.org/licenses/by/4.0/ This content is distributed under the terms of the Creative Commons Attribution 4.0 International license.

10.1128/mSphere.00245-21.10 TABLE S6 Comparison of analysis results using high-confidence versus all β-lactam^S^-associated genes. Download Table S6, DOCX file, 0.1 MB. Copyright © 2021 Trigos et al. 2021 Trigos et al. https://creativecommons.org/licenses/by/4.0/ This content is distributed under the terms of the Creative Commons Attribution 4.0 International license.

**FIG 1:**
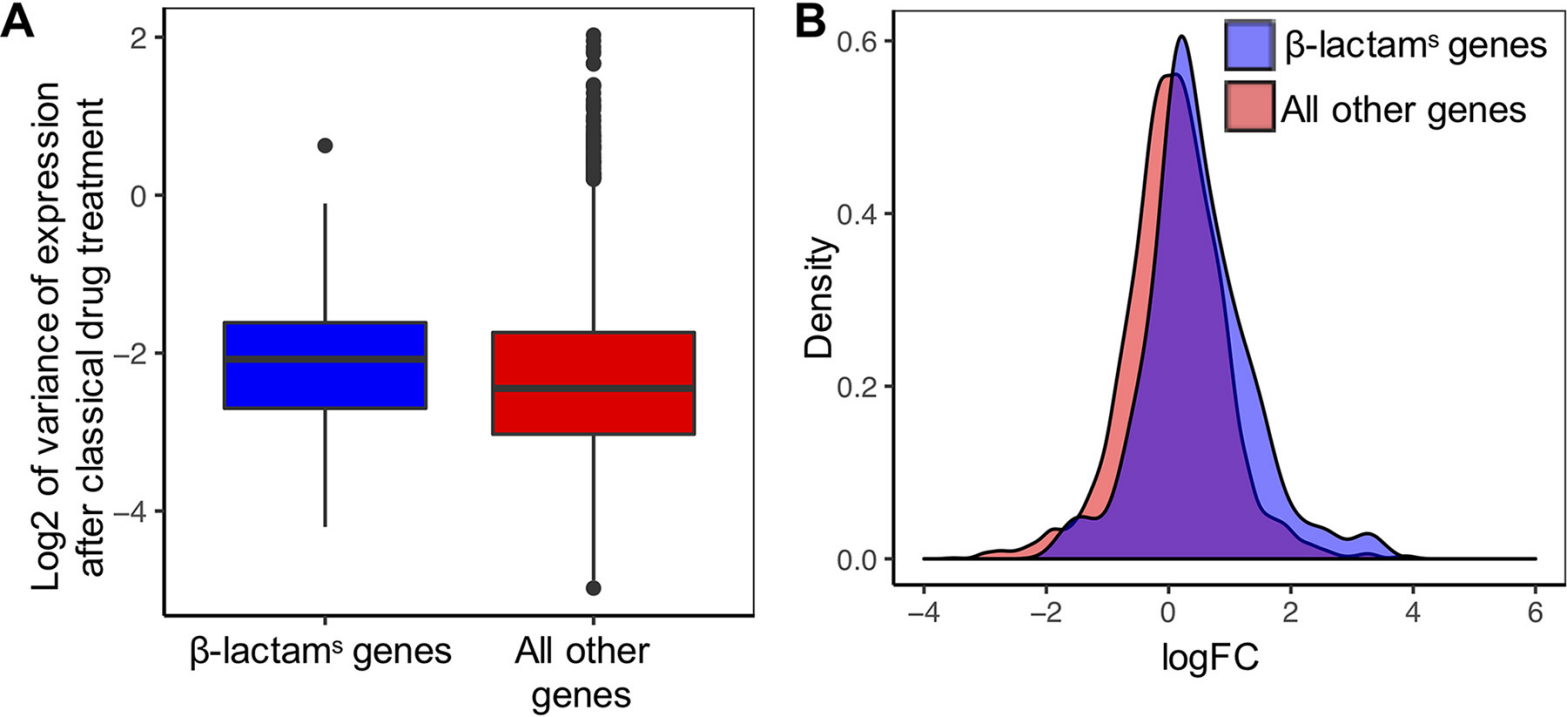
The expression of β-lactam sensitivity (β-lactam^s^) genes is affected by treatment with classical TB drugs. (A) β-lactam^s^ genes tend to be more variable than non-β-lactam^s^ genes, suggesting these genes are affected by drug treatment (KS test *P* value, 0.0027). (B) Log fold change (logFC) of β-lactam^s^ genes and all other non-β-lactam^s^ genes after classical drug treatment. β-lactam^s^ genes tend to have a more positive logFC than other genes, suggesting preferential activation.

We found that β-lactam^s^ genes showed a greater variability of expression than non-β-lactam^s^ genes (Kolmogorov-Smirnov [KS] test *P* value = 0.027; Wilcoxon test *P* value = 0.014) (Fig. 1A), indicating that classical drug treatment disproportionately affects the activity of these genes. We further validated this result by assessing the variability of randomly selected subsets of non-β-lactam^s^ genes matching the number of β-lactam^s^ genes (10,000 permutations, *P* = 0.023).

We next performed differential expression analysis using limma (27), revealing 483/3,947 differentially expressed genes after drug treatment (*q* value < 0.05, |fold change [FC]| > 2) (Fig. 1B; see also Table S3), with 268 of these being upregulated (6.79%). Inspection of the β-lactam^s^ genes revealed that 18.52% (10 of 54 in the data set) of β-lactam^s^ genes were significantly upregulated. This indicated an enrichment of β-lactam^s^ genes among the differentially expressed genes (Fisher exact test *P* = 0.0075).

10.1128/mSphere.00245-21.7 TABLE S3 Changes in gene expression after drug treatment. Differential expression was calculated comparing expression levels after classical tuberculosis drug treatment (pooled across drugs) and in control growth medium. Download Table S3, DOCX file, 0.1 MB. Copyright © 2021 Trigos et al. 2021 Trigos et al. https://creativecommons.org/licenses/by/4.0/ This content is distributed under the terms of the Creative Commons Attribution 4.0 International license.

To assess whether the activation of β-lactam^s^ genes may be a reflection of a stress response, we also investigated the expression levels of β-lactam^s^ genes in acidic environments (pH 5.5 and 6.5) (using data from reference 28, GEO accession GSE8827) and during a time course hypoxia experiment from 4 h to 8 days (using data from reference 29, GEO accession GSE9331). β-Lactam^s^ genes did not show patterns of increased expression under acidic conditions compared to that in the control (see Fig. S1). Only two β-lactam^s^ genes (Rv3290c and Rv0849) showed increased expression as an acute response to hypoxia within the first 8 h of treatment, while the rest either maintained similar levels or decreased their expression (see Fig. S2). This supports the notion that drug treatment is a more specific inducer of the expression of β-lactam^s^ genes than acidic stress conditions.

10.1128/mSphere.00245-21.1 FIG S1 Expression levels of β-lactam^s^ genes under acidic conditions. The vast majority of high-confidence (A and B) and all β-lactam^s^ genes (C and D) showed similar levels of expression under control and acidic (pH 6.5 and 5.5) conditions. Each data point corresponds to a gene. Data obtained from reference 28. Download FIG S1, DOCX file, 0.2 MB. Copyright © 2021 Trigos et al. 2021 Trigos et al. https://creativecommons.org/licenses/by/4.0/ This content is distributed under the terms of the Creative Commons Attribution 4.0 International license.

10.1128/mSphere.00245-21.2 FIG S2 Expression levels of β-lactam^s^ genes under hypoxic conditions. The vast majority of high-confidence (A) and all β-lactam^s^ genes (B) did not significantly alter their expression levels under hypoxic conditions. Only 1 high-confidence gene (Rv0849) and 2 from the complete β-lactam^s^ gene list (Rv0849 and Rv3290c) had altered expression within the first 24 h. Data obtained from reference 29. Download FIG S2, DOCX file, 0.2 MB. Copyright © 2021 Trigos et al. 2021 Trigos et al. https://creativecommons.org/licenses/by/4.0/ This content is distributed under the terms of the Creative Commons Attribution 4.0 International license.

Interestingly, *blaI* (Rv1846c), the major repressor of the *blaC* β-lactamase, and *atpH* (Rv1307) and *sigC* (Rv2069), members of the BlaI regulon (30), were all upregulated after classical drug treatment (pooled results: *blaI* fold change = 2.85, *q* value = 0.0095; *atpH* fold change = 2.25, *q* value = 0.030; *sigC* fold change = 2.70, *q* value = 0.0004) (Table S3; Fig. S3). Given that an increased activity of the *blaI* repressor would lead to a loss of activity of *blaC* and therefore a reduction in β-lactamase production (30), these data indicate that classical TB drug treatment may inhibit the main β-lactamase responsible for M. tuberculosis’s intrinsic β-lactam resistance. Among the upregulated genes we also found Rv1884c (*rpfC*) (fold change = 3.33, *q* value = 0.0324), which has also been associated with β-lactam sensitivity (31). Overall, our data suggest that treatment of M. tuberculosis with classical anti-TB drugs used in the clinic promoted the upregulation of key inhibitors of β-lactam resistance.

10.1128/mSphere.00245-21.3 FIG S3 Levels of expression of *blaI*, *sigC*, *atpH*, *blaR*, and *blaC* after drug treatment. The distribution of normalized expression values showed increased *blaI* expression after classical drug treatment. *sigC* and *atpH* also showed upregulation after drug treatment. There was no consistent signal across drug treatments for *blaR*, resulting in a nonsignificant difference (*q* value = 0.82, FC = 1.08). In the case of *blaC*, there was upregulation, but this was less marked than for *blaI*, *sigC*, and *atpH* and it was not significant (*q* value = 0.06 and FC = 1.43). Download FIG S3, DOCX file, 0.3 MB. Copyright © 2021 Trigos et al. 2021 Trigos et al. https://creativecommons.org/licenses/by/4.0/ This content is distributed under the terms of the Creative Commons Attribution 4.0 International license.

### Strong coexpression between β-lactam^s^ and DR genes.

The findings from our gene expression analyses were consistent with our hypothesis that there is a transcriptional association between β-lactam^s^ genes and those encoding the classical drug targets. To further explore this hypothesis, we searched for coexpression associations between the β-lactam^s^ and DR genes, the latter of which include those that encode the classical drug target proteins.

First, we investigated module comembership of β-lactam^s^ genes with DR genes among previously defined coexpression modules derived from 437 perturbation experiments with different drugs and growth-inhibitory conditions (26). We found that 50% of coexpression clusters with at least 2 β-lactam^s^ genes also contained DR genes, with permutation analysis indicating that this was unlikely to occur by chance (*P* = 0.12, based on 10,000 randomizations of cluster membership), suggesting that these genes are controlled by similar regulatory networks.

**FIG 2:**
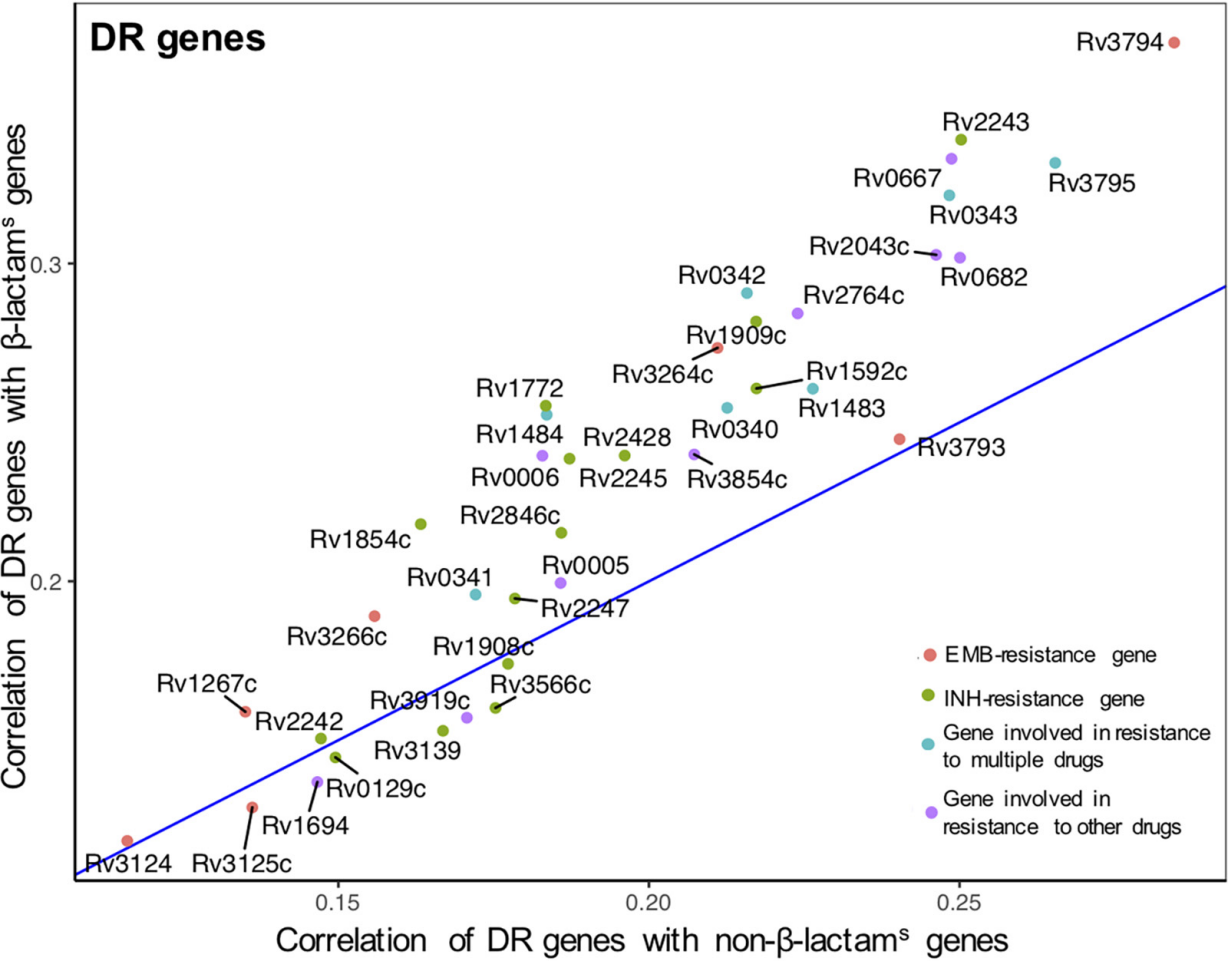
Upperquantile of expression correlation of drug resistance (DR) genes with β-lactam sensitivity (β-lactam^s^) genes (*y* axis) and non-β-lactam^s^ genes (*x* axis). Genes above the diagonal line are more strongly coexpressed with β-lactam^s^ genes than with other genes, and vice versa. The genes with the strongest positive correlation of expression (well above the diagonal line) are implicated in isoniazid (INH) and ethambutol (EMB) resistance.

Next, we compared the strength of correlation of expression of DR genes with β-lactam^s^ genes in these perturbation experiments (see Materials and Methods). We found that of the 37 DR genes, 30 (81.1%) had a stronger correlation with the high-confidence genes of the β-lactam^s^ cluster than with all other genes (Fig. 2, genes located above the diagonal line). These DR genes were disproportionately associated with INH resistance (13 genes) or with resistance to multiple drugs (7 genes), suggesting that these DR genes likely exert a strong influence on genes associated with β-lactam sensitivity.

Overall, DR and β-lactam^s^ genes were found to be highly coexpressed in the transcriptional network of M. tuberculosis, supporting our hypothesis of a transcriptional association of these genes.

### β-lactam^s^ and DR nodes (genes/proteins) are highly linked in the molecular network of M. tuberculosis.

To determine whether the transcriptional associations between β-lactam^s^ and DR genes were the result of direct regulation between these genes as opposed to indirect associations, we investigated their localization and interaction in the M. tuberculosis protein-protein and gene regulatory networks, where genes/proteins are represented as nodes and interactions are represented as edges. We integrated the STRING database (32) (here referred to as the protein-protein interaction [PPI] network) and transcription factor-target gene data published in reference 33 (here referred as to the gene regulatory [GR] network), excluding duplicated edges and self-loops. The resulting network contained 4,181 nodes (genes/proteins) and 37,313 edges, including experimentally validated physical and transcription factor-target associations between genes or proteins. There was no evidence to suggest that the combined PPI and GR network was not drawn from a power-law distribution (*P* = 0.065, i.e., indicating that we cannot reject the null hypothesis that the degree of distribution follows a power-law distribution), supporting the view that the network structure is consistent with a true biological network (34).

**FIG 3:**
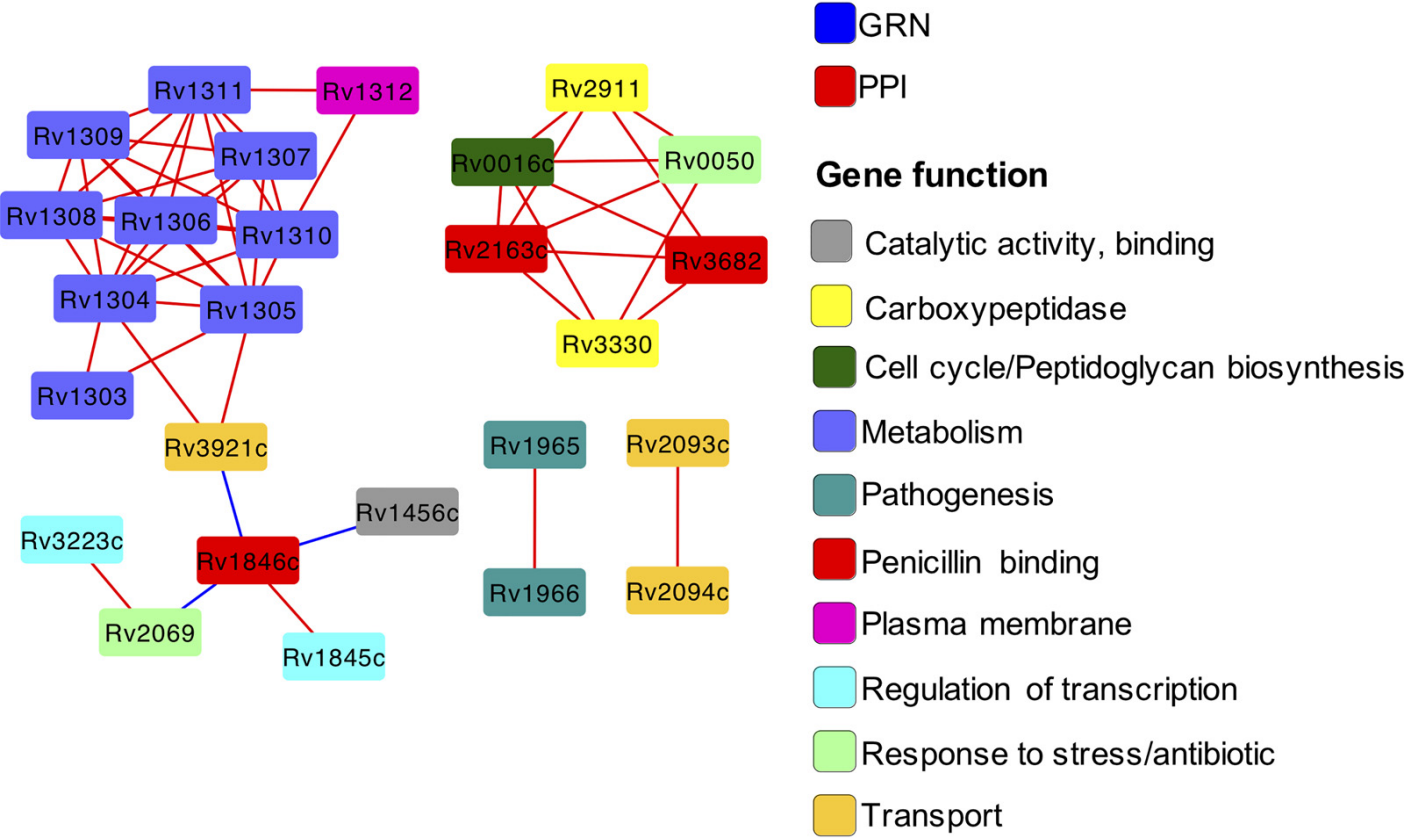
Network of interactions between genes/proteins associated with β-lactam sensitivity. β-Lactam^S^ (β-lactam sensitivity) genes form a single interconnected network, with a few exceptions, indicating a high degree of localization in the global M. tuberculosis network. Nodes are colored by predicted functional categories. The network shown is a combination of the protein-protein interaction (PPI; red edges) and the gene regulatory (GR; blue edges) networks.

We found that 26 of the 63 (41%) β-lactam^s^ nodes were localized in a highly specific network region, interacting with each other (Fig. 3). We assessed the significance of the interactions between gene sets modeling the distribution of cross talk expected under a random model of a given network as a hypergeometric distribution (35) (see Materials and Methods). We found that β-lactam^s^ nodes were more likely to interact with each other than expected by chance in the largest interconnected component of the network region (*q* value = 1 × 10^−35^). Interestingly, within this subnetwork, β-lactam^s^ nodes were clustered based on the gene/protein functional role (Fig. 3), with clusters of nodes representing genes/proteins involved in similar functions, such as metabolism, consistent with previous findings that gene function is related to network localization (36, 37). However, the broader clustering of β-lactam^s^ nodes suggests a high degree of association between these genes even when these are functionally highly varied (Table S1), suggesting involvement in similar protein complexes or enzymatic reactions.

**FIG 4:**
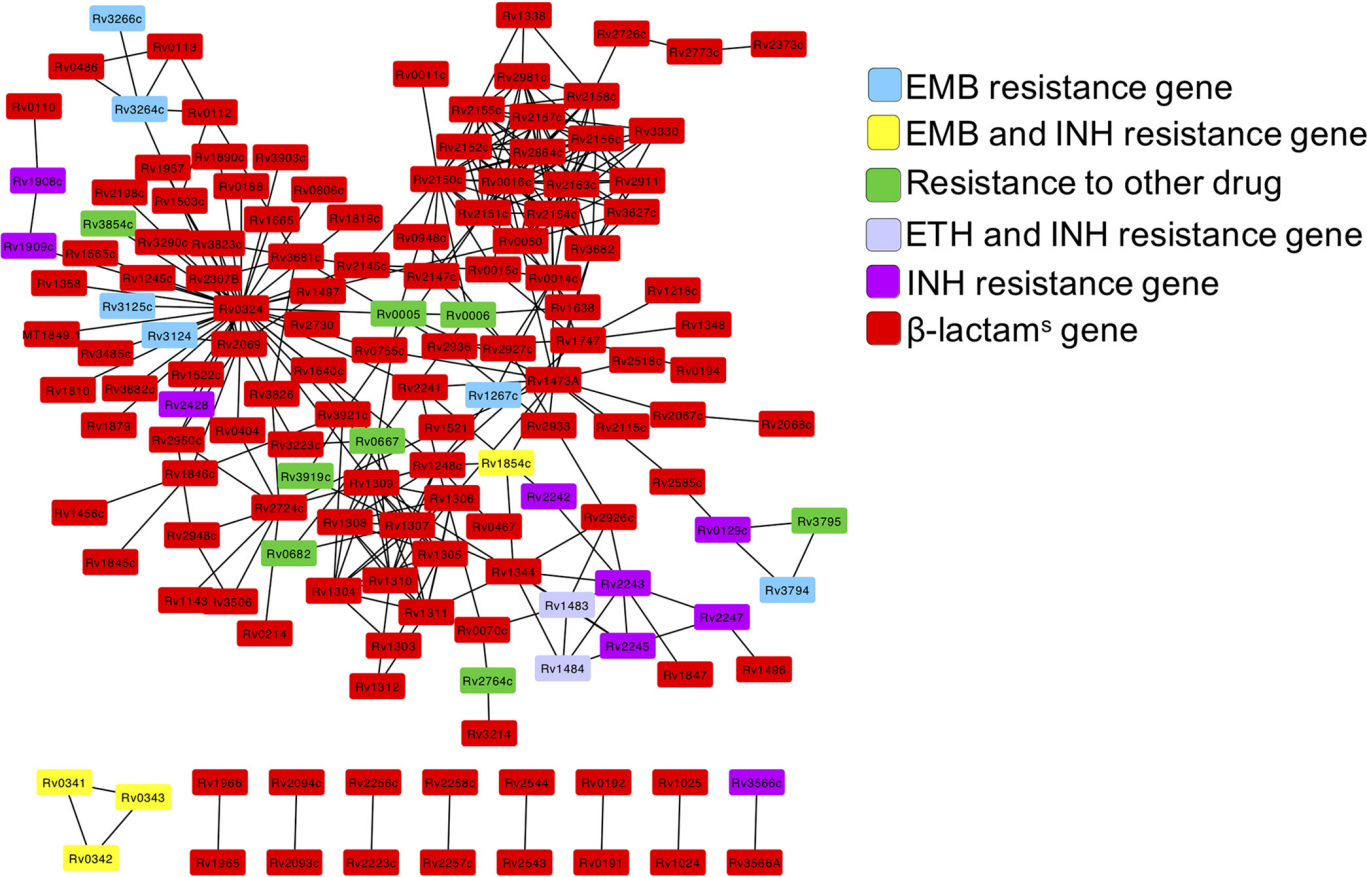
Network of interactions between β-lactam sensitivity (β-lactam^S^) and drug resistance (DR) genes/proteins. β-Lactam^S^ genes/proteins tend to be located toward the core of the network, connecting distinct subgroups of DR genes. The network shown is a combination of the protein-protein interaction and the gene regulatory networks. EMB, ethambutol; INH, isoniazid; ETH, ethionamide.

Next, we investigated the interactions between β-lactam^s^ nodes and DR nodes (Fig. 4; see also Table S4). We noted that the β-lactam^s^ nodes were located near the core or center of the subnetwork, with DR nodes organized in clusters at the periphery grouped by drug type. The degree of cross talk in the most highly connected component of the network was significant (*q* value = 0.0002). We found significant cross talk in the GR network between RIF and SM resistance and β-lactam^s^ genes (*q* value = 0.0003 and 0.003, respectively). These data support direct links between β-lactam^s^ and DR genes, which together with their strong transcriptional associations, support our hypothesis of direct regulatory interactions between β-lactam^s^ genes/proteins and the genes/proteins implicated in resistance to at least three of the first-line treatments used to treat TB in the clinic.

10.1128/mSphere.00245-21.8 TABLE S4 Network of interactions between β-lactam^s^ genes and DR genes. Scores represent quality/confidence in the interaction, as reported by the corresponding databases, and explained in the main text. RIF, rifampicin; INH, isoniazid; AMI, aminoglycoside; SM, streptomycin; FLQ, fluoroquinolone; EMB, ethambutol; ETH, ethionamide; PAS, *para*-aminosalicylic acid; PZA, pyrazinamide. Download Table S4, DOCX file, 0.1 MB. Copyright © 2021 Trigos et al. 2021 Trigos et al. https://creativecommons.org/licenses/by/4.0/ This content is distributed under the terms of the Creative Commons Attribution 4.0 International license.

### Mediators of the interactions between DR and β-lactam^s^ genes.

To identify the key genes linking β-lactam^s^ and DR genes/proteins, which likely mediate collateral β-lactam sensitivity, we performed random walks between the β-lactam^s^ and DR nodes in the PPI and GR networks to determine the influence of one node over another (access times). Random walks correspond to the possible paths taken by a random walker on a network between a pair of nodes, and access times represent the ease with which information (e.g., signal transduction and gene regulation) flows from one node to another, as it is proportional to the number of connections and available paths between nodes.

Since the importance of a node highly depends on the underlying network structure, we first determined how the structural differences between the PPI and GR networks would affect the random walks by comparing access times between pairs of DR nodes and the full set of β-lactam^s^ nodes. We considered both directions of information flow, from DR nodes to β-lactam^s^ nodes and vice versa. We ranked the access times between all pairs of nodes in each database separately and used bivariate Spearman’s ρ to calculate the concordance of edges with similar access times (38, 39) (see Fig. S4). We found that consistency between the PPI and GR networks occurred in the top 29.65% of edges, with the smallest access times in the β-lactam^s^-to-DR node direction and just 9.46% of those in the DR-to-β-lactam^s^ node direction. Similar results were obtained when we repeated the analysis with only the high-confidence β-lactam^s^ nodes, with the consistency in the β-lactam^s^-to-DR node direction being 31.09% and no consistency identified in the DR-to-β-lactam^s^ node direction. This result is consistent with the notion that PPI networks and GR networks represent different types of associations between genes and proteins. Therefore, we used PPI and GR networks separately for subsequent random walk analyses.

10.1128/mSphere.00245-21.4 FIG S4 Stability scores of access times obtained by random walks in the gene regulatory and protein-protein interaction networks from drug resistance (DR) nodes to β-lactam susceptibility (β-lactam^s^) nodes (A) and from β-lactam^s^ nodes to DR nodes (B). A drop in the stability scores indicates loss of rank consistency (vertical blue lines). This occurred in rank 272/3,836 in the DR-to-β-lactam^s^ direction, and from rank 1,146/3,836 in the β-lactam^s^-to-DR direction, indicating that the networks represent different types of interactions. Download FIG S4, DOCX file, 0.1 MB. Copyright © 2021 Trigos et al. 2021 Trigos et al. https://creativecommons.org/licenses/by/4.0/ This content is distributed under the terms of the Creative Commons Attribution 4.0 International license.

**FIG 5:**
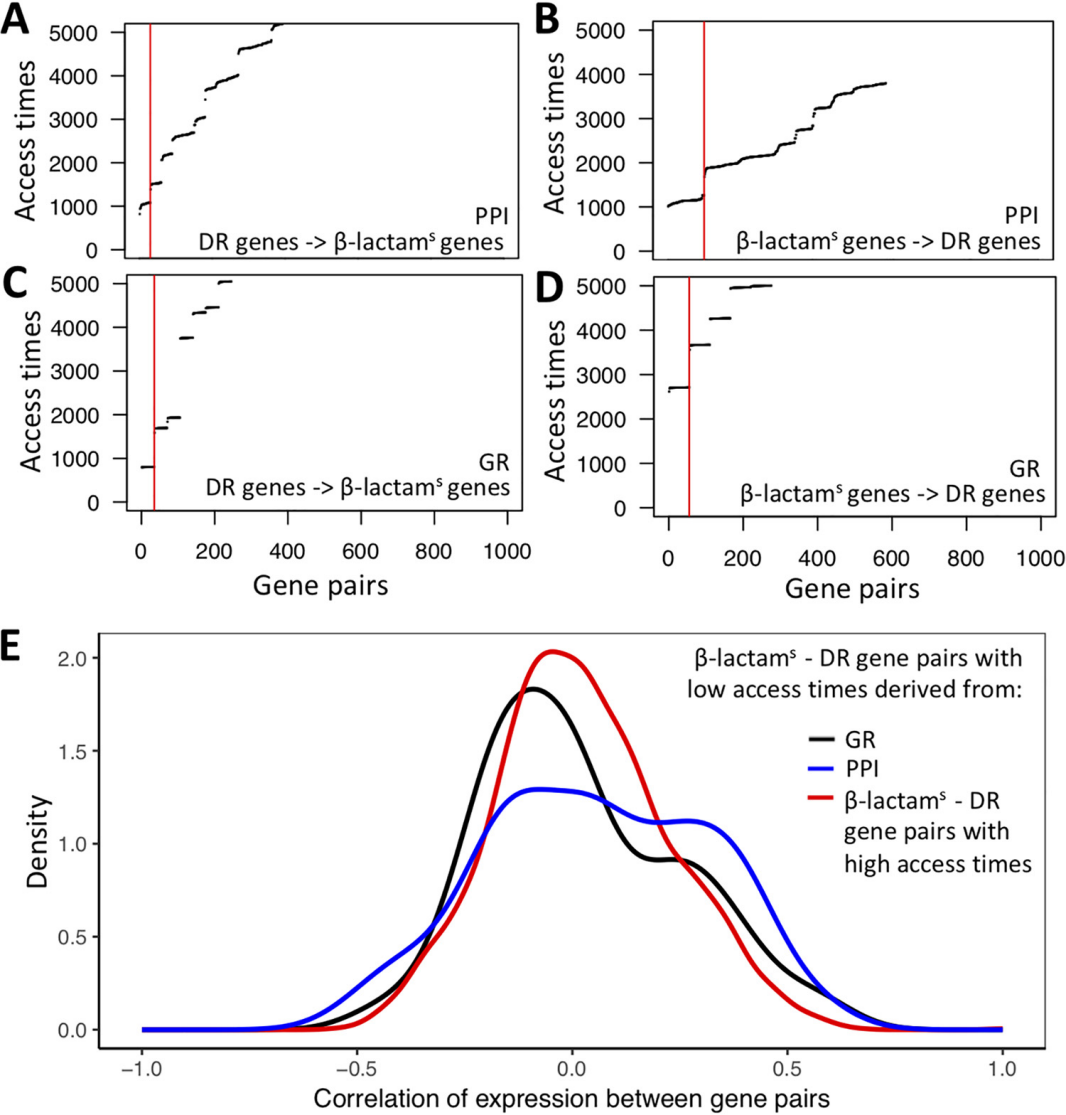
Highly influential pairs of β-lactam sensitivity (β-lactam^s^) and drug resistance (DR) nodes identified by random walks in the networks. Access times for gene pairs in the protein-protein interaction (PPI) (A and B) and gene regulatory (GR) (C and D) networks in the DR→β-lacam^s^ direction (A and C) and β-lactam^s^→DR direction (B and D). Due to a modular network structure, discrete clusters of highly influential pairs are identified. The set of genes with smallest access times (high influence between each other) to the left of the vertical red lines were used in the subsequent analysis. (E) Strength of coexpression between DR-β-lactam^s^ node pairs. DR-β-lactam^s^ node pairs with lowest access times are more strongly correlated than pairs of genes with higher access times (wider distribution).

Ranking pairs of β-lactam^s^ and DR nodes by their access times revealed discrete sets of node pairs with similar influence (Fig. 5A to D), consistent with the modular organization of the PPI and GR networks (40). We selected the set of node pairs with the smallest access times based on their distribution (red lines define threshold for each case): 30 and 35 pairs were identified from the PPI and GR networks, respectively, in the DR-to-β-lactam^s^ direction and 98 and 55 pairs, respectively, in the opposite direction (see Table S5). These node sets represent pairs of β-lactam^s^ and DR genes/proteins that are likely to modulate or influence each other’s activity.

10.1128/mSphere.00245-21.9 TABLE S5 Access times of genes of all pairs of DR and β-lactam^s^ genes. Download Table S5, DOCX file, 0.1 MB. Copyright © 2021 Trigos et al. 2021 Trigos et al. https://creativecommons.org/licenses/by/4.0/ This content is distributed under the terms of the Creative Commons Attribution 4.0 International license.

To ensure that the set of gene pairs with smallest access times represented biologically meaningful associations, we compared the coexpression of DR-β-lactam^s^ gene pairs with the smallest access times to DR-β-lactam^s^ gene pairs with higher access times (Fig. 5E). The distribution of coexpression of DR-β-lactam^s^ gene pairs with the smallest access times was wider than the reference distribution (Kolmogorov-Smirnov test *P* = 0.016 for the pairs derived from the PPI network and *P* = 0.021 for the pairs identified in the GR network), with a similar trend for β-lactam^s^-DR gene pairs (*P* = 0.063 in the PPI network and *P* = 0.08 in the GR network). This indicates stronger magnitudes of coexpression between gene pairs with smaller access times.

Examination of DR-β-lactam^s^ gene pairs with the smallest access times revealed two key nodes in the paths of information flow. All small access time pairs derived from the PPI network were centered around AtpH (encoded by *atpH*, *Rv1307*), and those derived from the GR network were centered around WhiB4 (encoded by *Rv3681c*). *atpH* is transcriptionally regulated by BlaI (30); hence, this result once more implicates *blaI* and its transcriptional network in M. tuberculosis β-lactam collateral sensitivity. WhiB4 is a transcription factor involved in the regulation of a large number of PE/PPE genes (41) and has been implicated in the response to β-lactam/β-lactam inhibitor combination efficacy in M. tuberculosis through its role in modulating internal redox potential (23).

### *In silico* functional validation of a dependence mechanism between β-lactam^s^ and DR gene pairs.

**FIG 6:**
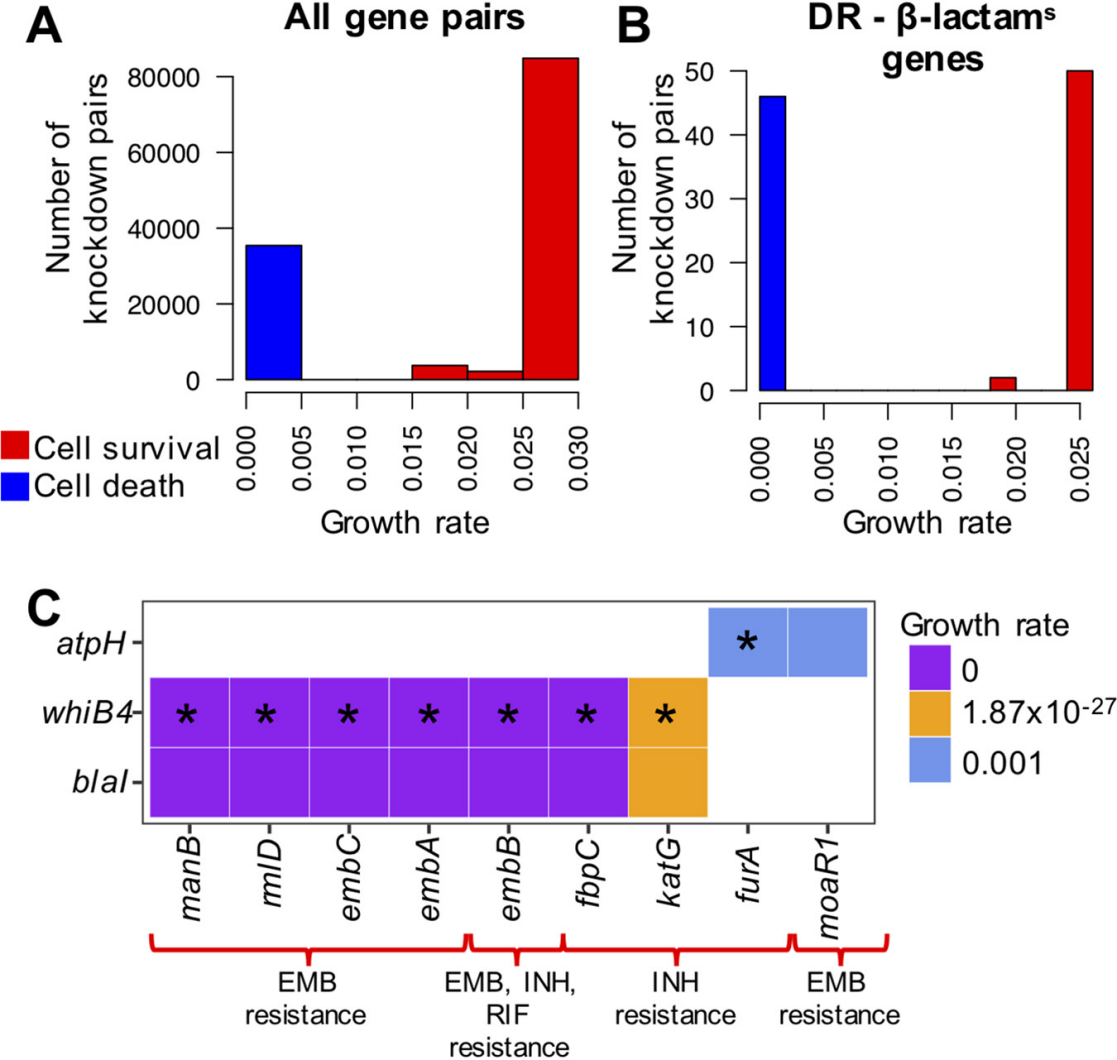
*In silico* double knockout of drug resistance (DR) and β-lactam sensitivity (β-lactam^s^) gene pairs reduces M. tuberculosis growth rate. Effect on the growth of M. tuberculosis after *in silico* knockout of all gene pairs (A) or DR-β-lactam^s^ gene pairs (B). DR-β-lactam^s^ gene pairs are enriched in those that lead to lethality (growth rate < 0.010) after knockout (Fisher test *P* = 7.00 × 10^−5^). Knockouts resulting in cells with a growth rate of <0.010 were considered lethal (blue), and above this cutoff, nonlethal (red). (C). Growth rate of pairs of β-lactam^s^ and DR genes after double knockouts. The knockouts of *atpH*, *whiB4*, and *blaI* together with DR genes implicated in the resistance to commonly used anti-TB drugs (e.g., EMB and INH) led to cell death. Gene pairs identified by random walk network analyses as being highly influential pairs are indicated with an asterisk. EMB, ethambutol; INH, isoniazid; RIF, rifampicin.

We sought to validate the functional association between β-lactam^s^ and DR genes/proteins by exploring their role in cell growth. We simulated the growth effects of β-lactam^s^ and DR gene pair knockouts using an *in silico* regulatory model that incorporates both transcriptional data and metabolic modeling (42). We found that simultaneous knockout of DR and β-lactam^s^ gene pairs caused a marked reduction in growth rate (growth rate < 0.010) or resulted in cell death more often than expected by chance (Fig. 6A and B) (88.46% β-lactam^s^ and DR gene pairs compared to only 39.0% in other pairs, Fisher exact test *P* = 7.00 × 10^−5^), suggesting synthetic lethality and functional dependency between these genes. Of note, we found synthetic lethality between each of the *whiB4*, *blaI*, and *atpH* genes with the key DR genes *embB*, *katG*, and *furA* (Fig. 6C).

## DISCUSSION

In this work, we applied a novel combination of systems biology approaches to investigate M. tuberculosis β-lactam collateral sensitivity. We combined gene expression and network analyses and show that the inhibitor of intrinsic β-lactam resistance, *blaI*, is activated after treatment with classical anti-TB drugs (e.g., isoniazid, rifampicin, amikacin, streptomycin, levofloxacin, ofloxacin, ethambutol, ethionamide, and pyrazinamide). Two genes transcriptionally regulated by *blaI*, *atpH* and *sigC* (30), as well as *Rv1884c* (*rpfC*), whose knockout mutants suffer increased sensitivity to β-lactams (31), were also upregulated. These findings are concordant with a model whereby classical anti-TB treatment drives cells toward a loss of β-lactam resistance, consistent with previous reports that drug-resistant M. tuberculosis strains were more likely to be susceptible to β-lactams (18, 19). Our gene expression analyses showed that DR and β-lactam^s^ genes were coexpressed, and our network analyses (transcription factor-target associations and PPI network analysis) indicate that this coexpression may result from a tight coregulatory association between DR and β-lactam^s^ genes. However, further experimental evidence would be needed to confirm the latter result, such as investigating the activity of *blaC* in multidrug-resistant and extensive drug-resistant strains. While one previous study indicated that β-lactamase activity was qualitatively conserved among 10 M. tuberculosis strains with various amoxicillin/clavulanate MICs (18), it remains unclear if there are quantitative differences in expression that may impact susceptibility.

Previous studies have demonstrated the utility of random walks across networks to identify putative treatment cotargets for M. tuberculosis (24). Here, we applied random walks to identify key mediators of the communication between DR and β-lactam^s^ genes and identified *atpH* and *sigC* as key regulators. In addition, *in silico* growth models revealed synthetic lethality after simultaneous knockout of either *blaI*, *atpH*, or *sigC* in combination with the genes conferring resistance to isoniazid, ethambutol, or rifampicin, further supporting a functional association between these gene classes.

The analysis conducted in this work primarily focused on a set of 63 high-confidence β-lactam^s^ genes, i.e., those with experimental evidence demonstrating an association with β-lactam sensitivity in M. tuberculosis. To ensure that our results were robust to changes in this set of genes, we repeated all analyses using an extended list of 199 β-lactam^s^-associated genes, including genes that were associated only through computational approaches (see Table S6 in the supplemental material). We find that all trends remain consistent, providing greater confidence that our analyses are robust to changes in the underlying list of genes.

Studies have shown that pathways regulating cell wall formation and β-lactam-associated genes are affected by stress in M. tuberculosis (43, 44), which would suggest that the induction of β-lactam^s^ gene expression and the subsequent acquisition of β-lactam susceptibility could be the result of a generic stress response. Our differential expression analysis did not reveal a preferential induction of β-lactam^s^ genes under acidic stress. In contrast, drug treatment was a more specific inducer of β-lactam^s^ genes, with 18.52% of induced genes being β-lactam^s^ genes. This result is consistent with the tight regulatory links between β-lactam^s^ and DR genes implicated by our network permutation and random walk analyses and *in silico* growth simulations. However, other types of stress, such as that induced by reactive oxygen species and reactive nitrogen species and macrophage stress, should also be investigated to rule out their role in the upregulation of β-lactam^s^ genes, and studies have reported regulation of β-lactam genes by the oxidant cumene hydroperoxide (23). Although we cannot exclude that some sources of stress might induce a response that modulates β-lactam susceptibility due to its effect on pathways regulating cell wall formation, our results as a whole suggest a potentially direct and specific rather than passive association between drug treatment and the activation of β-lactam^s^ genes.

Our *in silico* study of knockouts of gene pairs suggested synthetic lethality and functional dependency between β-lactam^s^ and DR gene pairs, specifically between each of the *whiB4*, *blaI*, and *atpH* genes with the key DR genes *embB*, *katG*, and *furA*. The effect of *whiB4* mutations and gene expression alterations has been studied in the context of single-gene knockouts or overexpression of *whiB4* (23, 45–47), suggesting a modulatory role in response to classical TB antibiotics. However, experimental studies are required to investigate the effect of double knockouts involving β-lactam^s^ and DR genes, which may be otherwise difficult to predict on the basis of single-gene knockout data.

Our findings are consistent with a model of collateral β-lactam sensitivity in classical drug-resistant M. tuberculosis, involving a concerted effect of multiple genes. Others have also recently found that collateral sensitivity to β-lactams, mainly penicillins, develops in M. tuberculosis strains evolved *in vitro* to be resistant to the classical drug class aminoglycosides (20). Our results suggest that *blaI*, together with its downstream targets, *atpH* and *sigC*, is a key regulator of collateral sensitivity resulting from classical drug resistance, although we were not able to detect a direct effect on transcription of the *blaC* β-lactamase gene in these data. Nevertheless, our evidence supporting a strong transcriptional wiring between β-lactam^s^ genes and DR genes suggests a tight coevolutionary relationship, likely due in part to functional similarities between the genes, many of which are implicated in resistance to drugs that target M. tuberculosis cell wall biosynthesis, e.g., ethambutol and isoniazid (48). Thus, collateral sensitivity to β-lactams may represent a functional evolutionary trade-off to classical drug resistance.

The development of bacterial drug resistance is often accompanied by a fitness cost (49), which in some cases can be overcome by compensatory mechanisms. We speculate that β-lactam sensitivity arises in M. tuberculosis as a compensatory mechanism to regain fitness after disruption of the molecular network of M. tuberculosis due to the evolution of classical drug resistance. Indeed, genes associated with sensitivity to β-lactams (e.g., *murE*, *ponA1*, *murD*, *Rv2752c*, and *Rv1218c*) have been identified as being under convergent evolution in drug-resistant M. tuberculosis or harboring compensatory mutations (50–52). Although most studies have associated compensatory mechanisms with mutations (50–52), our results suggest that transcriptional changes might also be playing a role, e.g., the upregulation of *blaI*. This assertion is consistent with a recent report showing that gene expression changes were associated with an increased fitness in M. tuberculosis that had developed resistance to rifampicin, isoniazid, streptomycin, fluoroquinolone, ethionamide, and amikacin during a single patient infection (53).

Taken together, our findings support a potential role for β-lactam therapy in patients with classical drug-resistant TB to delay and/or prevent the development of resistance. Previous *in vitro* studies have demonstrated anti-TB activity for certain β-lactam plus β-lactamase inhibitor combinations (10) and other drugs (54). However, mixed success in the clinic (15, 16, 55) suggests that treatment effectiveness might be dependent on other factors, such as the genetic background of the M. tuberculosis strain. Consequently, it will be essential to continue to develop our understanding of this phenomenon using a combination of bioinformatic and experimental approaches, such that we can readily identify M. tuberculosis strains and therefore patients for whom β-lactam therapy may be appropriate.

## MATERIALS AND METHODS

### Genes associated with β-lactam sensitivity.

A list of 199 genes putatively associated with β-lactam sensitivity in M. tuberculosis, and two closely related species, M. smegmatis and M. bovis, was obtained from multiple sources (17, 18, 23, 30, 31, 56–76). An initial set of 110 genes as reported in reference 18 was extended to 199 by further literature searches (see Table S1 in the supplemental material). These included a diverse set of discovery approaches (e.g., functional studies focused on single genes, high-throughput phenotypic screens, and computational analyses) with variable likelihood of spurious or false-positive results. To limit the potential influence of false-positive genes, we report separate analyses for all 199 genes and a subset of 63 genes with the highest confidence evidence (i.e., which included canonical genes such as *blaI*, *bla*, and *atpA-G* that were shown to have β-lactamase activity, are downstream of *blaI*, or were identified through functional assays as being associated with sensitivity to β-lactams). A full list of genes used in this study can be found in Table S1 (genes in the high-confidence subset are marked).

### Genes implicated in resistance to classical TB drugs.

We compiled a list of 40 genes implicated in classical TB drug resistance (here termed DR genes) from The Tuberculosis Drug Resistance Mutation Database (81) (Table S2). These included genes associated with resistance to rifampicin (RIF; *n* = 2), isoniazid (INH; *n* = 22), aminoglycosides (AMI; kanamycin, capreomycin, amikacin, and viomycin, *n* = 2), streptomycin (SM; *n* = 3), fluoroquinolones (FLQs; *n* = 2), ethambutol (EMB; *n* = 13), ethionamide (ETH; *n* = 3), *para*-aminosalicylic acid (PAS; *n* = 1), and pyrazinamide (PZA; *n* = 1).

### Expression data.

M. tuberculosis microarray gene expression data were obtained from sample series GSE1642 (26) from the NCBI GEO database. Data were available for M. tuberculosis exposed to 437 treatments, including the following *in vitro* treatment conditions: classical TB drugs as single agents (isoniazid, rifampin, amikacin, streptomycin, levofloxacin, ofloxacin, ethambutol, ethionamide, and pyrazinamide) and control conditions (7H9-based growth medium without drug treatment).

We assessed the impact of classical drug treatment by comparing the variance of expression of β-lactam^s^ genes to that across all genes. Significance testing was performed by comparison to the null distribution generated by random subsampling of M. tuberculosis genes (*n* = 111 genes with 10,000 replicates) and counting the number of times we obtained a variance equal to or greater than the observed value. Differential expression analysis was performed using limma (27), where differential expression was considered significant if the *q* value (i.e., a *P* value that has been adjusted for the false-discovery rate [FDR] considering multiple testing) was <0.05 and |fold change| was >2.

To compare the strength of correlation of expression of DR genes with β-lactam^s^ genes, we exhaustively calculated Spearman’s ρ between the expression of each of the individual genes, generating (i) a distribution of correlations of each individual DR gene with all β-lactam^s^ genes, and (ii) a distribution of DR genes with all other genes. We then used the upper quantile of the correlation magnitude (absolute value of the correlation of expression) of each of these distributions to summarize the differences in the distribution of the strength of correlation magnitude, therefore comparing the most strongly correlated DR genes and β-lactam^s^ genes with the most strongly correlated DR genes and non-β-lactam^s^ genes.

### M. tuberculosis molecular interaction networks.

We integrated molecular interaction networks from two sources: protein-protein interactions (PPIs) (22,308 interactions) from the STRING database (32) and transcription factor-target interactions experimentally obtained using chromatin immunoprecipitations (33) as a gene regulatory (GR) network (15,054 interactions). Note that although the STRING database has traditionally been considered to be solely composed of PPIs, there are a number of regulatory interactions supported by gene coexpression analysis (32). Only high-confidence edges were analyzed: PPIs with a weight greater than 700 (the cutoff suggested by STRING as being of high confidence) and statistically significant transcription factor-target gene interactions (as defined by reference 33) were considered. The power-law distribution of the combined network PPI and GR was verified using igraph (77) to ensure its biological plausibility. Network visualizations were obtained using Cytoscape v3.4.0 (78).

### Significance of interactions between β-lactam^s^ and DR nodes (genes/proteins) in the molecular interaction network.

The degree of cross talk, *N_AB_*, between two gene sets, *A* and *B* (e.g., β-lactam^s^ and DR genes), expected under a random model of a given network was modeled using the hypergeometric distribution *N_AB_* ≈ hypergeometric(*N*, *K*, *n*), where *n*, *K*, and *N* are the numbers of edges in gene set *A*, gene set *B*, and the entire network (35). A one-sided *P* value can be calculated as the probability of observing at least the observed number of interactions under this random model. Significance was corrected using the Benjamini-Hochberg method (and hence is reported as *q* values).

### Random network walks to identify β-lactam^s^ nodes influenced by DR nodes.

We performed random walks between all pairs of nodes in the PPI and the GR networks separately to determine the access times as an indicator for the influence of one node over another. Simulating the random walk was unnecessary, as the access time on a finite graph has an analytical solution (79) computed via eigenvalue decomposition of the edge matrices of the networks.

To assess the similarity of the access times obtained with the PPI (32) and the GR networks (33), we investigated the stability using a multivariate extension of Spearman’s ρ (38, 39). This allows us to assess the similarity of the top-*k* access times and determine if there is a set of stable edges with low access time.

We selected pairs of nodes comprising one β-lactam^s^ node and one DR node and narrowed down sets of pairs with small access times in either the PPI or GR network. Given the nonsymmetry of access times obtained with random walks (the access time from A to B is not equal to that from B to A), we considered the results obtained in both directions independently. Cutoffs were determined from the empirical distribution: 1,054.74 for the PPI in the DR gene→β-lactam^s^ gene direction, 1,336.58 for the β-lactam^s^ gene→DR gene direction, 1,713.37 for the GR network in the DR gene→β-lactam^s^ gene direction, and 2,741.49 in the β-lactam^s^ gene→DR gene direction.

### Simulating the effect on bacterial growth of double knockout β-lactam^s^ plus DR gene mutants.

To identify pairs of β-lactam^s^ and DR genes whose knockout would have the largest effect on the growth of M. tuberculosis, we performed simulations using the iSM810 model of M. tuberculosis with the PROM framework (42) and the COBRA toolbox (80), which incorporate both gene-regulatory and metabolic processes to predict growth rates after double knockdown simulations. As input, we used the GR network (33) and expression data described above (26).

## References

[1] Pal C, Papp B, Lazar V. 2015. Collateral sensitivity of antibiotic-resistant microbes. Trends Microbiol 23:401–407. doi:10.1016/j.tim.2015.02.009. 25818802 PMC5958998

[2] Imamovic L, Sommer MO. 2013. Use of collateral sensitivity networks to design drug cycling protocols that avoid resistance development. Sci Transl Med 5:204ra132. doi:10.1126/scitranslmed.3006609. 24068739

[3] Oz T, Guvenek A, Yildiz S, Karaboga E, Tamer YT, Mumcuyan N, Ozan VB, Senturk GH, Cokol M, Yeh P, Toprak E. 2014. Strength of selection pressure is an important parameter contributing to the complexity of antibiotic resistance evolution. Mol Biol Evol 31:2387–2401. doi:10.1093/molbev/msu191. 24962091 PMC4137714

[4] Wong A. 2017. Epistasis and the evolution of antimicrobial resistance. Front Microbiol 8:246. doi:10.3389/fmicb.2017.00246. 28261193 PMC5313483

[5] World Health Organization. 2020. Global tuberculosis report. World Health Organization, Geneva, Switzerland.

[6] Keener AB. 2014. Oldie but goodie: repurposing penicillin for tuberculosis. Nat Med 20:976–978. doi:10.1038/nm0914-976. 25198041

[7] Kumar P, Kaushik A, Lloyd EP, Li SG, Mattoo R, Ammerman NC, Bell DT, Perryman AL, Zandi TA, Ekins S, Ginell SL, Townsend CA, Freundlich JS, Lamichhane G. 2017. Non-classical transpeptidases yield insight into new antibacterials. Nat Chem Biol 13:54–61. doi:10.1038/nchembio.2237. 27820797 PMC5477059

[8] Sauvage E, Kerff F, Terrak M, Ayala JA, Charlier P. 2008. The penicillin-binding proteins: structure and role in peptidoglycan biosynthesis. FEMS Microbiol Rev 32:234–258. doi:10.1111/j.1574-6976.2008.00105.x. 18266856

[9] Gupta R, Lavollay M, Mainardi JL, Arthur M, Bishai WR, Lamichhane G. 2010. The *Mycobacterium tuberculosis* protein LdtMt2 is a nonclassical transpeptidase required for virulence and resistance to amoxicillin. Nat Med 16:466–469. doi:10.1038/nm.2120. 20305661 PMC2851841

[10] Hugonnet JE, Tremblay LW, Boshoff HI, Barry CE, III, Blanchard JS. 2009. Meropenem-clavulanate is effective against extensively drug-resistant *Mycobacterium tuberculosis*. Science 323:1215–1218. doi:10.1126/science.1167498. 19251630 PMC2679150

[11] Hugonnet JE, Blanchard JS. 2007. Irreversible inhibition of the *Mycobacterium tuberculosis* beta-lactamase by clavulanate. Biochemistry 46:11998–12004. doi:10.1021/bi701506h. 17915954 PMC2593862

[12] Dhar N, Dubee V, Ballell L, Cuinet G, Hugonnet JE, Signorino-Gelo F, Barros D, Arthur M, McKinney JD. 2015. Rapid cytolysis of *Mycobacterium tuberculosis* by faropenem, an orally bioavailable beta-lactam antibiotic. Antimicrob Agents Chemother 59:1308–1319. doi:10.1128/AAC.03461-14. 25421469 PMC4335862

[13] Kaushik A, Makkar N, Pandey P, Parrish N, Singh U, Lamichhane G. 2015. Carbapenems and rifampin exhibit synergy against *Mycobacterium tuberculosis* and *Mycobacterium abscessus*. Antimicrob Agents Chemother 59:6561–6567. doi:10.1128/AAC.01158-15. 26259792 PMC4576034

[14] Jaganath D, Lamichhane G, Shah M. 2016. Carbapenems against *Mycobacterium tuberculosis*: a review of the evidence. Int J Tuber Lung Dis 20:1436–1447. doi:10.5588/ijtld.16.0498. 27776583

[15] Donald PR, Sirgel FA, Venter A, Parkin DP, Van de Wal BW, Barendse A, Smit E, Carman D, Talent J, Maritz J. 2001. Early bactericidal activity of amoxicillin in combination with clavulanic acid in patients with sputum smear-positive pulmonary tuberculosis. Scand J Infect Dis 33:466–469. doi:10.1080/00365540152029954. 11450868

[16] Chambers HF, Kocagoz T, Sipit T, Turner J, Hopewell PC. 1998. Activity of amoxicillin/clavulanate in patients with tuberculosis. Clin Infect Dis 26:874–877. doi:10.1086/513945. 9564467

[17] Kumar P, Kaushik A, Bell DT, Chauhan V, Xia F, Stevens RL, Lamichhane G. 2017. Mutation in an unannotated protein confers carbapenem resistance in *Mycobacterium tuberculosis*. Antimicrob Agents Chemother 61:e02234-16. doi:10.1128/AAC.02234-16. 28069655 PMC5328524

[18] Cohen KA, El-Hay T, Wyres KL, Weissbrod O, Munsamy V, Yanover C, Aharonov R, Shaham O, Conway TC, Goldschmidt Y, Bishai WR, Pym AS. 2016. Paradoxical hypersusceptibility of drug-resistant *Mycobacterium tuberculosis* to beta-lactam antibiotics. EBioMedicine 9:170–179. doi:10.1016/j.ebiom.2016.05.041. 27333036 PMC4972527

[19] Payen MC, De Wit S, Martin C, Sergysels R, Muylle I, Van Laethem Y, Clumeck N. 2012. Clinical use of the meropenem-clavulanate combination for extensively drug-resistant tuberculosis. Int J Tuber Lung Dis 16:558–560. doi:10.5588/ijtld.11.0414. 22325421

[20] Barbosa C, Trebosc V, Kemmer C, Rosenstiel P, Beardmore R, Schulenburg H, Jansen G. 2017. Alternative evolutionary paths to bacterial antibiotic resistance cause distinct collateral effects. Mol Biol Evol 34:2229–2244. doi:10.1093/molbev/msx158. 28541480 PMC5850482

[21] Sambarey A, Prashanthi K, Chandra N. 2013. Mining large-scale response networks reveals 'topmost activities' in *Mycobacterium tuberculosis* infection. Sci Rep 3:2302. doi:10.1038/srep02302. 23892477 PMC3725478

[22] Hegde SR, Rajasingh H, Das C, Mande SS, Mande SC. 2012. Understanding communication signals during mycobacterial latency through predicted genome-wide protein interactions and Boolean modeling. PLoS One 7:e33893. doi:10.1371/journal.pone.0033893. 22448278 PMC3309013

[23] Mishra S, Shukla P, Bhaskar A, Anand K, Baloni P, Jha RK, Mohan A, Rajmani RS, Nagaraja V, Chandra N, Singh A. 2017. Efficacy of β-lactam/β-lactamase inhibitor combination is linked to WhiB4-mediated changes in redox physiology of *Mycobacterium tuberculosis*. Elife 6:e25624. doi:10.7554/eLife.25624. 28548640 PMC5473688

[24] Chen LC, Yeh HY, Yeh CY, Arias CR, Soo VW. 2012. Identifying co-targets to fight drug resistance based on a random walk model. BMC Syst Biol 6:5. doi:10.1186/1752-0509-6-5. 22257493 PMC3296574

[25] Hwang S, Kim CY, Ji SG, Go J, Kim H, Yang S, Kim HJ, Cho A, Yoon SS, Lee I. 2016. Network-assisted investigation of virulence and antibiotic-resistance systems in *Pseudomonas aeruginosa*. Sci Rep 6:26223. doi:10.1038/srep26223. 27194047 PMC4872156

[26] Boshoff HI, Myers TG, Copp BR, McNeil MR, Wilson MA, Barry CE, 3rd, 2004. The transcriptional responses of *Mycobacterium tuberculosis* to inhibitors of metabolism: novel insights into drug mechanisms of action. J Biol Chem 279:40174–40184. doi:10.1074/jbc.M406796200. 15247240

[27] Ritchie ME, Phipson B, Wu D, Hu Y, Law CW, Shi W, Smyth GK. 2015. limma powers differential expression analyses for RNA-sequencing and microarray studies. Nucleic Acids Res 43:e47. doi:10.1093/nar/gkv007. 25605792 PMC4402510

[28] Rohde KH, Abramovitch RB, Russell DG. 2007. *Mycobacterium tuberculosis* invasion of macrophages: linking bacterial gene expression to environmental cues. Cell Host Microbe 2:352–364. doi:10.1016/j.chom.2007.09.006. 18005756

[29] Rustad TR, Harrell MI, Liao R, Sherman D. 2008. The enduring hypoxic response of *Mycobacterium tuberculosis*. PLoS One 3:e1502. doi:10.1371/journal.pone.0001502. 18231589 PMC2198943

[30] Sala C, Haouz A, Saul FA, Miras I, Rosenkrands I, Alzari PM, Cole ST. 2009. Genome-wide regulon and crystal structure of BlaI (Rv1846c) from *Mycobacterium tuberculosis*. Mol Microbiol 71:1102–1116. doi:10.1111/j.1365-2958.2008.06583.x. 19154333

[31] Wivagg CN, Hung DT. 2012. Resuscitation-promoting factors are required for beta-lactam tolerance and the permeability barrier in *Mycobacterium tuberculosis*. Antimicrob Agents Chemother 56:1591–1594. doi:10.1128/AAC.06027-11. 22155826 PMC3294900

[32] Szklarczyk D, Franceschini A, Wyder S, Forslund K, Heller D, Huerta-Cepas J, Simonovic M, Roth A, Santos A, Tsafou KP, Kuhn M, Bork P, Jensen LJ, von Mering C. 2015. STRING v10: protein-protein interaction networks, integrated over the tree of life. Nucleic Acids Res 43:D447–D452. doi:10.1093/nar/gku1003. 25352553 PMC4383874

[33] Minch KJ, Rustad TR, Peterson EJ, Winkler J, Reiss DJ, Ma S, Hickey M, Brabant W, Morrison B, Turkarslan S, Mawhinney C, Galagan JE, Price ND, Baliga NS, Sherman DR. 2015. The DNA-binding network of *Mycobacterium tuberculosis*. Nat Commun 6:5829. doi:10.1038/ncomms6829. 25581030 PMC4301838

[34] Barabasi AL, Oltvai ZN. 2004. Network biology: understanding the cell's functional organization. Nat Rev Genet 5:101–113. doi:10.1038/nrg1272. 14735121

[35] Signorelli M, Vinciotti V, Wit EC. 2016. NEAT: an efficient network enrichment analysis test. BMC Bioinformatics 17:352. doi:10.1186/s12859-016-1203-6. 27597310 PMC5011912

[36] Ravasz E, Somera AL, Mongru DA, Oltvai ZN, Barabasi AL. 2002. Hierarchical organization of modularity in metabolic networks. Science 297:1551–1555. doi:10.1126/science.1073374. 12202830

[37] Tanay A, Sharan R, Kupiec M, Shamir R. 2004. Revealing modularity and organization in the yeast molecular network by integrated analysis of highly heterogeneous genomewide data. Proc Natl Acad Sci U S A 101:2981–2986. doi:10.1073/pnas.0308661100. 14973197 PMC365731

[38] Bedő J, Rawlinson D, Goudey B, Ong CS. 2014. Stability of bivariate GWAS biomarker detection. PLoS One 9:e93319. doi:10.1371/journal.pone.0093319. 24787002 PMC4005767

[39] Bedő J, Ong C. 2016. Multivariate Spearman’s ρ for aggregating ranks using copulas. J Mach Learn Res 17:1–30.

[40] Maslov S, Sneppen K. 2002. Specificity and stability in topology of protein networks. Science 296:910–913. doi:10.1126/science.1065103. 11988575

[41] Wu J, Ru HW, Xiang ZH, Jiang J, Wang YC, Zhang L, Liu J. 2017. WhiB4 regulates the PE/PPE gene family and is essential for virulence of *Mycobacterium marinum*. Sci Rep 7:3007. doi:10.1038/s41598-017-03020-4. 28592799 PMC5462746

[42] Chandrasekaran S, Price ND. 2010. Probabilistic integrative modeling of genome-scale metabolic and regulatory networks in *Escherichia coli* and *Mycobacterium tuberculosis*. Proc Natl Acad Sci U S A 107:17845–17850. doi:10.1073/pnas.1005139107. 20876091 PMC2955152

[43] Stallings CL, Glickman MS. 2010. Is *Mycobacterium tuberculosis* stressed out? A critical assessment of the genetic evidence. Microbes Infect 12:1091–1101. doi:10.1016/j.micinf.2010.07.014. 20691805 PMC3587153

[44] Zeng X, Lin J. 2013. Beta-lactamase induction and cell wall metabolism in Gram-negative bacteria. Front Microbiol 4:128. doi:10.3389/fmicb.2013.00128. 23734147 PMC3660660

[45] Chawla M, Parikh P, Saxena A, Munshi M, Mehta M, Mai D, Srivastava AK, Narasimhulu KV, Redding KE, Vashi N, Kumar D, Steyn AJ, Singh A. 2012. *Mycobacterium tuberculosis* WhiB4 regulates oxidative stress response to modulate survival and dissemination *in vivo*. Mol Microbiol 85:1148–1165. doi:10.1111/j.1365-2958.2012.08165.x. 22780904 PMC3438311

[46] Ma S, Minch KJ, Rustad TR, Hobbs S, Zhou S-L, Sherman DR, Price ND. 2015. Integrated modeling of gene regulatory and metabolic networks in *Mycobacterium tuberculosis*. PLoS Comput Biol 11:e1004543. doi:10.1371/journal.pcbi.1004543. 26618656 PMC4664399

[47] Chawla M, Mishra S, Anand K, Parikh P, Mehta M, Vij M, Verma T, Singh P, Jakkala K, Verma HN, AjitKumar P, Ganguli M, Narain Seshasayee AS, Singh A. 2018. Redox-dependent condensation of the mycobacterial nucleoid by WhiB4. Redox Biol 19:116–133. doi:10.1016/j.redox.2018.08.006. 30149290 PMC6111044

[48] Almeida Da Silva PE, Palomino JC. 2011. Molecular basis and mechanisms of drug resistance in *Mycobacterium tuberculosis*: classical and new drugs. J Antimicrob Chemother 66:1417–1430. doi:10.1093/jac/dkr173. 21558086

[49] Gagneux S. 2009. Fitness cost of drug resistance in *Mycobacterium tuberculosis*. Clin Microbiol Infect 15 Suppl 1:66–68. doi:10.1111/j.1469-0691.2008.02685.x. 19220360

[50] Comas I, Borrell S, Roetzer A, Rose G, Malla B, Kato-Maeda M, Galagan J, Niemann S, Gagneux S. 2011. Whole-genome sequencing of rifampicin-resistant *Mycobacterium tuberculosis* strains identifies compensatory mutations in RNA polymerase genes. Nat Genet 44:106–110. doi:10.1038/ng.1038. 22179134 PMC3246538

[51] Farhat MR, Shapiro BJ, Kieser KJ, Sultana R, Jacobson KR, Victor TC, Warren RM, Streicher EM, Calver A, Sloutsky A, Kaur D, Posey JE, Plikaytis B, Oggioni MR, Gardy JL, Johnston JC, Rodrigues M, Tang PK, Kato-Maeda M, Borowsky ML, Muddukrishna B, Kreiswirth BN, Kurepina N, Galagan J, Gagneux S, Birren B, Rubin EJ, Lander ES, Sabeti PC, Murray M. 2013. Genomic analysis identifies targets of convergent positive selection in drug-resistant *Mycobacterium tuberculosis*. Nat Genet 45:1183–1189. doi:10.1038/ng.2747. 23995135 PMC3887553

[52] Zhang H, Li D, Zhao L, Fleming J, Lin N, Wang T, Liu Z, Li C, Galwey N, Deng J, Zhou Y, Zhu Y, Gao Y, Wang T, Wang S, Huang Y, Wang M, Zhong Q, Zhou L, Chen T, Zhou J, Yang R, Zhu G, Hang H, Zhang J, Li F, Wan K, Wang J, Zhang XE, Bi L. 2013. Genome sequencing of 161 *Mycobacterium tuberculosis* isolates from China identifies genes and intergenic regions associated with drug resistance. Nat Genet 45:1255–1260. doi:10.1038/ng.2735. 23995137

[53] Eldholm V, Norheim G, von der Lippe B, Kinander W, Dahle UR, Caugant DA, Mannsaker T, Mengshoel AT, Dyrhol-Riise AM, Balloux F. 2014. Evolution of extensively drug-resistant *Mycobacterium tuberculosis* from a susceptible ancestor in a single patient. Genome Biol 15:490. doi:10.1186/s13059-014-0490-3. 25418686 PMC4223161

[54] Ramon-Garcia S, Gonzalez Del Rio R, Villarejo AS, Sweet GD, Cunningham F, Barros D, Ballell L, Mendoza-Losana A, Ferrer-Bazaga S, Thompson CJ. 2016. Repurposing clinically approved cephalosporins for tuberculosis therapy. Sci Rep 6:34293. doi:10.1038/srep34293. 27678056 PMC5039641

[55] Tiberi S, D'Ambrosio L, De Lorenzo S, Viggiani P, Centis R, Sotgiu G, Alffenaar JW, Migliori GB. 2016. Ertapenem in the treatment of multidrug-resistant tuberculosis: first clinical experience. Eur Respir J 47:333–336. doi:10.1183/13993003.01278-2015. 26585427

[56] Singh G, Kumar A, Arya S, Gupta UD, Singh K, Kaur J. 2016. Characterization of a novel esterase Rv1497 of *Mycobacterium tuberculosis* H37Rv demonstrating beta-lactamase activity. Enzyme Microb Technol 82:180–190. doi:10.1016/j.enzmictec.2015.10.007. 26672466

[57] Dinesh N, Sharma S, Balganesh M. 2013. Involvement of efflux pumps in the resistance to peptidoglycan synthesis inhibitors in *Mycobacterium tuberculosis*. Antimicrob Agents Chemother 57:1941–1943. doi:10.1128/AAC.01957-12. 23335736 PMC3623302

[58] Flores AR, Parsons LM, Pavelka MS. 2005. Characterization of novel *Mycobacterium tuberculosis* and *Mycobacterium smegmatis* mutants hypersusceptible to β-lactam antibiotics. J Bacteriol 187:1892–1900. doi:10.1128/JB.187.6.1892-1900.2005. 15743935 PMC1064048

[59] Bhakta S, Basu J. 2002. Overexpression, purification and biochemical characterization of a class A high-molecular-mass penicillin-binding protein (PBP), PBP1* and its soluble derivative from Mycobacterium tuberculosis. Biochem J 361:635–639. doi:10.1042/0264-6021:3610635. 11802794 PMC1222347

[60] Qin L, Wang J, Zheng R, Lu J, Yang H, Liu Z, Cui Z, Jin R, Feng Y, Hu Z. 2011. Perspective on sequence evolution of microsatellite locus (CCG)n in *Rv0050* gene from *Mycobacterium tuberculosis*. BMC Evol Biol 11:247. doi:10.1186/1471-2148-11-247. 21878130 PMC3176237

[61] Cole ST, Brosch R, Parkhill J, Garnier T, Churcher C, Harris D, Gordon SV, Eiglmeier K, Gas S, Barry CE, III, Tekaia F, Badcock K, Basham D, Brown D, Chillingworth T, Connor R, Davies R, Devlin K, Feltwell T, Gentles S, Hamlin N, Holroyd S, Hornsby T, Jagels K, Krogh A, McLean J, Moule S, Murphy L, Oliver K, Osborne J, Quail MA, Rajandream MA, Rogers J, Rutter S, Seeger K, Skelton J, Squares R, Squares S, Sulston JE, Taylor K, Whitehead S, Barrell BG. 1998. Deciphering the biology of *Mycobacterium tuberculosis* from the complete genome sequence. Nature 393:537–544. doi:10.1038/31159. 9634230

[62] Munshi T, Gupta A, Evangelopoulos D, Guzman JD, Gibbons S, Keep NH, Bhakta S. 2013. Characterisation of ATP-dependent Mur ligases involved in the biogenesis of cell wall peptidoglycan in *Mycobacterium tuberculosis*. PLoS One 8:e60143. doi:10.1371/journal.pone.0060143. 23555903 PMC3605390

[63] Danilchanka O, Mailaender C, Niederweis M. 2008. Identification of a novel multidrug efflux pump of *Mycobacterium tuberculosis*. Antimicrob Agents Chemother 52:2503–2511. doi:10.1128/AAC.00298-08. 18458127 PMC2443884

[64] Dubée V, Triboulet S, Mainardi J-L, Ethève-Quelquejeu M, Gutmann L, Marie A, Dubost L, Hugonnet J-E, Arthur M. 2012. Inactivation of *Mycobacterium tuberculosis* l,d-transpeptidase Ldt_Mt1_ by carbapenems and cephalosporins. Antimicrob Agents Chemother 56:4189–4195. doi:10.1128/AAC.00665-12. 22615283 PMC3421625

[65] Fukuda T, Matsumura T, Ato M, Hamasaki M, Nishiuchi Y, Murakami Y, Maeda Y, Yoshimori T, Matsumoto S, Kobayashi K, Kinoshita T, Morita YS. 2013. Critical roles for lipomannan and lipoarabinomannan in cell wall integrity of mycobacteria and pathogenesis of tuberculosis. mBio 4:e00472-12. doi:10.1128/mBio.00472-12. 23422411 PMC3573661

[66] Kumar P, Arora K, Lloyd JR, Lee IY, Nair V, Fischer E, Boshoff HIM, Barry CE, III. 2012. Meropenem inhibits d,d-carboxypeptidase activity in *Mycobacterium tuberculosis*. Mol Microbiol 86:367–381. doi:10.1111/j.1365-2958.2012.08199.x. 22906310 PMC3468717

[67] Lun S, Miranda D, Kubler A, Guo H, Maiga MC, Winglee K, Pelly S, Bishai WR. 2014. Synthetic lethality reveals mechanisms of *Mycobacterium tuberculosis* resistance to β-lactams. mBio 5:e01767-14. doi:10.1128/mBio.01767-14. 25227469 PMC4172077

[68] McDonough JA, Hacker KE, Flores AR, Pavelka MS, Jr, Braunstein M. 2005. The twin-arginine translocation pathway of *Mycobacterium smegmatis* is functional and required for the export of mycobacterial beta-lactamases. J Bacteriol 187:7667–7679. doi:10.1128/JB.187.22.7667-7679.2005. 16267291 PMC1280313

[69] Smani Y, Fàbrega A, Roca I, Sánchez-Encinales V, Vila J, Pachón J. 2014. Role of OmpA in the multidrug resistance phenotype of *Acinetobacter baumannii*. Antimicrob Agents Chemother 58:1806–1808. doi:10.1128/AAC.02101-13. 24379205 PMC3957889

[70] Nampoothiri KM, Rubex R, Patel AK, Narayanan SS, Krishna S, Das SM, Pandey A. 2008. Molecular cloning, overexpression and biochemical characterization of hypothetical beta-lactamases of *Mycobacterium tuberculosis* H37Rv. J Appl Microbiol 105:59–67. doi:10.1111/j.1365-2672.2007.03721.x. 18217931

[71] Sun L, Zhang L, Zhang H, He Z-G. 2011. Characterization of a bifunctional β-lactamase/ribonuclease and its interaction with a chaperone-like protein in the pathogen *Mycobacterium tuberculosis* H37Rv. Biochemistry (Mosc) 76:350–358. doi:10.1134/s0006297911030096. 21568871

[72] Datta P, Dasgupta A, Singh AK, Mukherjee P, Kundu M, Basu J. 2006. Interaction between FtsW and penicillin-binding protein 3 (PBP3) directs PBP3 to mid-cell, controls cell septation and mediates the formation of a trimeric complex involving FtsZ, FtsW and PBP3 in mycobacteria. Mol Microbiol 62:1655–1673. doi:10.1111/j.1365-2958.2006.05491.x. 17427288

[73] Slayden RA, Belisle JT. 2009. Morphological features and signature gene response elicited by inactivation of FtsI in *Mycobacterium tuberculosis*. J Antimicrob Chemother 63:451–457. doi:10.1093/jac/dkn507. 19109339 PMC2721702

[74] Saint-Joanis B, Demangel C, Jackson M, Brodin P, Marsollier L, Boshoff H, Cole ST. 2006. Inactivation of Rv2525c, a substrate of the twin arginine translocation (Tat) system of *Mycobacterium tuberculosis*, increases beta-lactam susceptibility and virulence. J Bacteriol 188:6669–6679. doi:10.1128/JB.00631-06. 16952959 PMC1595485

[75] Plocinski P, Ziolkiewicz M, Kiran M, Vadrevu SI, Nguyen HB, Hugonnet J, Veckerle C, Arthur M, Dziadek J, Cross TA, Madiraju M, Rajagopalan M. 2011. Characterization of CrgA, a new partner of the *Mycobacterium tuberculosis* peptidoglycan polymerization complexes. J Bacteriol 193:3246–3256. doi:10.1128/JB.00188-11. 21531798 PMC3133284

[76] Usha V, Dover LG, Roper DI, Fütterer K, Besra GS. 2009. Structure of the diaminopimelate epimerase DapF from *Mycobacterium tuberculosis*. Acta Crystallogr D Biol Crystallogr 65:383–387. doi:10.1107/S0907444909002522. 19307721

[77] Csardi G, Nepusz T. 2006. The igraph software package for complex network research. InterJournal Complex Systems:1695.

[78] Shannon P, Markiel A, Ozier O, Baliga NS, Wang JT, Ramage D, Amin N, Schwikowski B, Ideker T. 2003. Cytoscape: a software environment for integrated models of biomolecular interaction networks. Genome Res 13:2498–2504. doi:10.1101/gr.1239303. 14597658 PMC403769

[79] Lovász L. 1993. Random walks on graphs: a survey. Combinatorics Paul Erdős is Eighty 2:1–46.

[80] Schellenberger J, Que R, Fleming RM, Thiele I, Orth JD, Feist AM, Zielinski DC, Bordbar A, Lewis NE, Rahmanian S, Kang J, Hyduke DR, Palsson BO. 2011. Quantitative prediction of cellular metabolism with constraint-based models: the COBRA toolbox v2.0. Nat Protoc 6:1290–1307. doi:10.1038/nprot.2011.308. 21886097 PMC3319681

[81] Sandgren A, Strong M, Muthukrishnan P, Weiner BK, Church GM, Murray MB. 2009. Tuberculosis drug resistance mutation database. PLoS Med 6:e1000002. doi:10.1371/journal.pmed.1000002. PMC2637921 19209951

