# Supplementary material for: Collateral Sensitivity to β-Lactam Drugs in Drug-Resistant Tuberculosis Is Driven by the Transcriptional Wiring of BlaI Operon Genes
Source: mSphere. 2021 May 28;6(3):e00245-21. doi: 10.1128/mSphere.00245-21 (PMC8265638; doi:10.1128/mSphere.00245-21)
Supplement: TABLE S1 [file msphere.00245-21-st001.docx]

**Supplementary Table S1.**

| **Locus** | **Gene_Name(s)** | **Product** | **Notes** | **Family** | **Reason** | **Reference** | **Confidence** |
| --- | --- | --- | --- | --- | --- | --- | --- |
| Rv2257c | NA | Hypothetical protein | NA | beta-lactamase | Homologue of transposon insertion site gene identified during a screen of M. smegmatis mutants showing beta-lactam hypersusceptibility. Beta-lactamase domain pfam00144, PRK10662, PRK11289. | Flores et al. 2005, J Bacteriol. 187(6): 1892-1900. blastx NCBI domain check. | H |
| Rv0050 | ponA1; pbp1 | Probable bifunctional penicillin-binding protein 1A/1B PonA1 (murein polymerase) (PBP1):penicillin-insensitive transglycosylase (peptidoglycan TGASE) + penicillin-sensitive transpeptidase) | Class A high molecular mass PBP. Contains a CCG microsatellite repeat - variable number of repeats. Belongs to the transglycosylase family in the N-terminal section,and to the transpeptidase family in the C-terminal section. | PBP | Shows beta-lactam binding - ampicillin and amoxycillin can acetylate this protein. | Bhakta and Basu. 2002. Biochem J. 361: 635-639; Qin et al. 2011. Evol Biol. 11: 247. | H |
| Rv0849 | NA | Probable conserved integral membrane transport protein | NA | NA | Knock-out mutant shows decresed beta-lactam MICs | Dinesh et al. 2013. Antimicrob Agents Chemother. 57(4):1941-1943 | H |
| Rv1218c | NA | Probable tetronasin-transport ATP-binding protein ABC transporter | NA | NA | Knock-out mutant shows decresed beta-lactam MICs | Dinesh et al. 2013. Antimicrob Agents Chemother. 57(4):1941-1943 | H |
| Rv1258c | NA | Probable conserved integral membrane transport protein | NA | NA | Knock-out mutant shows decresed beta-lactam MICs | Dinesh et al. 2013. Antimicrob Agents Chemother. 57(4):1941-1943 | H |
| Rv3065 | mmr | Multidrugs-transport integral membrane protein Mmr | NA | NA | Knock-out mutant shows decresed beta-lactam MICs | Dinesh et al. 2013. Antimicrob Agents Chemother. 57(4):1941-1943 | H |
| Rv0116c | ldtMt1; ldtA | Probable L,D-transpeptidase LdtA | NA | LDT | Bond and acetylated by several tested carbapenams and chephalosporins | DubÌ©e et el. Antimicrob Agents Chemother. 56(8): 4189-4195. | H |
| Rv2181 | NA | Alpha(1->2)mannosyltransferase | Affects lipoarabinomannan and lipomannan production which in turn are thought to play a role in cell wall integrity and restrictoin of pore sizes. | | Knockout and overexpression mutants showed increased sensitivity to meropenem. | Fukuda et al. 2013. mBio 4(1): e00472-12 | H |
| Rv2911 | dacB2 | Probable penicillin-binding protein DacB2 (D-alanyl-D-alanine carboxypeptidase) (DD-peptidase) (DD-carboxypeptidase) (PBP) (DD-transpeptidase) (serine-type D-ala-D-ala carboxypeptidase) (D-amino acid hydrolase) | Belongs to peptidase family S11; also known as the D-alanyl-D-alanine carboxypeptidase 1 family. Thought to be a membrane-bound protein. Note that previously known as dacB. | PBP | Evidence that meropenem inhibits D,d-carboxypeptidase activity PLUS meropenem shown to bind to this protein. | Kumar et al. 2012. Molec Microbiol. 86(2): 367-381. | H |
| Rv3330 | dacB1 | Probable penicillin-binding protein DacB1 (D-alanyl-D-alanine carboxypeptidase) (DD-peptidase) (DD-carboxypeptidase) (PBP) (DD-transpeptidase) (serine-type D-ala-D-ala carboxypeptidase) (D-amino acid hydrolase) | Belongs to peptidase family S11; also known as the D-alanyl-D-alanine carboxypeptidase 1 family. Conserved in M. tuberculosis, M. leprae, M. bovis and M. avium paratuberculosis; predicted to be essential for in vivo survival and pathogenicity (See Ribeiro-Guimaraes and Pessolani, 2007). | PBP | Evidence that meropenem inhibits D,d-carboxypeptidase activity. | Kumar et al. 2012. Molec Microbiol. 86(2): 367-381. | H |
| Rv2093c | tatC | Sec-independent protein translocase transmembrane protein TatC | NA | NA | BlaC exported through Tat pathway. In M. smegmatis tatC homologue mutaant reduced BlaS export and lead to reduction of carbenicillin MIC. | McDonough et al. 2005. J Bacteriol. 187(22):7667-7679 | H |
| Rv2094c | tatA | Sec-independent protein translocase membrane-bound protein TatA | NA | NA | BlaC exported through Tat pathway. In M. smegmatis tatA homologue mutaant reduced BlaS export and lead to reduction of carbenicillin MIC. | McDonough et al. 2005. J Bacteriol. 187(22):7667-7679 | H |
| Rv0406c | NA | Beta lactamase like protein | beta-lactamase activity | beta-lactamase | Shown to have beta-lactamase activity. | Nampoothiri et al. 2008. J Appl Microbiol. 105:59-67. | H |
| Rv3677c | NA | Possible hydrolase | beta-lactamase activity | beta-lactamase | Shown to have beta-lactamase activity. | Nampoothiri et al. 2008. J Appl Microbiol. 105:59-67. | H |
| Rv2525c | NA | Conserved hypothetical protein Secreted; predicted to be a substrate of the twin arginine translocation (tat) export system | NA | NA | Knockout mutants showed a 2-4 fold reduction in beta-lactam MICs. | Saint-Joanis et al. 2006. J Bacteriol. 188(18):6669-6679. | H |
| Rv1303 | NA | Hypothetical protein | Transmemebrane protein. A core mycobacterial gene; conserved in mycobacterial strains | | Transcripton regulated blaI. Amoxicillin leads to a change in transcription. | Sala et al. 2009. Molec Microbiol. 71(5): 1102-1116. | H |
| Rv1304 | atpB | Probable ATP synthase a chain AtpB (protein 6) | NA | NA | Transcripton regulated blaI. Amoxicillin leads to a change in transcription. | Sala et al. 2009. Molec Microbiol. 71(5): 1102-1116. | H |
| Rv1305 | atpE | Probable ATP synthase C chain AtpE (lipid-binding protein) (dicyclohexylcarbodiimide-binding protein) | NA | NA | Transcripton regulated blaI. Amoxicillin leads to a change in transcription. | Sala et al. 2009. Molec Microbiol. 71(5): 1102-1116. | H |
| Rv1306 | atpF | Probable ATP synthase B chain AtpF | NA | NA | Transcripton regulated blaI. Amoxicillin leads to a change in transcription. | Sala et al. 2009. Molec Microbiol. 71(5): 1102-1116. | H |
| Rv1307 | atpH | Probable ATP synthase delta chain AtpH | NA | NA | Transcripton regulated blaI. Amoxicillin leads to a change in transcription. | Sala et al. 2009. Molec Microbiol. 71(5): 1102-1116. | H |
| Rv1308 | atpA | Probable ATP synthase alpha chain AtpA | NA | NA | Transcripton regulated blaI. Amoxicillin leads to a change in transcription. | Sala et al. 2009. Molec Microbiol. 71(5): 1102-1116. | H |
| Rv1309 | atpG | Probable ATP synthase gamma chain AtpG | NA | NA | Transcripton regulated blaI. Amoxicillin leads to a change in transcription. | Sala et al. 2009. Molec Microbiol. 71(5): 1102-1116. | H |
| Rv1310 | atpD | Probable ATP synthase beta chain AtpD | NA | NA | Transcripton regulated blaI. Amoxicillin leads to a change in transcription. | Sala et al. 2009. Molec Microbiol. 71(5): 1102-1116. | H |
| Rv1311 | atpC | Probable ATP synthase epsilon chain AtpC | NA | NA | Transcripton regulated blaI. Amoxicillin leads to a change in transcription. | Sala et al. 2009. Molec Microbiol. 71(5): 1102-1116. | H |
| Rv1312 | NA | Conserved hypothetical secreted protein | Conserved hypothetical secreted protein with potential N-terminal signal sequence. N-terminus hydrophobic. | | Transcripton regulated blaI. Amoxicillin leads to a change in transcription. | Sala et al. 2009. Molec Microbiol. 71(5): 1102-1116. | H |
| Rv1456c | NA | Probable unidentified antibiotic-transport integral membrane ABC transporter | NA | NA | Transcripton regulated blaI. Amoxicillin leads to a change in transcription. | Sala et al. 2009. Molec Microbiol. 71(5): 1102-1116. | H |
| Rv1846c | blaI | Transcriptional repressor BlaI | NA | NA | BlaI represses expression of blaC beta-lactamase. Amoxicillin de-represses blaI trasncription. | Sala et al. 2009. Molec Microbiol. 71(5): 1102-1116. | H |
| Rv1847 | NA | Hypothetical protein | Possible thioesterase | NA | Transcripton regulated blaI. Amoxicillin leads to a change in transcription. | Sala et al. 2009. Molec Microbiol. 71(5): 1102-1116. | H |
| Rv2069 | sigC | RNA polymerase sigma factor, ECF subfamily, SigC | NA | NA | Transcripton regulated blaI. Amoxicillin leads to a change in transcription. | Sala et al. 2009. Molec Microbiol. 71(5): 1102-1116. | H |
| Rv3921c | NA | Probable conserved transmembrane protein | NA | NA | Transcripton regulated blaI. Amoxicillin leads to a change in transcription. | Sala et al. 2009. Molec Microbiol. 71(5): 1102-1116. Cohen paper | H |
| Rv2068c | blaC | Class A beta-lactamase BlaC | Contains PS00013 Prokaryotic lipid attachment site near N-terminus, and PS00146 Beta-lactamase class-a active site. | beta-lactamase | Transcripton regulated blaI. Amoxicillin leads to a change in transcription. Shown to have beta-lactamase activity. | Sala et al. 2009. Molec Microbiol. 71(5): 1102-1116. Nampoothiti et al. 2008. J Appl Microbiol. 105: 59-67. | H |
| Rv2163c | pbpB; pbp3; ftsI | Probable penicillin-binding membrane protein PbpB | NA | PBP/beta-lactamase | Inhibited by cefalexin and piperacillin. Also shown to bind penicillin, and interact with FtsW and FtsZ. Blastx NCBI transpeptidase domain pfam00905. | Slayden and Belisle. 2009. A Antimicrob Agents Chemother. 63:451-457. Datta et al. 2006. Molec Microbiol. 62(6):1655-1673. | H |
| Rv2752c | NA | Hypothetical protein | NA | beta-lactamase | Shown to have beta-lactamase activity. | Sun et al. 2011. Biochem (Moscow). 76(3):350-358. | H |
| Rv2373c | dnaJ2 | Probable chaperone protein DnaJ2 | NA | NA | Shown to interact with Rv2752c and inhibit its beta-lactamase activity. | Sun et al. 2011. Biochem (Moscow). 76(3):350-358. | H |
| Rv0867c | rpfA | Possible resuscitation-promoting factor RpfA | NA | NA | Knockout mutants showed increased susceptibility to beta-lactam bacteriocidal activity (up to 8 fold decrease in IC90 when all 5 rpf genes knocked out). | Wivagg and Hung 2011. Antimicrob Agents Chemother. 56(3): 1591-1594. | H |
| Rv1009 | rpfB | Probable resuscitation-promoting factor RpfB | NA | NA | Knockout mutants showed increased susceptibility to beta-lactam bacteriocidal activity (up to 8 fold decrease in IC90 when all 5 rpf genes knocked out). | Wivagg and Hung 2011. Antimicrob Agents Chemother. 56(3): 1591-1594. | H |
| Rv1884c | rpfC | Probable resuscitation-promoting factor RpfC | NA | NA | Knockout mutants showed increased susceptibility to beta-lactam bacteriocidal activity (up to 8 fold decrease in IC90 when all 5 rpf genes knocked out). | Wivagg and Hung 2011. Antimicrob Agents Chemother. 56(3): 1591-1594. | H |
| Rv2389c | rpfD | Probable resuscitation-promoting factor RpfD | NA | NA | Knockout mutants showed increased susceptibility to beta-lactam bacteriocidal activity (up to 8 fold decrease in IC90 when all 5 rpf genes knocked out). | Wivagg and Hung 2011. Antimicrob Agents Chemother. 56(3): 1591-1594. | H |
| Rv2450c | rpfE | Probable resuscitation-promoting factor RpfE | NA | NA | Knockout mutants showed increased susceptibility to beta-lactam bacteriocidal activity (up to 8 fold decrease in IC90 when all 5 rpf genes knocked out). | Wivagg and Hung 2011. Antimicrob Agents Chemother. 56(3): 1591-1594. | H |
| Rv1497 | lipL | B-lactamase/lipid metabolism | NA | NA | B-lactamase activity | Signh et al., 2010. Enzyme and microbial technology. doi:10.1016/j.enzmictec.2015.10.007 | H |
| Rv3681c | WhiB4 | Related to AG exposure | NA | NA | NA | Mishra et al., 2017. eLife | H |
| Rv1845c | blaR | Related to AG exposure | NA | NA | NA | Mishra et al., 2017. eLife | H |
| Rv0899 | ompA | Listed by Mishra | NA | NA | Porin. Slow poing for B-lactams | Mishra et al., 2017. eLife. Smani et al., 2014. Antimicrobial agents and chemotherapy | H |
| Rv0486 | MshA | Related to AG exposure | NA | NA | NA | Mishra et al., 2017. eLife | H |
| Rv1908c | KatG | Related to AG exposure. Synthetic lethality screen | NA | NA | Multifunctional enzyme, exhibiting both a catalase, a broad-spectrum peroxidase, and a peroxynitritase activities | Mishra et al., 2017. eLife. Lun et al. 2014. mBio | H |
| Rv3223c | SigH | Related to AG exposure | NA | NA | NA | Mishra et al., 2017. eLife | H |
| NA | crfA | Novel B-lactamase | NA | NA | NA | Kumar et al. 2017 Antimicrobial agents and chemotherapy | H |
| Rv1565c | MT1616 | Synthetic lethality screen | NA | synthetic lethality with pennicillin | Unknown. Further verification from authors | Lun et al. 2014. mBio | H |
| Rv0016c | pbpA | Synthetic lethality screen | NA | reduced growth upon exposure to penicillin, | Probable penicillin-binding protein PbpA | Lun et al. 2014. mBio | H |
| Rv0070c | glyA2 | Synthetic lethality screen | NA | involved in ë_-lactam susceptibility | Key enzyme in the biosynthesis of purines, lipids, other components. | Lun et al. 2014. mBio | H |
| Rv0320 | MT0335 | Synthetic lethality screen | NA | reduced growth upon exposure to penicillin, | NA | Lun et al. 2014. mBio | H |
| Rv1104 | MT1136 | Synthetic lethality screen | NA | reduced growth upon exposure to penicillin, | NA | Lun et al. 2014. mBio | H |
| Rv1965 | MT2017 | Synthetic lethality screen | NA | reduced growth upon exposure to penicillin, | NA | Lun et al. 2014. mBio | H |
| Rv1966 | MT2018 | Synthetic lethality screen | NA | reduced growth upon exposure to penicillin, | NA | Lun et al. 2014. mBio | H |
| Rv2224c | caeA; expA | Probable carboxylesterase CaeA. Synthetic lethality screen | NA | reduced growth upon exposure to penicillin, more susceptible to isoniazid and clarithromycin, in addition to imipenem | Homologue of transposon insertion site gene identified during a screen of M. smegmatis mutants showing beta-lactam hypersusceptibility. | Flores et al. 2005, J Bacteriol. 187(6): 1892-1900. Lun et al. 2014. mBio | H |
| MT2954 | MT2954 | Synthetic lethality screen | NA | reduced growth upon exposure to penicillin, | NA | Lun et al. 2014. mBio | H |
| Rv1625c | MT1661 | Synthetic lethality screen | NA | hyper-resistance to penicillin | NA | Lun et al. 2014. mBio | H |
| Rv3723 | MT3826 | Synthetic lethality screen | NA | hyper-resistance to penicillin | NA | Lun et al. 2014. mBio | H |
| Rv3214 | MT3310 | Synthetic lethality screen | NA | hyper-resistance to penicillin | NA | Lun et al. 2014. mBio | H |
| MT1227 | MT1227 | Synthetic lethality screen | NA | involved in ë_-lactam susceptibility | NA | Lun et al. 2014. mBio | H |
| Rv0948c | MT0975 | Synthetic lethality screen | NA | synthetic lethality with pennicillin | NA | Lun et al. 2014. mBio | H |
| Rv1024 | cdpAB | Possible conserved membrane protein | NA | NA | Homologue of transposon insertion site gene identified during a screen of M. smegmatis mutants showing beta-lactam hypersusceptibility. | Flores et al. 2005, J Bacteriol. 187(6): 1892-1900. | L |
| Rv1025 | cdpAB | hypothetical protein | NA | NA | Homologue of transposon insertion site gene identified during a screen of M. smegmatis mutants showing beta-lactam hypersusceptibility. | Flores et al. 2005, J Bacteriol. 187(6): 1892-1900. | L |
| Rv2198c | mmpS3 | Probable conserved membrane protein MmpS3 | NA | NA | Identified by transposon mutanat screen of beta-lactam hypersusceptible Mtb. | Flores et al. 2005, J Bacteriol. 187(6): 1892-1900. | L |
| Rv2223c | expA | Probable exported protease | NA | NA | Homologue of transposon insertion site gene identified during a screen of M. smegmatis mutants showing beta-lactam hypersusceptibility. | Flores et al. 2005, J Bacteriol. 187(6): 1892-1900. | L |
| Rv2256c | ump | hypothetical protein | NA | NA | Homologue of transposon insertion site gene identified during a screen of M. smegmatis mutants showing beta-lactam hypersusceptibility. | Flores et al. 2005, J Bacteriol. 187(6): 1892-1900. | L |
| Rv2258c | mtxA | Possible transcriptional regulatory protein | NA | NA | Homologue of transposon insertion site gene identified during a screen of M. smegmatis mutants showing beta-lactam hypersusceptibility. | Flores et al. 2005, J Bacteriol. 187(6): 1892-1900. | L |
| Rv2509 | sdrA | Probable short-chain type dehydrogenase/reductase | NA | NA | Homologue of transposon insertion site gene identified during a screen of M. smegmatis mutants showing beta-lactam hypersusceptibility. | Flores et al. 2005, J Bacteriol. 187(6): 1892-1900. | L |
| Rv2773c | dapB | Dihydrodipicolinate reductase DapB (DHPR) | NA | NA | Homologue of transposon insertion site gene identified during a screen of M. smegmatis mutants showing beta-lactam hypersusceptibility. | Flores et al. 2005, J Bacteriol. 187(6): 1892-1900. | L |
| Rv2844 | NA | Conserved alanine rich protein | NA | NA | Identified by transposon mutanat screen of beta-lactam hypersusceptible Mtb. | Flores et al. 2005, J Bacteriol. 187(6): 1892-1900. | L |
| Rv2926c | nabA | hypothetical protein | NA | NA | Homologue of transposon insertion site gene identified during a screen of M. smegmatis mutants showing beta-lactam hypersusceptibility. | Flores et al. 2005, J Bacteriol. 187(6): 1892-1900. | L |
| Rv2927c | cdpC | hypothetical protein | NA | NA | Homologue of transposon insertion site gene identified during a screen of M. smegmatis mutants showing beta-lactam hypersusceptibility. | Flores et al. 2005, J Bacteriol. 187(6): 1892-1900. | L |
| Rv0014c | pknB | Transmembrane serine/threonine-protein kinase B PknB (protein kinase B) (STPK B) | NA | NA | Contains PASTA domain. Shown to interact with MurC -D, -E, -F | Cole et al. 1998. Nature 393: 597-544. Munshi et al 2013. PLoS One e60143. | L |
| Rv2107 | PE22 | PE family protein PE22 | NA | PE | Homologue of transposon insertion site gene identified during a screen of M. bovis mutants with amplicillin MICs greater than that of the wt strain. | Danilchanka et al. 2008 Antimicrob Agents Chemother 52(7):2503-2511 | L |
| Rv0755c | PPE12 | PPE family protein PPE12 | NA | PPE | Homologue of transposon insertion site gene identified during a screen of M. bovis mutants with amplicillin MICs greater than that of the wt strain. | Danilchanka et al. 2008 Antimicrob Agents Chemother 52(7):2503-2511 | L |
| Rv1753c | PPE24 | PPE family protein PPE24 | NA | PPE | Homologue of transposon insertion site gene identified during a screen of M. bovis mutants with amplicillin MICs greater than that of the wt strain. | Danilchanka et al. 2008 Antimicrob Agents Chemother 52(7):2503-2511 | L |
| Rv3159c | PPE53 | PPE family protein PPE53 | NA | PPE | Homologue of transposon insertion site gene identified during a screen of M. bovis mutants with amplicillin MICs greater than that of the wt strain. | Danilchanka et al. 2008 Antimicrob Agents Chemother 52(7):2503-2511 | L |
| Rv0030 | NA | hypothetical protein | NA | NA | Homologue of transposon insertion site gene identified during a screen of M. bovis mutants with amplicillin MICs greater than that of the wt strain. | Danilchanka et al. 2008 Antimicrob Agents Chemother 52(7):2503-2511 | L |
| Rv0112 | gca | Possible GDP-mannose 4,6-dehydratase Gca (GDP-D-mannose dehydratase) | NA | NA | Homologue of transposon insertion site gene identified during a screen of M. bovis mutants with amplicillin MICs greater than that of the wt strain. | Danilchanka et al. 2008 Antimicrob Agents Chemother 52(7):2503-2511 | L |
| Rv0113 | gmhA | Probable sedoheptulose-7-phosphate isomerase GmhA (phosphoheptose isomerase) | NA | NA | Homologue of transposon insertion site gene identified during a screen of M. bovis mutants with amplicillin MICs greater than that of the wt strain. | Danilchanka et al. 2008 Antimicrob Agents Chemother 52(7):2503-2511 | L |
| Rv0194 | NA | Probable transmembrane multidrug efflux pump | NA | NA | Homologue of transposon insertion site gene identified during a screen of M. bovis mutants with amplicillin MICs greater than that of the wt strain. | Danilchanka et al. 2008 Antimicrob Agents Chemother 52(7):2503-2511 | L |
| Rv0806c | cpsY | Possible UDP-glucose-4-epimerase CpsY (galactowaldenase) (UDP-galactose-4-epimerase) (uridine diphosphate galactose-4-epimerase) (uridine diphospho-galactose-4-epimerase) | NA | NA | Homologue of transposon insertion site gene identified during a screen of M. bovis mutants with amplicillin MICs greater than that of the wt strain. | Danilchanka et al. 2008 Antimicrob Agents Chemother 52(7):2503-2511 | L |
| Rv1503c | NA | hypothetical protein | NA | NA | Homologue of transposon insertion site gene identified during a screen of M. bovis mutants with amplicillin MICs greater than that of the wt strain. | Danilchanka et al. 2008 Antimicrob Agents Chemother 52(7):2503-2511 | L |
| Rv1521 | fadD25 | Probable fatty-acid-AMP ligase FadD25 (fatty-acid-AMP synthetase) (fatty-acid-AMP synthase) | NA | NA | Homologue of transposon insertion site gene identified during a screen of M. bovis mutants with amplicillin MICs greater than that of the wt strain. | Danilchanka et al. 2008 Antimicrob Agents Chemother 52(7):2503-2511 | L |
| Rv1522c | mmpL12 | Probable conserved transmembrane transport protein MmpL12 | NA | NA | Homologue of transposon insertion site gene identified during a screen of M. bovis mutants with amplicillin MICs greater than that of the wt strain. | Danilchanka et al. 2008 Antimicrob Agents Chemother 52(7):2503-2511 | L |
| Rv1665 | pks11 | Chalcone synthase Pks11 | NA | NA | Homologue of transposon insertion site gene identified during a screen of M. bovis mutants with amplicillin MICs greater than that of the wt strain. | Danilchanka et al. 2008 Antimicrob Agents Chemother 52(7):2503-2511 | L |
| Rv1810 | NA | hypothetical protein | NA | NA | Homologue of transposon insertion site gene identified during a screen of M. bovis mutants with amplicillin MICs greater than that of the wt strain. | Danilchanka et al. 2008 Antimicrob Agents Chemother 52(7):2503-2511 | L |
| Rv1949c | NA | hypothetical protein | NA | NA | Homologue of transposon insertion site gene identified during a screen of M. bovis mutants with amplicillin MICs greater than that of the wt strain. | Danilchanka et al. 2008 Antimicrob Agents Chemother 52(7):2503-2511 | L |
| Rv2104c | vapB37 | Possible antitoxin VapB37 | NA | NA | Homologue of transposon insertion site gene identified during a screen of M. bovis mutants with amplicillin MICs greater than that of the wt strain. | Danilchanka et al. 2008 Antimicrob Agents Chemother 52(7):2503-2511 | L |
| Rv2276 | cyp121 | Cytochrome P450 121 Cyp121 | NA | NA | Homologue of transposon insertion site gene identified during a screen of M. bovis mutants with amplicillin MICs greater than that of the wt strain. | Danilchanka et al. 2008 Antimicrob Agents Chemother 52(7):2503-2511 | L |
| Rv2307B | NA | Hypothetical glycine rich protein | NA | NA | Homologue of transposon insertion site gene identified during a screen of M. bovis mutants with amplicillin MICs greater than that of the wt strain. | Danilchanka et al. 2008 Antimicrob Agents Chemother 52(7):2503-2511 | L |
| Rv2543 | lppA | Probable conserved lipoprotein LppA | NA | NA | Homologue of transposon insertion site gene identified during a screen of M. bovis mutants with amplicillin MICs greater than that of the wt strain. | Danilchanka et al. 2008 Antimicrob Agents Chemother 52(7):2503-2511 | L |
| Rv2544 | lppB | Probable conserved lipoprotein LppB | NA | NA | Homologue of transposon insertion site gene identified during a screen of M. bovis mutants with amplicillin MICs greater than that of the wt strain. | Danilchanka et al. 2008 Antimicrob Agents Chemother 52(7):2503-2511 | L |
| Rv2721c | NA | Possible conserved transmembrane alanine and glycine rich protein | NA | NA | Homologue of transposon insertion site gene identified during a screen of M. bovis mutants with amplicillin MICs greater than that of the wt strain. | Danilchanka et al. 2008 Antimicrob Agents Chemother 52(7):2503-2511 | L |
| Rv2722 | NA | hypothetical protein | NA | NA | Homologue of transposon insertion site gene identified during a screen of M. bovis mutants with amplicillin MICs greater than that of the wt strain. | Danilchanka et al. 2008 Antimicrob Agents Chemother 52(7):2503-2511 | L |
| Rv2730 | NA | Hypothetical protein | NA | NA | Homologue of transposon insertion site gene identified during a screen of M. bovis mutants with amplicillin MICs greater than that of the wt strain. | Danilchanka et al. 2008 Antimicrob Agents Chemother 52(7):2503-2511 | L |
| Rv2806 | NA | Possible membrane protein | NA | NA | Homologue of transposon insertion site gene identified during a screen of M. bovis mutants with amplicillin MICs greater than that of the wt strain. | Danilchanka et al. 2008 Antimicrob Agents Chemother 52(7):2503-2511 | L |
| Rv2809 | NA | Hypothetical protein | NA | NA | Homologue of transposon insertion site gene identified during a screen of M. bovis mutants with amplicillin MICs greater than that of the wt strain. | Danilchanka et al. 2008 Antimicrob Agents Chemother 52(7):2503-2511 | L |
| Rv2943 | NA | Probable transposase for insertion sequence element IS1533 | NA | NA | Homologue of transposon insertion site gene identified during a screen of M. bovis mutants with amplicillin MICs greater than that of the wt strain. | Danilchanka et al. 2008 Antimicrob Agents Chemother 52(7):2503-2511 | L |
| Rv3107c | agpS | Possible alkyldihydroxyacetonephosphate synthase AgpS (alkyl-DHAP synthase) (alkylglycerone-phosphate synthase) | NA | NA | Homologue of transposon insertion site gene identified during a screen of M. bovis mutants with amplicillin MICs greater than that of the wt strain. | Danilchanka et al. 2008 Antimicrob Agents Chemother 52(7):2503-2511 | L |
| Rv3124 | moaR1 | Transcriptional regulatory protein MoaR1 | NA | NA | Homologue of transposon insertion site gene identified during a screen of M. bovis mutants with amplicillin MICs greater than that of the wt strain. | Danilchanka et al. 2008 Antimicrob Agents Chemother 52(7):2503-2511 | L |
| Rv3635 | NA | Probable conserved transmembrane protein | NA | NA | Homologue of transposon insertion site gene identified during a screen of M. bovis mutants with amplicillin MICs greater than that of the wt strain. | Danilchanka et al. 2008 Antimicrob Agents Chemother 52(7):2503-2511 | L |
| Rv3727 | NA | Possible oxidoreductase | NA | NA | Homologue of transposon insertion site gene identified during a screen of M. bovis mutants with amplicillin MICs greater than that of the wt strain. | Danilchanka et al. 2008 Antimicrob Agents Chemother 52(7):2503-2511 | L |
| Rv3820c | papA2 | Possible conserved polyketide synthase associated protein PapA2 | NA | NA | Homologue of transposon insertion site gene identified during a screen of M. bovis mutants with amplicillin MICs greater than that of the wt strain. | Danilchanka et al. 2008 Antimicrob Agents Chemother 52(7):2503-2511 | L |
| Rv3823c | mmpL8 | Conserved integral membrane transport protein MmpL8 | NA | NA | Homologue of transposon insertion site gene identified during a screen of M. bovis mutants with amplicillin MICs greater than that of the wt strain. | Danilchanka et al. 2008 Antimicrob Agents Chemother 52(7):2503-2511 | L |
| Rv3826 | fadD23 | Probable fatty-acid-AMP ligase FadD23 (fatty-acid-AMP synthetase) (fatty-acid-AMP synthase) | NA | NA | Homologue of transposon insertion site gene identified during a screen of M. bovis mutants with amplicillin MICs greater than that of the wt strain. | Danilchanka et al. 2008 Antimicrob Agents Chemother 52(7):2503-2511 | L |
| Rv3903c | NA | Hypothetical alanine and proline rich protein | NA | NA | Homologue of transposon insertion site gene identified during a screen of M. bovis mutants with amplicillin MICs greater than that of the wt strain. | Danilchanka et al. 2008 Antimicrob Agents Chemother 52(7):2503-2511 | L |
| Rv3682 | ponA2; MT3784 | Probable bifunctional membrane-associated penicillin-binding protein 1A/1B PonA2 (murein polymerase) [includes: penicillin-insensitive transglycosylase (peptidoglycan TGASE) + penicillin-sensitive transpeptidase (DD-transpeptidase)]. Synthetic lethality screen | NA | PBP | Identified by transposon mutant screen, led to 97 % raw and 65 % adjusted growth reduction. Plus idenified by transposon mutant screen of beta-lactam hypersusceptible mutants (Mtb and M. segmentis). Contains PASTA domain. Involved in peptidoglycan synthesis (at the final stages), cell wall formation. | Keira, transposon mutant screen data - mutants susceptible to Imipenem; Flores et al. 2005, J Bacteriol. 187(6): 1892-1900. Cole et al. 1998. Nature 393: 597-544. Lun et al. 2014. mBio | H |
| Rv2518c | ldtMt2; ldtB; lppS; MT2594 | Probable L,D-transpeptidase LdtB. Synthetic lethality screen | Has very long signal sequence and appropriately positioned PS00013 Prokaryotic membrane lipoprotein lipid attachment site. | LDT. Probable L,D-transpeptidase LdtB | Identified by transposon mutant screen, led to 91.4559721011334 % raw and 97.3717418126648 % adjusted growth reduction. AND a second mutant with 98.2380686717046 % raw and 101.730288495621 % adjusted growth reduction. Also identified by transposon mutant screen for growth deftects - the same mutant showed increased susceptibility to Amoxicillin-clavulanate. | Keira, transposon mutant screen data - mutants susceptible to Imipenem; Gupta et al. 2010. Nat Med 16(4): 466-470. Lun et al. 2014. mBio | H |
| Rv0192 | NA | Hypothetical protein | NA | LDT | L, D transpeptidase domain cd13431, cd13432, cd13430. | Keira's list - NCBI blastx confirm domain presence. | L |
| Rv1433 | NA | Possible conserved exported protein | L,D transpeptidase catalytic domain | LDT | L, D transpeptidase domain cd13431, cd13432, cd13430. | Keira's list - NCBI blastx confirm domain presence. | L |
| Rv3627c | NA | Hypothetical protein | D,D peptidase | PBP | D, D carboxypeptidase domain pfam02113, DacB (pbp4) domain COG2027, D,D carboxypeptidase domain PRK11113. | Keira's list - NCBI blastx confirm domain presence. | L |
| Rv0015c | pknA | Transmembrane serine/threonine-protein kinase A PknA (protein kinase A) (STPK A) | NA | NA | Shown to interact with MurC, -D, -E and/or -F. | Munshi et al 2013. PLoS One e60143. | L |
| Rv1338 | murI | Probable glutamate racemase MurI | NA | NA | Shown to interact with MurC, -D, -E and/or -F. | Munshi et al 2013. PLoS One e60143. | L |
| Rv2145c | wag31 | Diviva family protein Wag31 | NA | NA | Shown to interact with MurC, -D, -E and/or -F. | Munshi et al 2013. PLoS One e60143. | L |
| Rv2147c | CDS | hypothetical protein | NA | NA | Shown to interact with MurC, -D, -E and/or -F. | Munshi et al 2013. PLoS One e60143. | L |
| Rv2151c | ftsQ | Possible cell division protein FtsQ | NA | NA | Shown to interact with MurC, -D, -E and/or -F. | Munshi et al 2013. PLoS One e60143. | L |
| Rv2152c | murC | Probable UDP-N-acetylmuramate-alanine ligase MurC | NA | NA | Initiates formation of stem peptides during peptidoglycan formation. | Munshi et al 2013. PLoS One e60143. | L |
| Rv2155c | murD | Probable UDP-N-acetylmuramoylalanine-D-glutamate ligase MurD | NA | NA | Involved in formation of stem peptides during peptidoglycan formation. | Munshi et al 2013. PLoS One e60143. | L |
| Rv2156c | murX | Probable phospho-N-acetylmuramoyl-pentapeptidetransferase MurX | NA | NA | Shown to interact with MurC, -D, -E and/or -F. | Munshi et al 2013. PLoS One e60143. | L |
| Rv2157c | murF | Probable UDP-N-acetylmuramoylalanyl-D-glutamyl-2, 6-diaminopimelate-D-alanyl-D-alanyl ligase MurF | NA | NA | Involved in formation of stem peptides during peptidoglycan formation. | Munshi et al 2013. PLoS One e60143. | L |
| Rv2158c | murE | Probable UDP-N-acetylmuramoylalanyl-D-glutamate-2,6-diaminopimelate ligase MurE | NA | NA | Involved in formation of stem peptides during peptidoglycan formation. | Munshi et al 2013. PLoS One e60143. | L |
| Rv2160c | CDS | hypothetical protein | NA | NA | Shown to interact with MurC, -D, -E and/or -F. | Munshi et al 2013. PLoS One e60143. | L |
| Rv2981c | ddlA | Probable D-alanine--D-alanine ligase DdlA (D-alanylalanine synthetase) (D-ala-D-ala ligase) | NA | NA | Shown to interact with MurC, -D, -E and/or -F. | Munshi et al 2013. PLoS One e60143. | L |
| Rv3818 | namH | NamH, muramic acid glycosylase. | NA | NA | Shown to interact with MurC, -D, -E and/or -F. | Munshi et al 2013. PLoS One e60143. | L |
| Rv2150c | ftsZ | Cell division protein FtsZ | NA | NA | Shown to interact with MurC, -D, -E and/or -F. Also shown to interact with FtsW and PBP3. | Munshi et al 2013. PLoS One e60143. Datta et al. 2006. Molec Microbiol. 62(6):1655-1673. | L |
| Rv2726c | dapF | Probable diaminopimelate epimerase DapF (DAP epimerase) | NA | NA | Shown to interact with MurC, -D, -E and/or -F. Known to be DAP epimerase respinsible for interconversion of peptidoglycan cross-links. | Munshi et al 2013. PLoS One e60143. Usha et al. 2009. Acta Crystallographica Section D 65:383-387. | L |
| Rv2154c | ftsW | FtsW-like protein FtsW | NA | NA | Shown to interact with MurC, -D, -E and/or -F. Also shown to interact with FtsZ and PBP3. | Munshi et al 2013. PLoS One e60143. Datta et al. 2006. Molec Microbiol. 62(6):1655-1673. | L |
| Rv0011c | crgA | Probable conserved transmembrane protein | NA | NA | Co-localises with pbpA and pbpB in M. smegmatis. crgA knowckouts show altered pbp localisation. | Plocinski et al. 2011. J Bacteriol. 193(13): 3246-3256. | L |
| Rv2864c | NA | Possible penicillin-binding lipoprotein | NA | PBP | Annotated as possible PBP in Genbank - based on protein similarity. Blastx NCBI transpeptidase binding domain pfam00905. | | L |
| Rv1730c | NA | Possible penicillin-binding protein | NA | PBP/beta-lactamase | Annotated as possible PBP in Genbank - based on protein similarity. |  | L |
| Rv2933 | ppsC | Synthetic lethality screen | NA | NA | Involved in phenolpthiocerol and phthiocerol dimycocerosate (dim) biosynthesis | Lun et al. 2014. mBio | L |
| Rv1248c | NA | Synthetic lethality screen | NA | NA | Involved in cellular metabolism. Has alpha-ketoglutarate dehydrogenase (KDH) | Lun et al. 2014. mBio | L |
| MT3042 | NA | Synthetic lethality screen | NA | NA | NA | Lun et al. 2014. mBio | L |
| Rv1143 | mcr | Synthetic lethality screen | NA | NA | Required for bile acid synthesis and for catabolism of branched-chain fatty acids | Lun et al. 2014. mBio | L |
| Rv1879 | NA | Synthetic lethality screen | NA | NA | Conserved hypothetical protein | Lun et al. 2014. mBio | L |
| Rv1957 | NA | Synthetic lethality screen | NA | NA | Hypothetical protein | Lun et al. 2014. mBio | L |
| Rv0404 | fadD30 | Synthetic lethality screen | NA | NA | involved in lipid degradation. | Lun et al. 2014. mBio | L |
| Rv2936 | drrA | Synthetic lethality screen | NA | NA | involved in active transport of antibiotic and phthiocerol dimycocerosate (dim) across the membrane (export) | Lun et al. 2014. mBio | L |
| Rv1348 | irtA | Synthetic lethality screen | NA | NA | Involved in iron homeostasis. | Lun et al. 2014. mBio | L |
| Rv1640c | lysX | Synthetic lethality screen | NA | NA | Charging LYS tRNA | Lun et al. 2014. mBio | L |
| Rv1319c | NA | Synthetic lethality screen | NA | NA | Possible adenylate cyclase | Lun et al. 2014. mBio | L |
| Rv0191 | NA | Synthetic lethality screen | NA | NA | possibly involved in transport of drug across the membrane | Lun et al. 2014. mBio | L |
| Rv1473A | NA | Synthetic lethality screen | NA | NA | Possible transcriptional regulatory protein | Lun et al. 2014. mBio | L |
| Rv1618 | tesB1 | Synthetic lethality screen | NA | NA | Involved in fatty acid metabolism. | Lun et al. 2014. mBio | L |
| Rv0324 | NA | Synthetic lethality screen | NA | NA | Possible transcriptional regulatory protein (possibly ArsR-family) | Lun et al. 2014. mBio | L |
| Rv1496 | NA | Synthetic lethality screen | NA | NA | Possibly involved in transport (possibly arginine) | Lun et al. 2014. mBio | L |
| Rv3410c | guaB3 | Synthetic lethality screen | NA | NA | Catalyses the first reaction unique to GMP biosynthesis | Lun et al. 2014. mBio | L |
| Rv1890c | NA | Synthetic lethality screen | NA | NA | Hypothetical protein | Lun et al. 2014. mBio | L |
| Rv1021 | NA | Synthetic lethality screen | NA | NA | Function unknown | Lun et al. 2014. mBio | L |
| Rv2115c | map | Synthetic lethality screen | NA | NA | Involved in proteasomal protein degradation | Lun et al. 2014. mBio | L |
| Rv2948c | fadD22 | Synthetic lethality screen | NA | NA | Involved in biosynthesis of phenolic glycolipids | Lun et al. 2014. mBio | L |
| Rv3290c | lat | Synthetic lethality screen | NA | NA | Possibly involved in L-alpha-aminoadipic acid (L-AAA) biosynthesis | Lun et al. 2014. mBio | L |
| Rv3485c | NA | Synthetic lethality screen | NA | NA | Probable short-chain type dehydrogenase/reductase | Lun et al. 2014. mBio | L |
| MT2960 | NA | Synthetic lethality screen | NA | NA | NA | Lun et al. 2014. mBio | L |
| Rv1819c | bacA | Synthetic lethality screen | NA | NA | Probable drug-transport transmembrane ATP-binding protein ABC transporter BacA | Lun et al. 2014. mBio | L |
| Rv3619c | esxV | Synthetic lethality screen | NA | NA | Unknown | Lun et al. 2014. mBio | L |
| Rv0491 | regX3 | Synthetic lethality screen | NA | NA | Transcriptional regulatory protein part of the two component regulatory system REGX3/SENX3 | Lun et al. 2014. mBio | L |
| Rv0467 | icl1 | Synthetic lethality screen | NA | NA | Involved in glyoxylate bypass (at the first step), an alternative to the tricarboxylic acid cycle (in bacteria, plants, and fungi) [catalytic activity: isocitrate = succinate + glyoxylate]. Involved in the persistence in the host. | Lun et al. 2014. mBio | L |
| Rv3506 | fadD17 | Synthetic lethality screen | NA | NA | Function unknown, but supposed involvement in lipid degradation. | Lun et al. 2014. mBio | L |
| Rv3864 | espE | Synthetic lethality screen | NA | NA | Function unknown | Lun et al. 2014. mBio | L |
| Rv2950c | fadD29 | Synthetic lethality screen | NA | NA | Involved in biosynthesis of phenolic glycolipids | Lun et al. 2014. mBio | L |
| Rv1358 | NA | Synthetic lethality screen | NA | NA | Probable transcriptional regulatory protein | Lun et al. 2014. mBio | L |
| Rv3486 | NA | Synthetic lethality screen | NA | NA | Function unknown | Lun et al. 2014. mBio | L |
| Rv0483 | lprQ | Synthetic lethality screen | NA | NA | Probable conserved lipoprotein LprQ | Lun et al. 2014. mBio | L |
| Rv2585c | NA | Synthetic lethality screen | NA | NA | Unknown | Lun et al. 2014. mBio | L |
| Rv3000 | NA | Synthetic lethality screen | NA | NA | Unknown | Lun et al. 2014. mBio | L |
| Rv0110 | NA | Synthetic lethality screen | NA | NA | Unknown | Lun et al. 2014. mBio | L |
| Rv0056 | rplI | Synthetic lethality screen | NA | NA | Binds to the 23S rRNA | Lun et al. 2014. mBio | L |
| MT3453 | NA | Synthetic lethality screen | NA | NA | NA | Lun et al. 2014. mBio | L |
| Rv2241 | aceE | Synthetic lethality screen | NA | NA | Involved in energy metabolism; contributes to acetyl-CoA production as part of pyruvate dehydrogenase complex | Lun et al. 2014. mBio | L |
| Rv0771 | NA | Synthetic lethality screen | NA | NA | Involved in aromatic hydrocarbons catabolism | Lun et al. 2014. mBio | L |
| Rv2067c | NA | Synthetic lethality screen | NA | NA | Unknown | Lun et al. 2014. mBio | L |
| Rv1263 | amiB2 | Synthetic lethality screen | NA | NA | Involved in cellular metabolism, active on 2- to 6- carbon aliphatic amides and on many aromatic amides | Lun et al. 2014. mBio | L |
| Rv1450c | PE_PGRS27 | Synthetic lethality screen | NA | NA | PE-PGRS family protein PE_PGRS27 | Lun et al. 2014. mBio | L |
| Rv2114 | NA | Synthetic lethality screen | NA | NA | Unknown | Lun et al. 2014. mBio | L |
| Rv2922A | acyP | Synthetic lethality screen | NA | NA | Involved in cellular metabolism | Lun et al. 2014. mBio | L |
| Rv0488 | NA | Synthetic lethality screen | NA | NA | possibly involved in transport of lysine across the membrane. | Lun et al. 2014. mBio | L |
| MT1071 | NA | Synthetic lethality screen | NA | NA | NA | Lun et al. 2014. mBio | L |
| Rv1638 | uvrA | Synthetic lethality screen | NA | NA | Involved in nucleotide excision repair. | Lun et al. 2014. mBio | L |
| Rv3210c | NA | Synthetic lethality screen | NA | NA | Function unknown | Lun et al. 2014. mBio | L |
| Rv2724c | fadE20 | Synthetic lethality screen | NA | NA | Function unknown, but involved in lipid degradation. | Lun et al. 2014. mBio | L |
| Rv3863 | NA | Synthetic lethality screen | NA | NA | Unknown alanine rich protein | Lun et al. 2014. mBio | L |
| Rv1245c | NA | Synthetic lethality screen | NA | NA | Probable short-chain type dehydrogenase/reductase | Lun et al. 2014. mBio | L |
| MT1849.1 | NA | Synthetic lethality screen | NA | NA | NA | Lun et al. 2014. mBio | L |
| Rv1190 | NA | Synthetic lethality screen | NA | NA | Function unknown | Lun et al. 2014. mBio | L |
| Rv3882c | eccE1 | Synthetic lethality screen | NA | NA | ESX conserved component EccE1. ESX-1 type VII secretion system protein. Possible membrane protein. | Lun et al. 2014. mBio | L |
| Rv1651c | PE_PGRS30 | Synthetic lethality screen | NA | NA | Function unknown. Thought to be involved in virulence. | Lun et al. 2014. mBio | L |
| Rv0188 | NA | Synthetic lethality screen | NA | NA | Probable conserved transmembrane protein | Lun et al. 2014. mBio | L |
| Rv0826 | NA | Synthetic lethality screen | NA | NA | Function unknown | Lun et al. 2014. mBio | L |
| Rv0214 | fadD4 | Synthetic lethality screen | NA | NA | Function unknown, but involved in lipid degradation. | Lun et al. 2014. mBio | L |
| Rv1226c | NA | Synthetic lethality screen | NA | NA | Unknown | Lun et al. 2014. mBio | L |
| Rv1838c | vapC13 | Synthetic lethality screen | NA | NA | Possible toxin VapC13 | Lun et al. 2014. mBio | L |
| Rv0443 | NA | Synthetic lethality screen | NA | NA | Function unknown | Lun et al. 2014. mBio | L |
| Rv3295 | NA | Synthetic lethality screen | NA | NA | Probable transcriptional regulatory protein (probably TetR-family) | Lun et al. 2014. mBio | L |
| Rv3566A | NA | Synthetic lethality screen | NA | NA | Unknown | Lun et al. 2014. mBio | L |
| Rv1747 | NA | Synthetic lethality screen | NA | NA | Probable conserved transmembrane ATP-binding protein ABC transporter | Lun et al. 2014. mBio | L |
| Rv1344 | mbtL | Synthetic lethality screen | NA | NA | Thought to be involved in the biogenesis of the hydroxyphenyloxazoline-containing siderophore mycobactins | Lun et al. 2014. mBio | L |
| Rv0232 | NA | Synthetic lethality screen | NA | NA | Probable transcriptional regulatory protein (probably TetR/AcrR-family) | Lun et al. 2014. mBio | L |
