# Supplementary material for: Collateral Sensitivity to β-Lactam Drugs in Drug-Resistant Tuberculosis Is Driven by the Transcriptional Wiring of BlaI Operon Genes
Source: mSphere. 2021 May 28;6(3):e00245-21. doi: 10.1128/mSphere.00245-21 (PMC8265638; doi:10.1128/mSphere.00245-21)
Supplement: TABLE S2 [file msphere.00245-21-st002.docx]

**Supplementary Table S2.**

| **Drug** | **Genes involved in resistance** |
| --- | --- |
| AMI (aminoglycosides) | *Rv1694, MTB000019* |
| EMB (ethambutol) | *Rv3795, Rv1267c, Rv3124, Rv3125c,* |
|  | *Rv3126, Rv0340, Rv0341, Rv0342,* |
|  | *Rv0343, Rv3264c, Rv3266c, Rv3793, Rv3794* |
| ETH (ehthionamide) | *Rv1484, Rv3854c, Rv1483* |
| FLQ (fluoroquinolones) | *Rv0006, Rv0005* |
| INH (isoniazid) | *Rv1908c, Rv2245, Rv1854c, Rv2427A, Rv2428, Rv1483, Rv1484, Rv3795, Rv1909c, Rv0340, Rv0341, Rv0342, Rv0343, Rv1592c,* |
|  | *Rv1772, Rv2242, Rv2243, Rv2247, Rv0129c, Rv3139, Rv2846c, Rv3566c* |
| PAS (para-aminosalisylic acid) | *Rv2764c* |
| PZA (pyrazinamide) | *Rv2043c* |
| RIF (rifampicin) | Rv0667, Rv3795 |
| SM (streptomycin) | MTB000019, Rv3919c, Rv0682 |
| SM, AMI | MTB000019 |
| EMB, INH | Rv0340, Rv0341, Rv0342, Rv0343 |
| ETH, INH | Rv1483, Rv1484 |
| EMB, INH, RIF | Rv3795 |
