# Supplementary material for: Collateral Sensitivity to β-Lactam Drugs in Drug-Resistant Tuberculosis Is Driven by the Transcriptional Wiring of BlaI Operon Genes
Source: mSphere. 2021 May 28;6(3):e00245-21. doi: 10.1128/mSphere.00245-21 (PMC8265638; doi:10.1128/mSphere.00245-21)
Supplement: TABLE S6 [file msphere.00245-21-st006.docx]

**Supplementary Table S6.**

| **Section** | **High confidence genes** | **All genes** |
| --- | --- | --- |
| ***Treatment with classical TB drugs induces the expression of β-lactamase inhibitors*** |  | |
| Variability of β-lactam^S^ vs non-β-lactam^S^ genes when affected by classical TB drugs (KS test p-value) | 0.027 | 0.012 |
| Variability of β-lactam^S^ vs non-β-lactam^S^ genes when affected by classical TB drugs (Wilcoxon test p-value) | 0.014 | 0.0029 |
| Variability of β-lactam^S^ vs non-β-lactam^S^ genes when affected by classical TB drugs (Permutation p-value) | 0.023 | 0.012 |
| % of genes significantly upregulated after drug treatment (Fisher exact test p-value) | 18.52% (0.024) | 14.47% (0.014) |
| ***Co-expression between β-lactam^S^ and DR genes*** |  |  |
| % of clusters containing two β-lactam^S^ genes that also contained DR genes (p-value) | 50% (p = 0.12) | 34.8% (p=0.054) |
| # of DR genes more correlated with TB drug genes than all other genes (%) | 30 of 37 (81.1%) | 31 of 37 (83.8%) |
| ***β-lactamS and DR nodes are highly linked in the molecular network of Mtb*** |  | |
| # of β-lactam^S^ nodes highly localized in a highly specific network region | 26 of 63 (41.2%) | 119 of 199 (59.8%) |
| Significance of β-lactam^S^ nodes interacting | 1x10^-35^ | 1x10^-28^ |
| Significance of cross-talk between β-lactam^S^ nodes and DR nodes (hypergeometric test q-value) | 0.00008 | 0.0002 |
| Significance of cross-talk between β-lactam^S^ nodes and RIF (hypergeometric test q-value) | 0.0003 | 0.0008 |
| Significance of cross-talk between β-lactam^S^ nodes and SM (hypergeometric test q-value) | 0.003 | 0.007 |
| Significance of cross-talk between β-lactam^S^ nodes and PAS (hypergeometric test q-value) | - | 0.001 |
| ***In silico functional validation of a dependence mechanism between β-lactams and DR gene pairs*** |  |  |
| % of knocked-out of DR and β-lactam^s^ pairs with reduced growth/cell death from gene (Fisher exact test p-value) | 88.46% (p=7.00x10^-5)^ | 75.34% (p=4.18x10^-7)^ |
