# Supplementary material for: Collateral Sensitivity to β-Lactam Drugs in Drug-Resistant Tuberculosis Is Driven by the Transcriptional Wiring of BlaI Operon Genes
Source: mSphere. 2021 May 28;6(3):e00245-21. doi: 10.1128/mSphere.00245-21 (PMC8265638; doi:10.1128/mSphere.00245-21)
Supplement: TABLE S3 [file msphere.00245-21-st003.docx]

**Supplementary Table S3.**

| **Gene** | **logFC** | **q value** |
| --- | --- | --- |
| Rv2843 | 1.391561 | 6.58890E-11 |
| Rv2164c | 2.030478 | 8.93282E-10 |
| Rv0226c | 1.356258 | 2.71257E-09 |
| Rv3246c | 3.202308 | 5.50817E-09 |
| Rv3320c | 1.729852 | 5.50817E-09 |
| Rv1566c | 3.152358 | 1.29576E-08 |
| Rv1220c | 1.455851 | 1.72762E-08 |
| Rv2816c | 2.323121 | 1.89210E-08 |
| Rv0997 | 1.316215 | 2.50995E-08 |
| Rv2550c | 2.497323 | 2.67818E-08 |
| Rv0599c | 2.324952 | 2.67818E-08 |
| Rv0883c | 1.9345 | 3.25604E-08 |
| Rv2148c | 1.653569 | 4.17239E-08 |
| Rv2380c | 1.927887 | 6.31350E-08 |
| Rv3267 | 1.966903 | 9.16475E-08 |
| Rv0526 | 1.832197 | 1.27370E-07 |
| Rv3342 | 1.254364 | 1.55373E-07 |
| Rv2525c | 2.562373 | 2.06174E-07 |
| Rv2350c | 1.291459 | 2.67263E-07 |
| Rv2864c | 1.789767 | 2.88312E-07 |
| Rv0394c | 1.847613 | 3.11416E-07 |
| Rv2810c | 2.030438 | 3.90872E-07 |
| Rv0626 | 1.498005 | 4.85342E-07 |
| Rv1683 | 1.752688 | 5.84939E-07 |
| Rv3678c | 1.744582 | 7.72077E-07 |
| Rv1420 | 1.537033 | 8.59423E-07 |
| Rv0731c | 1.894058 | 1.34193E-06 |
| Rv0598c | 1.783873 | 1.66191E-06 |
| Rv2163c | 3.269438 | 3.16114E-06 |
| Rv0217c | 1.166303 | 3.16114E-06 |
| Rv0707 | 1.39203 | 3.34753E-06 |
| Rv3268 | 1.604561 | 4.16264E-06 |
| Rv1157c | 2.589631 | 4.40906E-06 |
| Rv0582 | 1.318129 | 4.65137E-06 |
| Rv0747 | 1.709302 | 5.22308E-06 |
| Rv0867c | 2.083791 | 7.16970E-06 |
| Rv1873 | 1.907119 | 7.16970E-06 |
| Rv2335 | 1.352136 | 7.29736E-06 |
| Rv3587c | 2.28897 | 9.33024E-06 |
| Rv2165c | 2.208627 | 1.11073E-05 |
| Rv1350 | 1.675696 | 1.25703E-05 |
| Rv2552c | 1.192297 | 1.24391E-05 |
| Rv3321c | 1.088292 | 1.29041E-05 |
| Rv2756c | 2.044357 | 1.38202E-05 |
| Rv0519c | 1.822455 | 1.36025E-05 |
| Rv2256c | 2.257262 | 1.87836E-05 |
| Rv2956 | 1.56 | 2.79721E-05 |
| Rv2820c | 1.301455 | 2.79145E-05 |
| Rv1830 | 3.388 | 4.26160E-05 |
| Rv1240 | 1.421522 | 3.69615E-05 |
| Rv2110c | 1.198418 | 4.03512E-05 |
| Rv1061 | 1.08146 | 5.17054E-05 |
| Rv0380c | 1.214967 | 6.25930E-05 |
| Rv3417c | 1.959415 | 8.04452E-05 |
| Rv0527 | 2.038672 | 8.64042E-05 |
| Rv0418 | 1.367719 | 1.05836E-04 |
| Rv2732c | 1.261836 | 1.26053E-04 |
| Rv0344c | 1.053388 | 1.33033E-04 |
| Rv0935 | 1.164 | 1.36472E-04 |
| Rv2807 | 1.354981 | 1.41924E-04 |
| Rv1672c | 1.495041 | 1.52245E-04 |
| Rv1387 | 2.672274 | 1.54304E-04 |
| Rv0040c | 2.473358 | 1.53814E-04 |
| Rv0644c | 1.817862 | 2.00573E-04 |
| Rv2166c | 2.655403 | 1.71241E-04 |
| Rv3200c | 1.263583 | 1.80278E-04 |
| Rv2390c | 1.511712 | 1.87720E-04 |
| Rv2497c | 2.279273 | 1.96548E-04 |
| Rv0803 | 1.179707 | 2.13123E-04 |
| Rv1658 | 1.351207 | 2.16223E-04 |
| Rv0581 | 1.962925 | 2.14902E-04 |
| Rv3455c | 1.631814 | 2.29004E-04 |
| Rv2294 | 1.325524 | 2.29004E-04 |
| Rv1204c | 1.214016 | 2.42344E-04 |
| Rv1221 | 2.86691 | 2.48080E-04 |
| Rv1158c | 2.617621 | 2.49782E-04 |
| Rv1218c | 1.581672 | 2.59483E-04 |
| Rv3011c | 1.320197 | 2.64353E-04 |
| Rv2898c | 1.047103 | 3.19291E-04 |
| Rv3907c | 1.151833 | 3.23186E-04 |
| Rv2530c | 1.006019 | 3.32963E-04 |
| Rv0727c | 1.11908 | 3.42775E-04 |
| Rv2202c | 1.152485 | 3.50446E-04 |
| Rv2078 | 1.018821 | 3.86611E-04 |
| Rv3604c | 1.216203 | 3.58623E-04 |
| Rv2069 | 1.432582 | 3.58623E-04 |
| Rv2782c | 1.619121 | 3.84572E-04 |
| Rv0249c | 1.720015 | 3.89539E-04 |
| Rv2887 | 1.475655 | 4.17050E-04 |
| Rv1592c | 3.293606 | 4.11294E-04 |
| Rv3413c | 1.901123 | 4.24422E-04 |
| Rv2602 | 1.069032 | 4.26384E-04 |
| Rv2429 | 3.987106 | 4.40149E-04 |
| Rv1030 | 1.555935 | 5.74145E-04 |
| Rv2888c | 1.420667 | 4.91641E-04 |
| Rv0681 | 1.112625 | 5.22379E-04 |
| Rv2975c | 1.507086 | 5.34363E-04 |
| Rv1679 | 1.129612 | 5.42717E-04 |
| Rv2428 | 3.743776 | 5.56459E-04 |
| Rv0660c | 1.933552 | 6.01753E-04 |
| Rv1839c | 1.017698 | 6.04391E-04 |
| Rv1578c | 1.829721 | 6.15550E-04 |
| Rv2815c | 1.230149 | 6.15550E-04 |
| Rv2927c | 2.419385 | 6.35265E-04 |
| Rv3399 | 1.541383 | 6.58410E-04 |
| Rv2168c | 1.080727 | 6.67872E-04 |
| Rv2671 | 1.377433 | 6.84736E-04 |
| Rv1538c | 1.074086 | 7.47049E-04 |
| Rv1697 | 2.191574 | 8.37538E-04 |
| Rv2865 | 2.027422 | 8.73348E-04 |
| Rv3465 | 1.234368 | 8.96248E-04 |
| Rv0831c | 1.066727 | 9.48263E-04 |
| Rv1932 | 1.264514 | 1.10171E-03 |
| Rv2527 | 2.281787 | 1.02803E-03 |
| Rv2150c | 1.859375 | 1.10171E-03 |
| Rv0044c | 1.121239 | 1.11009E-03 |
| Rv1388 | 1.215121 | 1.13435E-03 |
| Rv1674c | 1.128864 | 1.15215E-03 |
| Rv1404 | 1.069045 | 1.25116E-03 |
| Rv0457c | 1.260567 | 1.38007E-03 |
| Rv0659c | 1.835288 | 1.45452E-03 |
| Rv2009 | 1.138015 | 1.48326E-03 |
| Rv1386 | 2.292636 | 1.49740E-03 |
| Rv0677c | 1.567597 | 1.50027E-03 |
| Rv3812 | 1.983492 | 1.61668E-03 |
| Rv0728c | 1.101297 | 1.63917E-03 |
| Rv2052c | 2.129299 | 2.19435E-03 |
| Rv2467 | 1.148476 | 1.77767E-03 |
| Rv2747 | 1.2045 | 1.91229E-03 |
| Rv2796c | 1.248484 | 1.94512E-03 |
| Rv2103c | 1.046721 | 2.33166E-03 |
| Rv1623c | 2.089273 | 2.33166E-03 |
| Rv3276c | 1.395509 | 2.46694E-03 |
| Rv3258c | 2.093429 | 2.56594E-03 |
| Rv2223c | 1.051152 | 2.89544E-03 |
| Rv1698 | 1.870138 | 2.66006E-03 |
| Rv1982c | 1.299897 | 2.89544E-03 |
| Rv2147c | 2.179682 | 2.91870E-03 |
| Rv3414c | 2.097154 | 2.99090E-03 |
| Rv1544 | 1.173138 | 3.35206E-03 |
| Rv3209 | 1.413032 | 3.41082E-03 |
| Rv2216 | 1.222194 | 3.50694E-03 |
| Rv1370c | 1.030815 | 3.63733E-03 |
| Rv2710 | 3.30603 | 3.63733E-03 |
| Rv1072 | 1.688356 | 3.90838E-03 |
| Rv0285 | 1.276415 | 4.03826E-03 |
| Rv3843c | 1.237379 | 4.07003E-03 |
| Rv0059 | 1.215164 | 4.25309E-03 |
| Rv3248c | 1.892925 | 4.33310E-03 |
| Rv3330 | 1.377113 | 4.36163E-03 |
| Rv1042c | 1.257955 | 4.38423E-03 |
| Rv2944 | 1.31628 | 4.71177E-03 |
| Rv1755c | 1.326776 | 5.90644E-03 |
| Rv2648 | 1.075433 | 4.67921E-03 |
| Rv2452c | 1.161895 | 4.78595E-03 |
| Rv0592 | 1.6123 | 6.57201E-03 |
| Rv3608c | 1.014386 | 5.34969E-03 |
| Rv2676c | 1.001185 | 5.32347E-03 |
| Rv2606c | 1.006985 | 5.51580E-03 |
| Rv3324c | 1.623575 | 6.23573E-03 |
| Rv2617c | 1.891291 | 5.96414E-03 |
| Rv0286 | 1.608017 | 6.89817E-03 |
| Rv0074 | 1.281284 | 7.12757E-03 |
| Rv3269 | 3.335985 | 7.49985E-03 |
| Rv1595 | 1.31509 | 7.49985E-03 |
| Rv3640c | 1.622538 | 9.46152E-03 |
| Rv2145c | 1.995642 | 7.96969E-03 |
| Rv2351c | 1.121161 | 8.20589E-03 |
| Rv0023 | 1.047016 | 8.25049E-03 |
| Rv2595 | 1.008288 | 8.25049E-03 |
| Rv1963c | 1.176897 | 8.77412E-03 |
| Rv1981c | 1.49285 | 9.02474E-03 |
| Rv2276 | 1.126642 | 9.17664E-03 |
| Rv1999c | 1.38696 | 9.54705E-03 |
| Rv2745c | 1.242418 | 9.37219E-03 |
| Rv1846c | 1.508746 | 9.53535E-03 |
| Rv3341 | 1.002984 | 9.54705E-03 |
| Rv0483 | 1.673164 | 9.65702E-03 |
| Rv0795 | 1.376048 | 1.02945E-02 |
| Rv2033c | 1.192049 | 1.12764E-02 |
| Rv3353c | 1.172881 | 1.17364E-02 |
| Rv1132 | 1.050951 | 1.30409E-02 |
| Rv3273 | 1.106138 | 1.26744E-02 |
| Rv0723 | 1.245953 | 1.28220E-02 |
| Rv2515c | 1.267317 | 1.32678E-02 |
| Rv2835c | 1.123352 | 1.41197E-02 |
| Rv0440 | 3.870791 | 1.41219E-02 |
| Rv0406c | 1.225019 | 1.45586E-02 |
| Rv0218 | 1.00977 | 1.46930E-02 |
| Rv1406 | 1.178133 | 1.59793E-02 |
| Rv2987c | 1.344803 | 1.49434E-02 |
| Rv0240 | 1.739508 | 1.91666E-02 |
| Rv3310 | 1.589833 | 1.55374E-02 |
| Rv2107 | 1.003825 | 1.56396E-02 |
| Rv2926c | 1.36894 | 1.56005E-02 |
| Rv3047c | 1.099788 | 1.62713E-02 |
| Rv1062 | 1.16653 | 2.01386E-02 |
| Rv2466c | 1.757879 | 1.62287E-02 |
| Rv2986c | 2.111627 | 1.62287E-02 |
| Rv2790c | 2.092516 | 1.63050E-02 |
| Rv2208 | 1.938138 | 1.64554E-02 |
| Rv0312 | 1.356092 | 1.68989E-02 |
| Rv2721c | 1.81154 | 1.70242E-02 |
| Rv0352 | 3.155299 | 1.69513E-02 |
| Rv2526 | 2.293507 | 1.72238E-02 |
| Rv0541c | 1.014821 | 1.72238E-02 |
| Rv0779c | 1.019567 | 1.77393E-02 |
| Rv2460c | 1.268209 | 1.77863E-02 |
| Rv2278 | 1.089267 | 1.85790E-02 |
| Rv2006 | 1.195712 | 1.86092E-02 |
| Rv0747 | 1.077968 | 1.93050E-02 |
| Rv2074 | 1.651242 | 1.92791E-02 |
| Rv1435c | 1.912754 | 2.01386E-02 |
| Rv0482 | 1.090698 | 2.67734E-02 |
| Rv2963 | 1.498108 | 2.16002E-02 |
| Rv2729c | 1.292492 | 2.18368E-02 |
| Rv2334 | 1.273954 | 2.20911E-02 |
| Rv0652 | 1.368661 | 2.32566E-02 |
| Rv0129c | 2.509439 | 2.32375E-02 |
| Rv0103c | 1.07609 | 2.33763E-02 |
| Rv0420c | 1.037883 | 2.40240E-02 |
| Rv3395c | 1.843214 | 2.65753E-02 |
| Rv3170 | 1.049737 | 2.51193E-02 |
| Rv2933 | 1.332493 | 2.53894E-02 |
| Rv0928 | 1.104848 | 3.32456E-02 |
| Rv0676c | 1.635864 | 2.64012E-02 |
| Rv0251c | 3.876194 | 2.68239E-02 |
| Rv1277 | 1.326758 | 2.73106E-02 |
| Rv2108 | 1.298038 | 2.85727E-02 |
| Rv1400c | 1.338019 | 2.86163E-02 |
| Rv3008 | 1.005905 | 2.82394E-02 |
| Rv3050c | 1.186179 | 2.82148E-02 |
| Rv2744c | 1.382152 | 2.82643E-02 |
| Rv0706 | 1.500567 | 2.87660E-02 |
| Rv0641 | 1.427091 | 2.87820E-02 |
| Rv2672 | 1.038742 | 2.99847E-02 |
| Rv1307 | 1.172682 | 3.00313E-02 |
| Rv3385c | 1.072288 | 3.08119E-02 |
| Rv3810 | 2.052754 | 3.11398E-02 |
| Rv2737c | 1.806875 | 3.17441E-02 |
| Rv3503c | 1.665094 | 3.17441E-02 |
| Rv2576c | 1.528015 | 3.17324E-02 |
| Rv3180c | 1.034967 | 3.23379E-02 |
| Rv2383c | 1.110537 | 3.27130E-02 |
| Rv1884c | 1.73406 | 3.24453E-02 |
| Rv2923c | 1.0275 | 3.30443E-02 |
| Rv1076 | 1.327597 | 3.32440E-02 |
| Rv1690 | 1.748938 | 3.41445E-02 |
| Rv3628 | 1.027258 | 3.41445E-02 |
| Rv3335c | 1.275326 | 3.57208E-02 |
| Rv3270 | 2.323212 | 3.49813E-02 |
| Rv3585 | 1.238274 | 3.51596E-02 |
| Rv2593c | 1.109536 | 3.79226E-02 |
| Rv3550 | 1.12845 | 3.81980E-02 |
| Rv3426 | 1.058373 | 3.88206E-02 |
| Rv1203c | 1.129036 | 3.98835E-02 |
| Rv0898c | 1.28647 | 4.10267E-02 |
| Rv0667 | 1.090015 | 4.10267E-02 |
| Rv0848 | 1.530727 | 4.13583E-02 |
| Rv3075c | 1.369269 | 4.22770E-02 |
| Rv2930 | 1.761469 | 4.28656E-02 |
| Rv0873 | 1.050629 | 4.43704E-02 |
| Rv1070c | 1.152123 | 4.48468E-02 |
| Rv3260c | 1.881413 | 4.55973E-02 |
| Rv0243 | 1.238179 | 4.57205E-02 |
| Rv0678 | 2.468701 | 4.75927E-02 |
| Rv2734 | 1.477083 | 4.96039E-02 |
| Rv0743c | 1.013883 | 4.97446E-02 |
| Rv2032 | -5.5439 | 3.52462E-23 |
| Rv2961 | -2.84622 | 3.52462E-23 |
| Rv2626c | -4.63675 | 1.77998E-17 |
| Rv3289c | -2.80849 | 5.63948E-17 |
| Rv2627c | -4.72756 | 5.63948E-17 |
| Rv0569 | -4.6463 | 4.38913E-16 |
| Rv1180 | -2.58492 | 4.89853E-15 |
| Rv1181 | -3.51131 | 1.28065E-14 |
| Rv2628 | -4.23606 | 1.28065E-14 |
| Rv3588c | -2.28301 | 1.23287E-13 |
| Rv0079 | -3.05995 | 1.83084E-13 |
| Rv1868 | -2.00592 | 5.45433E-13 |
| Rv3237c | -1.66984 | 3.06627E-12 |
| Rv1183 | -2.45334 | 1.20832E-11 |
| Rv2031c | -5.46669 | 1.20832E-11 |
| Rv1365c | -2.42718 | 3.80576E-11 |
| Rv1639c | -1.95318 | 5.48796E-11 |
| Rv2129c | -2.20653 | 1.17296E-10 |
| Rv2342 | -2.2639 | 1.30368E-10 |
| Rv3134c | -2.64626 | 1.57825E-10 |
| Rv2632c | -2.94918 | 4.54926E-10 |
| Rv1393c | -2.19799 | 5.03508E-10 |
| Rv1368 | -2.1523 | 1.27707E-09 |
| Rv1200 | -1.96848 | 1.67649E-09 |
| Rv0319 | -2.48832 | 1.77602E-09 |
| Rv1184c | -3.07811 | 2.13693E-09 |
| Rv0740 | -1.95188 | 3.77598E-09 |
| Rv2914c | -1.8062 | 3.99190E-09 |
| Rv3572 | -1.73886 | 3.99190E-09 |
| Rv0696 | -2.73439 | 5.34795E-09 |
| Rv1829 | -1.93582 | 5.50817E-09 |
| Rv2158c | -2.18015 | 5.50817E-09 |
| Rv3613c | -2.73571 | 6.01544E-09 |
| Rv2007c | -4.00009 | 1.63244E-08 |
| Rv3290c | -5.00827 | 2.97480E-08 |
| Rv1161 | -1.54798 | 3.39808E-08 |
| Rv2668 | -1.7272 | 3.63218E-08 |
| Rv3614c | -2.96831 | 3.95418E-08 |
| Rv0739 | -1.98802 | 4.17239E-08 |
| Rv2781c | -1.46611 | 4.71250E-08 |
| Rv1724c | -1.60044 | 6.31350E-08 |
| Rv1163 | -1.89727 | 6.31350E-08 |
| Rv3863 | -2.32623 | 6.34355E-08 |
| Rv2764c | -1.96811 | 8.42918E-08 |
| Rv2608 | -1.64423 | 9.44809E-08 |
| Rv1194c | -1.42521 | 1.50280E-07 |
| Rv3616c | -3.0102 | 1.54859E-07 |
| Rv0165c | -1.8122 | 1.66039E-07 |
| Rv2777c | -4.01895 | 1.69469E-07 |
| Rv2005c | -1.89791 | 1.71423E-07 |
| Rv2398c | -1.55617 | 1.79687E-07 |
| Rv3749c | -2.58464 | 1.94786E-07 |
| Rv3291c | -2.07277 | 5.33615E-07 |
| Rv1172c | -1.93189 | 5.91262E-07 |
| Rv2177c | -1.408 | 6.56150E-07 |
| Rv3840 | -1.89363 | 7.17613E-07 |
| Rv0001 | -2.06352 | 1.34193E-06 |
| Rv3824c | -1.90934 | 1.91616E-06 |
| Rv3686c | -2.75157 | 2.02203E-06 |
| Rv3089 | -1.96462 | 1.99044E-06 |
| Rv1360 | -1.72737 | 2.00856E-06 |
| Rv3479 | -2.33901 | 2.39343E-06 |
| Rv2336 | -2.75239 | 3.16114E-06 |
| Rv2340c | -1.58339 | 2.83121E-06 |
| Rv3633 | -1.53277 | 2.92548E-06 |
| Rv0070c | -1.30988 | 3.12959E-06 |
| Rv2008c | -1.78076 | 3.25850E-06 |
| Rv2159c | -1.52548 | 4.05127E-06 |
| Rv0948c | -1.62809 | 7.13957E-06 |
| Rv3896c | -1.17739 | 7.13957E-06 |
| Rv3712 | -1.79855 | 8.83837E-06 |
| Rv2304c | -1.15221 | 8.74425E-06 |
| Rv3750c | -1.74025 | 9.23486E-06 |
| Rv3491 | -1.67404 | 1.07977E-05 |
| Rv1439c | -1.29949 | 1.07977E-05 |
| Rv3698 | -1.08734 | 1.16129E-05 |
| Rv2424c | -1.54647 | 1.24391E-05 |
| Rv1996 | -1.70677 | 1.24391E-05 |
| Rv2348c | -1.52594 | 1.72994E-05 |
| Rv3825c | -1.93681 | 3.01217E-05 |
| Rv2623 | -2.75407 | 3.09799E-05 |
| Rv2375 | -1.52072 | 3.09029E-05 |
| Rv0679c | -1.33553 | 3.18149E-05 |
| Rv3733c | -1.49717 | 3.18149E-05 |
| Rv2303c | -1.15989 | 3.28288E-05 |
| Rv1425 | -1.4478 | 4.03512E-05 |
| Rv0535 | -1.90233 | 4.79928E-05 |
| Rv2352c | -1.35546 | 5.95665E-05 |
| Rv1008 | -1.33525 | 8.96010E-05 |
| Rv3487c | -2.99924 | 8.26397E-05 |
| Rv3390 | -1.26484 | 8.64042E-05 |
| Rv2290 | -1.29623 | 8.64042E-05 |
| Rv2663 | -1.95138 | 8.64042E-05 |
| Rv0827c | -1.96276 | 9.11710E-05 |
| Rv2406c | -1.45223 | 1.08359E-04 |
| Rv0713 | -1.36607 | 1.10917E-04 |
| Rv3254 | -2.00415 | 1.22630E-04 |
| Rv3383c | -1.11375 | 1.40962E-04 |
| Rv3234c | -1.51708 | 1.42066E-04 |
| Rv3822 | -2.43538 | 1.54304E-04 |
| Rv3848 | -1.24161 | 1.55413E-04 |
| Rv1926c | -1.14131 | 1.87720E-04 |
| Rv1893 | -2.47629 | 1.88377E-04 |
| Rv3331 | -1.05877 | 1.95070E-04 |
| Rv2160c | -2.07617 | 1.94626E-04 |
| Rv3701c | -1.78407 | 1.96407E-04 |
| Rv3501c | -1.37366 | 2.10646E-04 |
| Rv2459 | -1.36066 | 2.10646E-04 |
| Rv2629 | -1.36224 | 2.13139E-04 |
| Rv0990c | -2.16664 | 2.23082E-04 |
| Rv3087 | -2.01056 | 2.27166E-04 |
| Rv1813c | -2.246 | 2.29004E-04 |
| Rv2686c | -1.01876 | 2.83799E-04 |
| Rv3612c | -1.73197 | 3.03954E-04 |
| Rv3849 | -1.29693 | 3.18020E-04 |
| Rv3230c | -1.50144 | 3.56730E-04 |
| Rv2205c | -1.19162 | 3.58623E-04 |
| Rv3165c | -1.28625 | 3.86611E-04 |
| Rv2654c | -1.14203 | 5.00889E-04 |
| Rv1318c | -1.00398 | 4.11294E-04 |
| Rv2138 | -1.23014 | 4.13593E-04 |
| Rv2483c | -1.25548 | 4.54372E-04 |
| Rv3287c | -1.775 | 5.74145E-04 |
| Rv2333c | -1.06668 | 5.94801E-04 |
| Rv2558 | -2.08738 | 6.13333E-04 |
| Rv2266 | -1.22487 | 6.13476E-04 |
| Rv2950c | -1.83659 | 6.25053E-04 |
| Rv1896c | -1.07175 | 6.30505E-04 |
| Rv1733c | -1.87319 | 7.76569E-04 |
| Rv3294 | -1.53283 | 7.46494E-04 |
| Rv3729 | -1.2977 | 7.53438E-04 |
| Rv3091 | -1.11489 | 7.70816E-04 |
| Rv3868 | -1.29105 | 8.19307E-04 |
| Rv1162 | -1.33668 | 8.61898E-04 |
| Rv3387 | -1.90475 | 8.92347E-04 |
| Rv2338c | -1.67175 | 1.06391E-03 |
| Rv3784 | -1.13516 | 1.11438E-03 |
| Rv3615c | -2.31446 | 1.10926E-03 |
| Rv2135c | -1.19045 | 1.19377E-03 |
| Rv3288c | -1.26063 | 1.52002E-03 |
| Rv2472 | -1.49215 | 1.96681E-03 |
| Rv1975 | -1.10567 | 1.74831E-03 |
| Rv0076c | -1.35507 | 1.75445E-03 |
| Rv1361c | -2.18753 | 2.02129E-03 |
| Rv3371 | -1.38406 | 2.19192E-03 |
| Rv2289 | -1.20013 | 2.34022E-03 |
| Rv1263 | -1.31202 | 2.98187E-03 |
| Rv3823c | -1.14106 | 2.45194E-03 |
| Rv3674c | -1.55924 | 2.46749E-03 |
| Rv0471c | -1.25264 | 2.59141E-03 |
| Rv2083 | -1.17763 | 3.21871E-03 |
| Rv3755c | -1.12217 | 2.77602E-03 |
| Rv3326 | -1.10757 | 2.89467E-03 |
| Rv0064 | -1.23637 | 3.00887E-03 |
| Rv2633c | -2.24058 | 3.03666E-03 |
| Rv2590 | -1.89818 | 3.06907E-03 |
| Rv0458 | -1.32458 | 3.58682E-03 |
| Rv2583c | -1.45601 | 4.00584E-03 |
| Rv0920c | -1.51247 | 3.98015E-03 |
| Rv0811c | -1.00427 | 4.09386E-03 |
| Rv3499c | -1.24085 | 4.17352E-03 |
| Rv2157c | -1.41902 | 4.33310E-03 |
| Rv1027c | -1.21747 | 4.17719E-03 |
| Rv2519 | -1.09836 | 4.33310E-03 |
| Rv2161c | -1.04814 | 4.32434E-03 |
| Rv1770 | -1.15756 | 4.33310E-03 |
| Rv3753c | -1.16492 | 4.45629E-03 |
| Rv1164 | -1.52802 | 4.67921E-03 |
| Rv3826 | -1.3108 | 4.71177E-03 |
| Rv2046 | -1.08205 | 4.82722E-03 |
| Rv3910 | -1.10592 | 4.83696E-03 |
| Rv0909 | -1.00485 | 5.96414E-03 |
| Rv3526 | -1.20441 | 5.96414E-03 |
| Rv0493c | -1.08426 | 6.20943E-03 |
| Rv3159c | -1.31494 | 8.20340E-03 |
| Rv2557 | -1.75094 | 6.97332E-03 |
| Rv1182 | -1.80198 | 7.61254E-03 |
| Rv1870c | -1.51888 | 7.61254E-03 |
| Rv3478 | -1.92415 | 8.29101E-03 |
| Rv1980c | -1.33367 | 8.97073E-03 |
| Rv0509 | -1.02686 | 8.77140E-03 |
| Rv0030 | -1.14135 | 1.13851E-02 |
| Rv1065 | -1.27552 | 1.17133E-02 |
| Rv1990c | -1.15806 | 1.24485E-02 |
| Rv2688c | -1.03753 | 1.28422E-02 |
| Rv0942 | -1.3593 | 1.66308E-02 |
| Rv3778c | -1.07794 | 1.34761E-02 |
| Rv0257c | -1.26588 | 1.34761E-02 |
| Rv1797 | -1.49575 | 1.38139E-02 |
| Rv1640c | -1.0028 | 1.39881E-02 |
| Rv1196 | -1.29922 | 1.39881E-02 |
| Rv3416 | -1.36565 | 1.47155E-02 |
| Rv1284 | -1.44761 | 1.48992E-02 |
| Rv2025c | -1.00408 | 1.64554E-02 |
| Rv3477 | -2.16492 | 1.80982E-02 |
| Rv3235 | -1.50942 | 1.93050E-02 |
| Rv3855 | -1.80009 | 1.89106E-02 |
| Rv2664 | -1.24372 | 2.03972E-02 |
| Rv2715 | -1.11655 | 2.10715E-02 |
| Rv1887 | -1.07661 | 2.13286E-02 |
| Rv2641 | -1.67353 | 2.19482E-02 |
| Rv1986 | -1.11657 | 2.93288E-02 |
| Rv1185c | -1.18321 | 2.41741E-02 |
| Rv1353c | -1.20856 | 3.25255E-02 |
| Rv0886 | -1.29233 | 3.73963E-02 |
| Rv0263c | -1.01131 | 3.09965E-02 |
| Rv3365c | -1.00036 | 3.27130E-02 |
| Rv1660 | -1.0132 | 3.37559E-02 |
| Rv1994c | -1.1208 | 3.36306E-02 |
| Rv1171 | -1.0355 | 3.50909E-02 |
| Rv2301 | -1.17529 | 3.79554E-02 |
| Rv3763 | -1.18781 | 4.08838E-02 |
| Rv0682 | -1.36268 | 4.08838E-02 |
| Rv2371 | -1.10645 | 4.35265E-02 |
| Rv3469c | -1.35706 | 4.57146E-02 |
