# Supplementary figures and images for: Collateral Sensitivity to β-Lactam Drugs in Drug-Resistant Tuberculosis Is Driven by the Transcriptional Wiring of BlaI Operon Genes
Source: mSphere. 2021 May 28;6(3):e00245-21. doi: 10.1128/mSphere.00245-21 (PMC8265638; doi:10.1128/mSphere.00245-21)

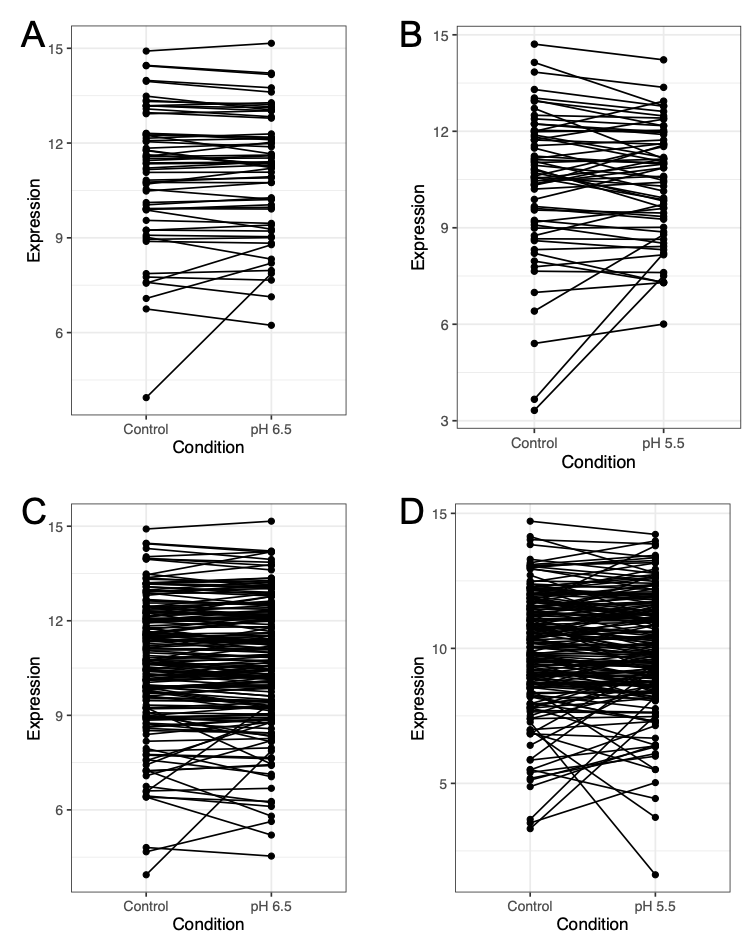


**Supplementary Figure S1.**

Supplement: FIG S1 [file msphere.00245-21-sf001.docx]

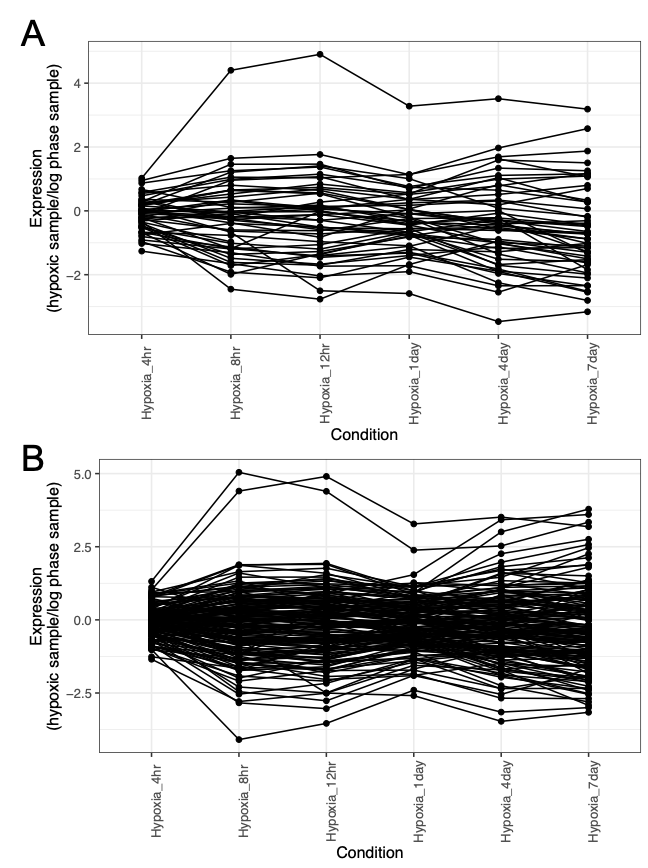


**Supplementary Figure S2.**

Supplement: FIG S2 [file msphere.00245-21-sf002.docx]

**
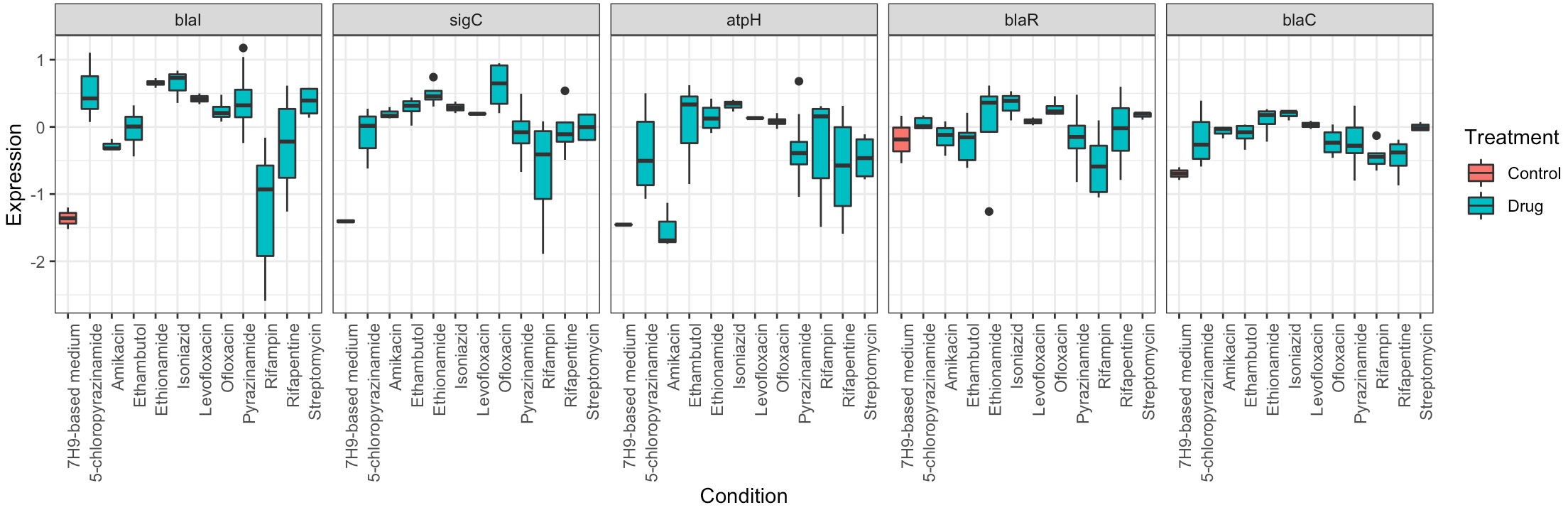
**

**Supplementary Figure S3.**

Supplement: FIG S3 [file msphere.00245-21-sf003.docx]

**
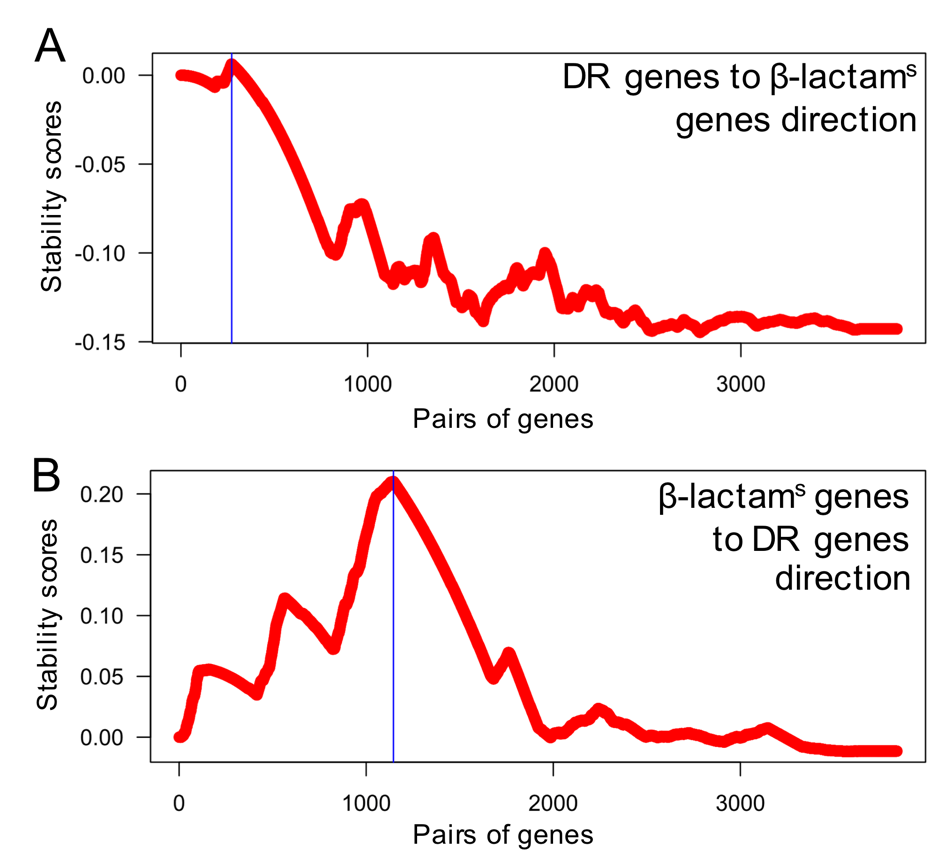
**

**Supplementary Figure S4.**

Supplement: FIG S4 [file msphere.00245-21-sf004.docx]
