# Supplementary material for: Collateral Sensitivity to β-Lactam Drugs in Drug-Resistant Tuberculosis Is Driven by the Transcriptional Wiring of BlaI Operon Genes
Source: mSphere. 2021 May 28;6(3):e00245-21. doi: 10.1128/mSphere.00245-21 (PMC8265638; doi:10.1128/mSphere.00245-21)
Supplement: TABLE S4 [file msphere.00245-21-st004.docx]

**Supplementary Table S4.**

| **Gene1** | **Name1** | **Class1** | **Drug1** | **Gene2** | **Name2** | **Class2** | **Drug2** | **Database** | **Score** |
| --- | --- | --- | --- | --- | --- | --- | --- | --- | --- |
| Rv0011c | Rv0011c | β-lactam^s^ gene | NA | Rv2150c | ftsZ | β-lactam^s^ gene | NA | PPI | 805 |
| Rv0014c | pknB | β-lactam^s^ gene | NA | Rv0015c | pknA | β-lactam^s^ gene | NA | PPI | 974 |
| Rv0014c | pknB | β-lactam^s^ gene | NA | Rv2150c | ftsZ | β-lactam^s^ gene | NA | PPI | 721 |
| Rv0014c | pknB | β-lactam^s^ gene | NA | Rv2154c | ftsW | β-lactam^s^ gene | NA | PPI | 754 |
| Rv0014c | pknB | β-lactam^s^ gene | NA | Rv2163c | pbpB | β-lactam^s^ gene | NA | PPI | 725 |
| Rv0014c | pknB | β-lactam^s^ gene | NA | Rv2927c | Rv2927c | β-lactam^s^ gene | NA | PPI | 701 |
| Rv0015c | pknA | β-lactam^s^ gene | NA | Rv2145c | wag31 | β-lactam^s^ gene | NA | PPI | 940 |
| Rv0015c | pknA | β-lactam^s^ gene | NA | Rv2150c | ftsZ | β-lactam^s^ gene | NA | PPI | 915 |
| Rv0050 | ponA1 | β-lactam^s^ gene | NA | Rv2152c | murC | β-lactam^s^ gene | NA | PPI | 773 |
| Rv0050 | ponA1 | β-lactam^s^ gene | NA | Rv2154c | ftsW | β-lactam^s^ gene | NA | PPI | 911 |
| Rv0050 | ponA1 | β-lactam^s^ gene | NA | Rv2156c | murX | β-lactam^s^ gene | NA | PPI | 747 |
| Rv0050 | ponA1 | β-lactam^s^ gene | NA | Rv2163c | pbpB | β-lactam^s^ gene | NA | PPI | 993 |
| Rv0050 | ponA1 | β-lactam^s^ gene | NA | Rv2864c | Rv2864c | β-lactam^s^ gene | NA | PPI | 965 |
| Rv0050 | ponA1 | β-lactam^s^ gene | NA | Rv2911 | dacB2 | β-lactam^s^ gene | NA | PPI | 804 |
| Rv0050 | ponA1 | β-lactam^s^ gene | NA | Rv3330 | dacB1 | β-lactam^s^ gene | NA | PPI | 804 |
| Rv0050 | ponA1 | β-lactam^s^ gene | NA | Rv3627c | Rv3627c | β-lactam^s^ gene | NA | PPI | 780 |
| Rv0112 | gca | β-lactam^s^ gene | NA | Rv0113 | gmhA | β-lactam^s^ gene | NA | PPI | 813 |
| Rv0112 | gca | β-lactam^s^ gene | NA | Rv1503c | Rv1503c | β-lactam^s^ gene | NA | PPI | 808 |
| Rv1024 | Rv1024 | β-lactam^s^ gene | NA | Rv1025 | Rv1025 | β-lactam^s^ gene | NA | PPI | 974 |
| Rv1303 | Rv1303 | β-lactam^s^ gene | NA | Rv1304 | atpB | β-lactam^s^ gene | NA | PPI | 873 |
| Rv1303 | Rv1303 | β-lactam^s^ gene | NA | Rv1305 | atpE | β-lactam^s^ gene | NA | PPI | 704 |
| Rv1304 | atpB | β-lactam^s^ gene | NA | Rv1305 | atpE | β-lactam^s^ gene | NA | PPI | 999 |
| Rv1304 | atpB | β-lactam^s^ gene | NA | Rv1306 | atpF | β-lactam^s^ gene | NA | PPI | 998 |
| Rv1304 | atpB | β-lactam^s^ gene | NA | Rv1307 | atpH | β-lactam^s^ gene | NA | PPI | 999 |
| Rv1304 | atpB | β-lactam^s^ gene | NA | Rv1308 | atpA | β-lactam^s^ gene | NA | PPI | 999 |
| Rv1304 | atpB | β-lactam^s^ gene | NA | Rv1309 | atpG | β-lactam^s^ gene | NA | PPI | 999 |
| Rv1304 | atpB | β-lactam^s^ gene | NA | Rv1310 | atpD | β-lactam^s^ gene | NA | PPI | 999 |
| Rv1304 | atpB | β-lactam^s^ gene | NA | Rv1311 | atpC | β-lactam^s^ gene | NA | PPI | 998 |
| Rv1304 | atpB | β-lactam^s^ gene | NA | Rv3921c | Rv3921c | β-lactam^s^ gene | NA | PPI | 807 |
| Rv1305 | atpE | β-lactam^s^ gene | NA | Rv1306 | atpF | β-lactam^s^ gene | NA | PPI | 999 |
| Rv1305 | atpE | β-lactam^s^ gene | NA | Rv1307 | atpH | β-lactam^s^ gene | NA | PPI | 999 |
| Rv1305 | atpE | β-lactam^s^ gene | NA | Rv1308 | atpA | β-lactam^s^ gene | NA | PPI | 999 |
| Rv1305 | atpE | β-lactam^s^ gene | NA | Rv1309 | atpG | β-lactam^s^ gene | NA | PPI | 999 |
| Rv1305 | atpE | β-lactam^s^ gene | NA | Rv1310 | atpD | β-lactam^s^ gene | NA | PPI | 998 |
| Rv1305 | atpE | β-lactam^s^ gene | NA | Rv1311 | atpC | β-lactam^s^ gene | NA | PPI | 998 |
| Rv1305 | atpE | β-lactam^s^ gene | NA | Rv3921c | Rv3921c | β-lactam^s^ gene | NA | PPI | 936 |
| Rv1306 | atpF | β-lactam^s^ gene | NA | Rv1307 | atpH | β-lactam^s^ gene | NA | PPI | 999 |
| Rv1306 | atpF | β-lactam^s^ gene | NA | Rv1308 | atpA | β-lactam^s^ gene | NA | PPI | 999 |
| Rv1306 | atpF | β-lactam^s^ gene | NA | Rv1309 | atpG | β-lactam^s^ gene | NA | PPI | 996 |
| Rv1306 | atpF | β-lactam^s^ gene | NA | Rv1310 | atpD | β-lactam^s^ gene | NA | PPI | 998 |
| Rv1306 | atpF | β-lactam^s^ gene | NA | Rv1311 | atpC | β-lactam^s^ gene | NA | PPI | 994 |
| Rv1307 | atpH | β-lactam^s^ gene | NA | Rv1308 | atpA | β-lactam^s^ gene | NA | PPI | 999 |
| Rv1307 | atpH | β-lactam^s^ gene | NA | Rv1309 | atpG | β-lactam^s^ gene | NA | PPI | 999 |
| Rv1307 | atpH | β-lactam^s^ gene | NA | Rv1310 | atpD | β-lactam^s^ gene | NA | PPI | 999 |
| Rv1307 | atpH | β-lactam^s^ gene | NA | Rv1311 | atpC | β-lactam^s^ gene | NA | PPI | 999 |
| Rv1308 | atpA | β-lactam^s^ gene | NA | Rv1309 | atpG | β-lactam^s^ gene | NA | PPI | 999 |
| Rv1308 | atpA | β-lactam^s^ gene | NA | Rv1310 | atpD | β-lactam^s^ gene | NA | PPI | 999 |
| Rv1308 | atpA | β-lactam^s^ gene | NA | Rv1311 | atpC | β-lactam^s^ gene | NA | PPI | 999 |
| Rv1309 | atpG | β-lactam^s^ gene | NA | Rv1310 | atpD | β-lactam^s^ gene | NA | PPI | 999 |
| Rv1309 | atpG | β-lactam^s^ gene | NA | Rv1311 | atpC | β-lactam^s^ gene | NA | PPI | 999 |
| Rv1310 | atpD | β-lactam^s^ gene | NA | Rv1311 | atpC | β-lactam^s^ gene | NA | PPI | 999 |
| Rv1310 | atpD | β-lactam^s^ gene | NA | Rv1312 | Rv1312 | β-lactam^s^ gene | NA | PPI | 822 |
| Rv1311 | atpC | β-lactam^s^ gene | NA | Rv1312 | Rv1312 | β-lactam^s^ gene | NA | PPI | 856 |
| Rv1338 | murI | β-lactam^s^ gene | NA | Rv2155c | murD | β-lactam^s^ gene | NA | PPI | 990 |
| Rv1338 | murI | β-lactam^s^ gene | NA | Rv2158c | murE | β-lactam^s^ gene | NA | PPI | 751 |
| Rv1338 | murI | β-lactam^s^ gene | NA | Rv2981c | ddlA | β-lactam^s^ gene | NA | PPI | 729 |
| Rv2093c | tatC | β-lactam^s^ gene | NA | Rv2094c | tatA | β-lactam^s^ gene | NA | PPI | 987 |
| Rv2145c | wag31 | β-lactam^s^ gene | NA | Rv2147c | Rv2147c | β-lactam^s^ gene | NA | PPI | 816 |
| Rv2145c | wag31 | β-lactam^s^ gene | NA | Rv2150c | ftsZ | β-lactam^s^ gene | NA | PPI | 860 |
| Rv2147c | Rv2147c | β-lactam^s^ gene | NA | Rv2150c | ftsZ | β-lactam^s^ gene | NA | PPI | 998 |
| Rv2147c | Rv2147c | β-lactam^s^ gene | NA | Rv2151c | ftsQ | β-lactam^s^ gene | NA | PPI | 816 |
| Rv2147c | Rv2147c | β-lactam^s^ gene | NA | Rv2927c | Rv2927c | β-lactam^s^ gene | NA | PPI | 745 |
| Rv2150c | ftsZ | β-lactam^s^ gene | NA | Rv2151c | ftsQ | β-lactam^s^ gene | NA | PPI | 977 |
| Rv2150c | ftsZ | β-lactam^s^ gene | NA | Rv2152c | murC | β-lactam^s^ gene | NA | PPI | 862 |
| Rv2150c | ftsZ | β-lactam^s^ gene | NA | Rv2154c | ftsW | β-lactam^s^ gene | NA | PPI | 991 |
| Rv2150c | ftsZ | β-lactam^s^ gene | NA | Rv2155c | murD | β-lactam^s^ gene | NA | PPI | 959 |
| Rv2150c | ftsZ | β-lactam^s^ gene | NA | Rv2156c | murX | β-lactam^s^ gene | NA | PPI | 962 |
| Rv2150c | ftsZ | β-lactam^s^ gene | NA | Rv2157c | murF | β-lactam^s^ gene | NA | PPI | 847 |
| Rv2150c | ftsZ | β-lactam^s^ gene | NA | Rv2158c | murE | β-lactam^s^ gene | NA | PPI | 956 |
| Rv2150c | ftsZ | β-lactam^s^ gene | NA | Rv2163c | pbpB | β-lactam^s^ gene | NA | PPI | 927 |
| Rv2150c | ftsZ | β-lactam^s^ gene | NA | Rv2864c | Rv2864c | β-lactam^s^ gene | NA | PPI | 761 |
| Rv2150c | ftsZ | β-lactam^s^ gene | NA | Rv2981c | ddlA | β-lactam^s^ gene | NA | PPI | 879 |
| Rv2151c | ftsQ | β-lactam^s^ gene | NA | Rv2152c | murC | β-lactam^s^ gene | NA | PPI | 948 |
| Rv2151c | ftsQ | β-lactam^s^ gene | NA | Rv2154c | ftsW | β-lactam^s^ gene | NA | PPI | 994 |
| Rv2151c | ftsQ | β-lactam^s^ gene | NA | Rv2155c | murD | β-lactam^s^ gene | NA | PPI | 970 |
| Rv2151c | ftsQ | β-lactam^s^ gene | NA | Rv2156c | murX | β-lactam^s^ gene | NA | PPI | 959 |
| Rv2151c | ftsQ | β-lactam^s^ gene | NA | Rv2157c | murF | β-lactam^s^ gene | NA | PPI | 961 |
| Rv2151c | ftsQ | β-lactam^s^ gene | NA | Rv2158c | murE | β-lactam^s^ gene | NA | PPI | 956 |
| Rv2151c | ftsQ | β-lactam^s^ gene | NA | Rv2163c | pbpB | β-lactam^s^ gene | NA | PPI | 951 |
| Rv2151c | ftsQ | β-lactam^s^ gene | NA | Rv2864c | Rv2864c | β-lactam^s^ gene | NA | PPI | 951 |
| Rv2151c | ftsQ | β-lactam^s^ gene | NA | Rv2981c | ddlA | β-lactam^s^ gene | NA | PPI | 777 |
| Rv2151c | ftsQ | β-lactam^s^ gene | NA | Rv3921c | Rv3921c | β-lactam^s^ gene | NA | PPI | 933 |
| Rv2152c | murC | β-lactam^s^ gene | NA | Rv2154c | ftsW | β-lactam^s^ gene | NA | PPI | 992 |
| Rv2152c | murC | β-lactam^s^ gene | NA | Rv2155c | murD | β-lactam^s^ gene | NA | PPI | 999 |
| Rv2152c | murC | β-lactam^s^ gene | NA | Rv2156c | murX | β-lactam^s^ gene | NA | PPI | 994 |
| Rv2152c | murC | β-lactam^s^ gene | NA | Rv2157c | murF | β-lactam^s^ gene | NA | PPI | 982 |
| Rv2152c | murC | β-lactam^s^ gene | NA | Rv2158c | murE | β-lactam^s^ gene | NA | PPI | 997 |
| Rv2152c | murC | β-lactam^s^ gene | NA | Rv2163c | pbpB | β-lactam^s^ gene | NA | PPI | 959 |
| Rv2152c | murC | β-lactam^s^ gene | NA | Rv2864c | Rv2864c | β-lactam^s^ gene | NA | PPI | 896 |
| Rv2152c | murC | β-lactam^s^ gene | NA | Rv2981c | ddlA | β-lactam^s^ gene | NA | PPI | 991 |
| Rv2154c | ftsW | β-lactam^s^ gene | NA | Rv2155c | murD | β-lactam^s^ gene | NA | PPI | 995 |
| Rv2154c | ftsW | β-lactam^s^ gene | NA | Rv2156c | murX | β-lactam^s^ gene | NA | PPI | 998 |
| Rv2154c | ftsW | β-lactam^s^ gene | NA | Rv2157c | murF | β-lactam^s^ gene | NA | PPI | 988 |
| Rv2154c | ftsW | β-lactam^s^ gene | NA | Rv2158c | murE | β-lactam^s^ gene | NA | PPI | 996 |
| Rv2154c | ftsW | β-lactam^s^ gene | NA | Rv2163c | pbpB | β-lactam^s^ gene | NA | PPI | 999 |
| Rv2154c | ftsW | β-lactam^s^ gene | NA | Rv2864c | Rv2864c | β-lactam^s^ gene | NA | PPI | 969 |
| Rv2154c | ftsW | β-lactam^s^ gene | NA | Rv2981c | ddlA | β-lactam^s^ gene | NA | PPI | 967 |
| Rv2154c | ftsW | β-lactam^s^ gene | NA | Rv3682 | ponA2 | β-lactam^s^ gene | NA | PPI | 904 |
| Rv2155c | murD | β-lactam^s^ gene | NA | Rv2156c | murX | β-lactam^s^ gene | NA | PPI | 997 |
| Rv2155c | murD | β-lactam^s^ gene | NA | Rv2157c | murF | β-lactam^s^ gene | NA | PPI | 996 |
| Rv2155c | murD | β-lactam^s^ gene | NA | Rv2158c | murE | β-lactam^s^ gene | NA | PPI | 999 |
| Rv2155c | murD | β-lactam^s^ gene | NA | Rv2163c | pbpB | β-lactam^s^ gene | NA | PPI | 971 |
| Rv2155c | murD | β-lactam^s^ gene | NA | Rv2864c | Rv2864c | β-lactam^s^ gene | NA | PPI | 807 |
| Rv2155c | murD | β-lactam^s^ gene | NA | Rv2981c | ddlA | β-lactam^s^ gene | NA | PPI | 981 |
| Rv2156c | murX | β-lactam^s^ gene | NA | Rv2157c | murF | β-lactam^s^ gene | NA | PPI | 999 |
| Rv2156c | murX | β-lactam^s^ gene | NA | Rv2158c | murE | β-lactam^s^ gene | NA | PPI | 998 |
| Rv2156c | murX | β-lactam^s^ gene | NA | Rv2163c | pbpB | β-lactam^s^ gene | NA | PPI | 980 |
| Rv2156c | murX | β-lactam^s^ gene | NA | Rv2864c | Rv2864c | β-lactam^s^ gene | NA | PPI | 857 |
| Rv2156c | murX | β-lactam^s^ gene | NA | Rv2911 | dacB2 | β-lactam^s^ gene | NA | PPI | 724 |
| Rv2156c | murX | β-lactam^s^ gene | NA | Rv2981c | ddlA | β-lactam^s^ gene | NA | PPI | 969 |
| Rv2156c | murX | β-lactam^s^ gene | NA | Rv3627c | Rv3627c | β-lactam^s^ gene | NA | PPI | 718 |
| Rv2156c | murX | β-lactam^s^ gene | NA | Rv3682 | ponA2 | β-lactam^s^ gene | NA | PPI | 708 |
| Rv2157c | murF | β-lactam^s^ gene | NA | Rv2158c | murE | β-lactam^s^ gene | NA | PPI | 999 |
| Rv2157c | murF | β-lactam^s^ gene | NA | Rv2163c | pbpB | β-lactam^s^ gene | NA | PPI | 921 |
| Rv2157c | murF | β-lactam^s^ gene | NA | Rv2864c | Rv2864c | β-lactam^s^ gene | NA | PPI | 761 |
| Rv2157c | murF | β-lactam^s^ gene | NA | Rv2911 | dacB2 | β-lactam^s^ gene | NA | PPI | 744 |
| Rv2157c | murF | β-lactam^s^ gene | NA | Rv2981c | ddlA | β-lactam^s^ gene | NA | PPI | 997 |
| Rv2157c | murF | β-lactam^s^ gene | NA | Rv3330 | dacB1 | β-lactam^s^ gene | NA | PPI | 755 |
| Rv2158c | murE | β-lactam^s^ gene | NA | Rv2163c | pbpB | β-lactam^s^ gene | NA | PPI | 977 |
| Rv2158c | murE | β-lactam^s^ gene | NA | Rv2726c | dapF | β-lactam^s^ gene | NA | PPI | 993 |
| Rv2158c | murE | β-lactam^s^ gene | NA | Rv2864c | Rv2864c | β-lactam^s^ gene | NA | PPI | 803 |
| Rv2158c | murE | β-lactam^s^ gene | NA | Rv2981c | ddlA | β-lactam^s^ gene | NA | PPI | 988 |
| Rv2163c | pbpB | β-lactam^s^ gene | NA | Rv2911 | dacB2 | β-lactam^s^ gene | NA | PPI | 886 |
| Rv2163c | pbpB | β-lactam^s^ gene | NA | Rv2981c | ddlA | β-lactam^s^ gene | NA | PPI | 906 |
| Rv2163c | pbpB | β-lactam^s^ gene | NA | Rv3330 | dacB1 | β-lactam^s^ gene | NA | PPI | 887 |
| Rv2163c | pbpB | β-lactam^s^ gene | NA | Rv3627c | Rv3627c | β-lactam^s^ gene | NA | PPI | 825 |
| Rv2163c | pbpB | β-lactam^s^ gene | NA | Rv3682 | ponA2 | β-lactam^s^ gene | NA | PPI | 995 |
| Rv2223c | Rv2223c | β-lactam^s^ gene | NA | Rv2256c | Rv2256c | β-lactam^s^ gene | NA | PPI | 717 |
| Rv2257c | Rv2257c | β-lactam^s^ gene | NA | Rv2258c | Rv2258c | β-lactam^s^ gene | NA | PPI | 824 |
| Rv2373c | dnaJ2 | β-lactam^s^ gene | NA | Rv2773c | dapB | β-lactam^s^ gene | NA | PPI | 717 |
| Rv2543 | lppA | β-lactam^s^ gene | NA | Rv2544 | lppB | β-lactam^s^ gene | NA | PPI | 859 |
| Rv2726c | dapF | β-lactam^s^ gene | NA | Rv2773c | dapB | β-lactam^s^ gene | NA | PPI | 948 |
| Rv2864c | Rv2864c | β-lactam^s^ gene | NA | Rv2911 | dacB2 | β-lactam^s^ gene | NA | PPI | 870 |
| Rv2864c | Rv2864c | β-lactam^s^ gene | NA | Rv2981c | ddlA | β-lactam^s^ gene | NA | PPI | 772 |
| Rv2864c | Rv2864c | β-lactam^s^ gene | NA | Rv3330 | dacB1 | β-lactam^s^ gene | NA | PPI | 870 |
| Rv2864c | Rv2864c | β-lactam^s^ gene | NA | Rv3627c | Rv3627c | β-lactam^s^ gene | NA | PPI | 819 |
| Rv2864c | Rv2864c | β-lactam^s^ gene | NA | Rv3682 | ponA2 | β-lactam^s^ gene | NA | PPI | 986 |
| Rv2911 | dacB2 | β-lactam^s^ gene | NA | Rv3682 | ponA2 | β-lactam^s^ gene | NA | PPI | 804 |
| Rv2926c | Rv2926c | β-lactam^s^ gene | NA | Rv2927c | Rv2927c | β-lactam^s^ gene | NA | PPI | 801 |
| Rv3330 | dacB1 | β-lactam^s^ gene | NA | Rv3682 | ponA2 | β-lactam^s^ gene | NA | PPI | 804 |
| Rv3627c | Rv3627c | β-lactam^s^ gene | NA | Rv3682 | ponA2 | β-lactam^s^ gene | NA | PPI | 780 |
| Rv0005 | gyrB | DR gene | FLQ | Rv0006 | gyrA | DR gene | FLQ | PPI | 999 |
| Rv0005 | gyrB | DR gene | FLQ | Rv0667 | rpoB | DR gene | RIF | PPI | 778 |
| Rv0006 | gyrA | DR gene | FLQ | Rv0667 | rpoB | DR gene | RIF | PPI | 715 |
| Rv0129c | fbpC | DR gene | INH | Rv3794 | embA | DR gene | EMB | PPI | 706 |
| Rv0129c | fbpC | DR gene | INH | Rv3795 | embB | DR gene | EMB,INH,RIF | PPI | 715 |
| Rv0341 | iniB | DR gene | EMB,INH | Rv0342 | iniA | DR gene | EMB,INH | PPI | 731 |
| Rv0341 | iniB | DR gene | EMB,INH | Rv0343 | iniC | DR gene | EMB,INH | PPI | 731 |
| Rv0342 | iniA | DR gene | EMB,INH | Rv0343 | iniC | DR gene | EMB,INH | PPI | 968 |
| Rv0667 | rpoB | DR gene | RIF | Rv0682 | rpsL | DR gene | SM | PPI | 975 |
| Rv1483 | fabG1 | DR gene | ETH,INH | Rv1484 | inhA | DR gene | ETH,INH | PPI | 958 |
| Rv1483 | fabG1 | DR gene | ETH,INH | Rv2243 | fabD | DR gene | INH | PPI | 968 |
| Rv1483 | fabG1 | DR gene | ETH,INH | Rv2245 | kasA | DR gene | INH | PPI | 997 |
| Rv1484 | inhA | DR gene | ETH,INH | Rv2243 | fabD | DR gene | INH | PPI | 816 |
| Rv1484 | inhA | DR gene | ETH,INH | Rv2245 | kasA | DR gene | INH | PPI | 989 |
| Rv1908c | katG | DR gene | INH | Rv1909c | furA | DR gene | INH | PPI | 973 |
| Rv2242 | Rv2242 | DR gene | INH | Rv2243 | fabD | DR gene | INH | PPI | 826 |
| Rv2243 | fabD | DR gene | INH | Rv2245 | kasA | DR gene | INH | PPI | 999 |
| Rv2243 | fabD | DR gene | INH | Rv2247 | accD6 | DR gene | INH | PPI | 924 |
| Rv2245 | kasA | DR gene | INH | Rv2247 | accD6 | DR gene | INH | PPI | 942 |
| Rv3264c | manB | DR gene | EMB | Rv3266c | rmlD | DR gene | EMB | PPI | 924 |
| Rv3794 | embA | DR gene | EMB | Rv3795 | embB | DR gene | EMB,INH,RIF | PPI | 884 |
| Rv0014c | pknB | β-lactam^s^ gene | NA | Rv1267c | embR | DR gene | EMB | PPI | 808 |
| Rv0112 | gca | β-lactam^s^ gene | NA | Rv3264c | manB | DR gene | EMB | PPI | 825 |
| Rv0113 | gmhA | β-lactam^s^ gene | NA | Rv3264c | manB | DR gene | EMB | PPI | 729 |
| Rv1307 | atpH | β-lactam^s^ gene | NA | Rv3919c | gid | DR gene | SM | PPI | 748 |
| Rv1503c | Rv1503c | β-lactam^s^ gene | NA | Rv3264c | manB | DR gene | EMB | PPI | 750 |
| Rv1847 | Rv1847 | β-lactam^s^ gene | NA | Rv2243 | fabD | DR gene | INH | PPI | 705 |
| Rv0005 | gyrB | DR gene | FLQ | Rv2150c | ftsZ | β-lactam^s^ gene | NA | PPI | 739 |
| Rv0006 | gyrA | DR gene | FLQ | Rv2150c | ftsZ | β-lactam^s^ gene | NA | PPI | 771 |
| Rv0667 | rpoB | DR gene | RIF | Rv1307 | atpH | β-lactam^s^ gene | NA | PPI | 733 |
| Rv0667 | rpoB | DR gene | RIF | Rv1308 | atpA | β-lactam^s^ gene | NA | PPI | 713 |
| Rv0667 | rpoB | DR gene | RIF | Rv1310 | atpD | β-lactam^s^ gene | NA | PPI | 743 |
| Rv0682 | rpsL | DR gene | SM | Rv1307 | atpH | β-lactam^s^ gene | NA | PPI | 833 |
| Rv1483 | fabG1 | DR gene | ETH,INH | Rv2926c | Rv2926c | β-lactam^s^ gene | NA | PPI | 725 |
| Rv2243 | fabD | DR gene | INH | Rv2926c | Rv2926c | β-lactam^s^ gene | NA | PPI | 784 |
| Rv3919c | gid | DR gene | SM | Rv3921c | Rv3921c | β-lactam^s^ gene | NA | PPI | 859 |
| Rv1846c | blaI | β-lactam^s^ gene | NA | Rv2069 | sigC | β-lactam^s^ gene | NA | GRN | 0.916859 |
| Rv1846c | blaI | β-lactam^s^ gene | NA | Rv3921c | Rv3921c | β-lactam^s^ gene | NA | GRN | 0.95133 |
| Rv1456c | Rv1456c | β-lactam^s^ gene | NA | Rv1846c | blaI | β-lactam^s^ gene | NA | GRN | 0.979996 |
| Rv2069 | sigC | β-lactam^s^ gene | NA | Rv3124 | moaR1 | Both | EMB | GRN | 0.820574 |
| Rv1503c | Rv1503c | β-lactam^s^ gene | NA | Rv2069 | sigC | β-lactam^s^ gene | NA | GRN | 0.752013 |
| Rv2069 | sigC | β-lactam^s^ gene | NA | Rv2307B | Rv2307B | β-lactam^s^ gene | NA | GRN | 0.789359 |
| Rv2069 | sigC | β-lactam^s^ gene | NA | Rv3823c | mmpL8 | β-lactam^s^ gene | NA | GRN | 0.738922 |
| Rv2069 | sigC | β-lactam^s^ gene | NA | Rv2145c | wag31 | β-lactam^s^ gene | NA | GRN | 0.892376 |
