# Supplementary material for: Collateral Sensitivity to β-Lactam Drugs in Drug-Resistant Tuberculosis Is Driven by the Transcriptional Wiring of BlaI Operon Genes
Source: mSphere. 2021 May 28;6(3):e00245-21. doi: 10.1128/mSphere.00245-21 (PMC8265638; doi:10.1128/mSphere.00245-21)
Supplement: TABLE S5 [file msphere.00245-21-st005.docx]

**Supplementary Table S5.**

| **Gene1** | **Name1** | **Class1** | **Gene2** | **Name2** | **Class2** | **Database** | **Direction** | **Access time** |
| --- | --- | --- | --- | --- | --- | --- | --- | --- |
| Rv1307 | atpH | DR gene | Rv3919c | gid | β-lactam^s^ gene | STRING | DR gene-> β-lactam^s^ gene | 782.989 |
| Rv0682 | rpsL | DR gene | Rv1307 | atpH | β-lactam^s^ gene | STRING | DR gene-> β-lactam^s^ gene | 918.9204 |
| Rv0667 | rpoB | DR gene | Rv1307 | atpH | β-lactam^s^ gene | STRING | DR gene-> β-lactam^s^ gene | 934.8335 |
| Rv0005 | gyrB | DR gene | Rv1307 | atpH | β-lactam^s^ gene | STRING | DR gene-> β-lactam^s^ gene | 951.1498 |
| Rv0006 | gyrA | DR gene | Rv1307 | atpH | β-lactam^s^ gene | STRING | DR gene-> β-lactam^s^ gene | 988.9233 |
| Rv1307 | atpH | DR gene | Rv1854c | ndh | β-lactam^s^ gene | STRING | DR gene-> β-lactam^s^ gene | 989.9219 |
| Rv1267c | embR | DR gene | Rv1307 | atpH | β-lactam^s^ gene | STRING | DR gene-> β-lactam^s^ gene | 1012.037 |
| Rv1307 | atpH | DR gene | Rv2764c | NA | β-lactam^s^ gene | STRING | DR gene-> β-lactam^s^ gene | 1012.095 |
| Rv1307 | atpH | DR gene | Rv1484 | inhA | β-lactam^s^ gene | STRING | DR gene-> β-lactam^s^ gene | 1012.323 |
| Rv1307 | atpH | DR gene | Rv1909c | furA | β-lactam^s^ gene | STRING | DR gene-> β-lactam^s^ gene | 1012.841 |
| Rv1307 | atpH | DR gene | Rv1694 | tlyA | β-lactam^s^ gene | STRING | DR gene-> β-lactam^s^ gene | 1016.985 |
| Rv1307 | atpH | DR gene | Rv2428 | NA | β-lactam^s^ gene | STRING | DR gene-> β-lactam^s^ gene | 1019.446 |
| Rv1307 | atpH | DR gene | Rv1908c | katG | β-lactam^s^ gene | STRING | DR gene-> β-lactam^s^ gene | 1019.505 |
| Rv1307 | atpH | DR gene | Rv2242 | Rv2242 | β-lactam^s^ gene | STRING | DR gene-> β-lactam^s^ gene | 1022.359 |
| Rv1307 | atpH | DR gene | Rv2243 | fabD | β-lactam^s^ gene | STRING | DR gene-> β-lactam^s^ gene | 1023.878 |
| Rv1307 | atpH | DR gene | Rv2245 | kasA | β-lactam^s^ gene | STRING | DR gene-> β-lactam^s^ gene | 1026.793 |
| Rv1307 | atpH | DR gene | Rv1483 | fabG1 | β-lactam^s^ gene | STRING | DR gene-> β-lactam^s^ gene | 1028.111 |
| Rv1307 | atpH | DR gene | Rv2846c | NA | β-lactam^s^ gene | STRING | DR gene-> β-lactam^s^ gene | 1034.211 |
| Rv1307 | atpH | DR gene | Rv2247 | accD6 | β-lactam^s^ gene | STRING | DR gene-> β-lactam^s^ gene | 1037.112 |
| Rv1307 | atpH | DR gene | Rv3264c | manB | β-lactam^s^ gene | STRING | DR gene-> β-lactam^s^ gene | 1038.807 |
| Rv1307 | atpH | DR gene | Rv3266c | rmlD | β-lactam^s^ gene | STRING | DR gene-> β-lactam^s^ gene | 1044.875 |
| Rv0343 | iniC | DR gene | Rv1307 | atpH | β-lactam^s^ gene | STRING | DR gene-> β-lactam^s^ gene | 1049.673 |
| Rv0342 | iniA | DR gene | Rv1307 | atpH | β-lactam^s^ gene | STRING | DR gene-> β-lactam^s^ gene | 1049.673 |
| Rv1307 | atpH | DR gene | Rv2043c | NA | β-lactam^s^ gene | STRING | DR gene-> β-lactam^s^ gene | 1049.749 |
| Rv1307 | atpH | DR gene | Rv3139 | NA | β-lactam^s^ gene | STRING | DR gene-> β-lactam^s^ gene | 1051.213 |
| Rv0341 | iniB | DR gene | Rv1307 | atpH | β-lactam^s^ gene | STRING | DR gene-> β-lactam^s^ gene | 1051.673 |
| Rv0129c | fbpC | DR gene | Rv1307 | atpH | β-lactam^s^ gene | STRING | DR gene-> β-lactam^s^ gene | 1053.668 |
| Rv1307 | atpH | DR gene | Rv3794 | embA | β-lactam^s^ gene | STRING | DR gene-> β-lactam^s^ gene | 1054.185 |
| Rv1307 | atpH | DR gene | Rv3793 | NA | β-lactam^s^ gene | STRING | DR gene-> β-lactam^s^ gene | 1054.556 |
| Rv1307 | atpH | DR gene | Rv3795 | embB | β-lactam^s^ gene | STRING | DR gene-> β-lactam^s^ gene | 1054.738 |
| Rv0667 | rpoB | β-lactam^s^ gene | Rv1308 | atpA | DR gene | STRING | β-lactam^s^ gene ->DR gene | 1074.369 |
| Rv0667 | rpoB | β-lactam^s^ gene | Rv1310 | atpD | DR gene | STRING | β-lactam^s^ gene ->DR gene | 1088.876 |
| Rv0667 | rpoB | β-lactam^s^ gene | Rv1306 | atpF | DR gene | STRING | β-lactam^s^ gene ->DR gene | 1097.419 |
| Rv0667 | rpoB | β-lactam^s^ gene | Rv1307 | atpH | DR gene | STRING | β-lactam^s^ gene ->DR gene | 1098.284 |
| Rv0667 | rpoB | β-lactam^s^ gene | Rv1305 | atpE | DR gene | STRING | β-lactam^s^ gene ->DR gene | 1115.492 |
| Rv0682 | rpsL | β-lactam^s^ gene | Rv1308 | atpA | DR gene | STRING | β-lactam^s^ gene ->DR gene | 1126.762 |
| Rv0682 | rpsL | β-lactam^s^ gene | Rv1307 | atpH | DR gene | STRING | β-lactam^s^ gene ->DR gene | 1127.577 |
| Rv0667 | rpoB | β-lactam^s^ gene | Rv1009 | NA | DR gene | STRING | β-lactam^s^ gene ->DR gene | 1128.202 |
| Rv0667 | rpoB | β-lactam^s^ gene | Rv3921c | Rv3921c | DR gene | STRING | β-lactam^s^ gene ->DR gene | 1128.68 |
| Rv0682 | rpsL | β-lactam^s^ gene | Rv1306 | atpF | DR gene | STRING | β-lactam^s^ gene ->DR gene | 1129.073 |
| Rv0667 | rpoB | β-lactam^s^ gene | Rv1309 | atpG | DR gene | STRING | β-lactam^s^ gene ->DR gene | 1130.261 |
| Rv0667 | rpoB | β-lactam^s^ gene | Rv1303 | Rv1303 | DR gene | STRING | β-lactam^s^ gene ->DR gene | 1132.188 |
| Rv0667 | rpoB | β-lactam^s^ gene | Rv1312 | Rv1312 | DR gene | STRING | β-lactam^s^ gene ->DR gene | 1133.429 |
| Rv0667 | rpoB | β-lactam^s^ gene | Rv2943 | NA | DR gene | STRING | β-lactam^s^ gene ->DR gene | 1143.521 |
| Rv0667 | rpoB | β-lactam^s^ gene | Rv2373c | dnaJ2 | DR gene | STRING | β-lactam^s^ gene ->DR gene | 1144.636 |
| Rv0667 | rpoB | β-lactam^s^ gene | Rv1304 | atpB | DR gene | STRING | β-lactam^s^ gene ->DR gene | 1144.884 |
| Rv0667 | rpoB | β-lactam^s^ gene | Rv1311 | atpC | DR gene | STRING | β-lactam^s^ gene ->DR gene | 1148.364 |
| Rv0682 | rpsL | β-lactam^s^ gene | Rv1009 | NA | DR gene | STRING | β-lactam^s^ gene ->DR gene | 1154.99 |
| Rv0682 | rpsL | β-lactam^s^ gene | Rv1310 | atpD | DR gene | STRING | β-lactam^s^ gene ->DR gene | 1157.67 |
| Rv0682 | rpsL | β-lactam^s^ gene | Rv1305 | atpE | DR gene | STRING | β-lactam^s^ gene ->DR gene | 1160.934 |
| Rv0667 | rpoB | β-lactam^s^ gene | Rv2773c | dapB | DR gene | STRING | β-lactam^s^ gene ->DR gene | 1161 |
| Rv0667 | rpoB | β-lactam^s^ gene | Rv2150c | ftsZ | DR gene | STRING | β-lactam^s^ gene ->DR gene | 1162.188 |
| Rv0667 | rpoB | β-lactam^s^ gene | Rv2093c | tatC | DR gene | STRING | β-lactam^s^ gene ->DR gene | 1163.376 |
| Rv0015c | pknA | β-lactam^s^ gene | Rv0667 | rpoB | DR gene | STRING | β-lactam^s^ gene ->DR gene | 1165.144 |
| Rv0014c | pknB | β-lactam^s^ gene | Rv0667 | rpoB | DR gene | STRING | β-lactam^s^ gene ->DR gene | 1167.279 |
| Rv0667 | rpoB | β-lactam^s^ gene | Rv2145c | wag31 | DR gene | STRING | β-lactam^s^ gene ->DR gene | 1167.89 |
| Rv0682 | rpsL | β-lactam^s^ gene | Rv1309 | atpG | DR gene | STRING | β-lactam^s^ gene ->DR gene | 1168.333 |
| Rv0667 | rpoB | β-lactam^s^ gene | Rv2926c | Rv2926c | DR gene | STRING | β-lactam^s^ gene ->DR gene | 1169.313 |
| Rv0667 | rpoB | β-lactam^s^ gene | Rv1433 | NA | DR gene | STRING | β-lactam^s^ gene ->DR gene | 1171.626 |
| Rv0682 | rpsL | β-lactam^s^ gene | Rv3921c | Rv3921c | DR gene | STRING | β-lactam^s^ gene ->DR gene | 1171.868 |
| Rv0113 | gmhA | β-lactam^s^ gene | Rv0667 | rpoB | DR gene | STRING | β-lactam^s^ gene ->DR gene | 1172.902 |
| Rv0667 | rpoB | β-lactam^s^ gene | Rv1024 | Rv1024 | DR gene | STRING | β-lactam^s^ gene ->DR gene | 1173.752 |
| Rv0667 | rpoB | β-lactam^s^ gene | Rv2147c | Rv2147c | DR gene | STRING | β-lactam^s^ gene ->DR gene | 1173.938 |
| Rv0667 | rpoB | β-lactam^s^ gene | Rv2730 | NA | DR gene | STRING | β-lactam^s^ gene ->DR gene | 1174.631 |
| Rv0667 | rpoB | β-lactam^s^ gene | Rv2151c | ftsQ | DR gene | STRING | β-lactam^s^ gene ->DR gene | 1175.368 |
| Rv0682 | rpsL | β-lactam^s^ gene | Rv1303 | Rv1303 | DR gene | STRING | β-lactam^s^ gene ->DR gene | 1177.12 |
| Rv0667 | rpoB | β-lactam^s^ gene | Rv2726c | dapF | DR gene | STRING | β-lactam^s^ gene ->DR gene | 1177.513 |
| Rv0667 | rpoB | β-lactam^s^ gene | Rv3627c | Rv3627c | DR gene | STRING | β-lactam^s^ gene ->DR gene | 1177.685 |
| Rv0667 | rpoB | β-lactam^s^ gene | Rv1025 | Rv1025 | DR gene | STRING | β-lactam^s^ gene ->DR gene | 1179.55 |
| Rv0116c | ldtA | β-lactam^s^ gene | Rv0667 | rpoB | DR gene | STRING | β-lactam^s^ gene ->DR gene | 1180.072 |
| Rv0667 | rpoB | β-lactam^s^ gene | Rv3682 | ponA2 | DR gene | STRING | β-lactam^s^ gene ->DR gene | 1182.805 |
| Rv0667 | rpoB | β-lactam^s^ gene | Rv2154c | ftsW | DR gene | STRING | β-lactam^s^ gene ->DR gene | 1182.909 |
| Rv0667 | rpoB | β-lactam^s^ gene | Rv2752c | NA | DR gene | STRING | β-lactam^s^ gene ->DR gene | 1183.083 |
| Rv0667 | rpoB | β-lactam^s^ gene | Rv2256c | Rv2256c | DR gene | STRING | β-lactam^s^ gene ->DR gene | 1183.16 |
| Rv0667 | rpoB | β-lactam^s^ gene | Rv2069 | sigC | DR gene | STRING | β-lactam^s^ gene ->DR gene | 1183.18 |
| Rv0667 | rpoB | β-lactam^s^ gene | Rv2155c | murD | DR gene | STRING | β-lactam^s^ gene ->DR gene | 1184.218 |
| Rv0667 | rpoB | β-lactam^s^ gene | Rv2156c | murX | DR gene | STRING | β-lactam^s^ gene ->DR gene | 1184.259 |
| Rv0667 | rpoB | β-lactam^s^ gene | Rv2864c | Rv2864c | DR gene | STRING | β-lactam^s^ gene ->DR gene | 1184.306 |
| Rv0667 | rpoB | β-lactam^s^ gene | Rv2158c | murE | DR gene | STRING | β-lactam^s^ gene ->DR gene | 1184.806 |
| Rv0667 | rpoB | β-lactam^s^ gene | Rv2223c | Rv2223c | DR gene | STRING | β-lactam^s^ gene ->DR gene | 1185.16 |
| Rv0050 | ponA1 | β-lactam^s^ gene | Rv0667 | rpoB | DR gene | STRING | β-lactam^s^ gene ->DR gene | 1185.659 |
| Rv0667 | rpoB | β-lactam^s^ gene | Rv2163c | pbpB | DR gene | STRING | β-lactam^s^ gene ->DR gene | 1185.666 |
| Rv0667 | rpoB | β-lactam^s^ gene | Rv2152c | murC | DR gene | STRING | β-lactam^s^ gene ->DR gene | 1185.743 |
| Rv0667 | rpoB | β-lactam^s^ gene | Rv2981c | ddlA | DR gene | STRING | β-lactam^s^ gene ->DR gene | 1185.8 |
| Rv0667 | rpoB | β-lactam^s^ gene | Rv2157c | murF | DR gene | STRING | β-lactam^s^ gene ->DR gene | 1186.141 |
| Rv0667 | rpoB | β-lactam^s^ gene | Rv3727 | NA | DR gene | STRING | β-lactam^s^ gene ->DR gene | 1186.229 |
| Rv0682 | rpsL | β-lactam^s^ gene | Rv1312 | Rv1312 | DR gene | STRING | β-lactam^s^ gene ->DR gene | 1186.739 |
| Rv0667 | rpoB | β-lactam^s^ gene | Rv2911 | dacB2 | DR gene | STRING | β-lactam^s^ gene ->DR gene | 1187.697 |
| Rv0667 | rpoB | β-lactam^s^ gene | Rv3330 | dacB1 | DR gene | STRING | β-lactam^s^ gene ->DR gene | 1187.841 |
| Rv0667 | rpoB | β-lactam^s^ gene | Rv3677c | NA | DR gene | STRING | β-lactam^s^ gene ->DR gene | 1188.077 |
| Rv0667 | rpoB | β-lactam^s^ gene | Rv1846c | blaI | DR gene | STRING | β-lactam^s^ gene ->DR gene | 1188.232 |
| Rv0667 | rpoB | β-lactam^s^ gene | Rv2094c | tatA | DR gene | STRING | β-lactam^s^ gene ->DR gene | 1188.472 |
| Rv0011c | Rv0011c | β-lactam^s^ gene | Rv0667 | rpoB | DR gene | STRING | β-lactam^s^ gene ->DR gene | 1188.948 |
| Rv0682 | rpsL | β-lactam^s^ gene | Rv1304 | atpB | DR gene | STRING | β-lactam^s^ gene ->DR gene | 1189.307 |
| Rv0667 | rpoB | β-lactam^s^ gene | Rv1338 | murI | DR gene | STRING | β-lactam^s^ gene ->DR gene | 1189.656 |
| Rv0406c | Rv0406c | β-lactam^s^ gene | Rv0667 | rpoB | DR gene | STRING | β-lactam^s^ gene ->DR gene | 1190.276 |
| Rv0667 | rpoB | β-lactam^s^ gene | Rv0849 | NA | DR gene | STRING | β-lactam^s^ gene ->DR gene | 1191.57 |
| Rv0667 | rpoB | β-lactam^s^ gene | Rv2518c | NA | DR gene | STRING | β-lactam^s^ gene ->DR gene | 1192.298 |
| Rv0192 | Rv0192 | β-lactam^s^ gene | Rv0667 | rpoB | DR gene | STRING | β-lactam^s^ gene ->DR gene | 1193.143 |
| Rv0667 | rpoB | β-lactam^s^ gene | Rv1456c | Rv1456c | DR gene | STRING | β-lactam^s^ gene ->DR gene | 1193.219 |
| Rv0682 | rpsL | β-lactam^s^ gene | Rv1311 | atpC | DR gene | STRING | β-lactam^s^ gene ->DR gene | 1193.555 |
| Rv0194 | Rv0194 | β-lactam^s^ gene | Rv0667 | rpoB | DR gene | STRING | β-lactam^s^ gene ->DR gene | 1193.884 |
| Rv0667 | rpoB | β-lactam^s^ gene | Rv1847 | Rv1847 | DR gene | STRING | β-lactam^s^ gene ->DR gene | 1195.624 |
| Rv0667 | rpoB | β-lactam^s^ gene | Rv2927c | Rv2927c | DR gene | STRING | β-lactam^s^ gene ->DR gene | 1201.391 |
| Rv0667 | rpoB | β-lactam^s^ gene | Rv3107c | NA | DR gene | STRING | β-lactam^s^ gene ->DR gene | 1202.894 |
| Rv0112 | gca | β-lactam^s^ gene | Rv0667 | rpoB | DR gene | STRING | β-lactam^s^ gene ->DR gene | 1203.178 |
| Rv0682 | rpsL | β-lactam^s^ gene | Rv2373c | dnaJ2 | DR gene | STRING | β-lactam^s^ gene ->DR gene | 1204.707 |
| Rv0682 | rpsL | β-lactam^s^ gene | Rv2943 | NA | DR gene | STRING | β-lactam^s^ gene ->DR gene | 1205.062 |
| Rv0667 | rpoB | β-lactam^s^ gene | Rv2509 | NA | DR gene | STRING | β-lactam^s^ gene ->DR gene | 1206.624 |
| Rv0682 | rpsL | β-lactam^s^ gene | Rv2093c | tatC | DR gene | STRING | β-lactam^s^ gene ->DR gene | 1207.706 |
| Rv0015c | pknA | β-lactam^s^ gene | Rv0682 | rpsL | DR gene | STRING | β-lactam^s^ gene ->DR gene | 1208.938 |
| Rv0682 | rpsL | β-lactam^s^ gene | Rv2773c | dapB | DR gene | STRING | β-lactam^s^ gene ->DR gene | 1209.6 |
| Rv0682 | rpsL | β-lactam^s^ gene | Rv2926c | Rv2926c | DR gene | STRING | β-lactam^s^ gene ->DR gene | 1210.407 |
| Rv0667 | rpoB | β-lactam^s^ gene | Rv1503c | Rv1503c | DR gene | STRING | β-lactam^s^ gene ->DR gene | 1210.443 |
| Rv0682 | rpsL | β-lactam^s^ gene | Rv2150c | ftsZ | DR gene | STRING | β-lactam^s^ gene ->DR gene | 1210.639 |
| Rv0113 | gmhA | β-lactam^s^ gene | Rv0682 | rpsL | DR gene | STRING | β-lactam^s^ gene ->DR gene | 1212.885 |
| Rv0667 | rpoB | β-lactam^s^ gene | Rv1884c | NA | DR gene | STRING | β-lactam^s^ gene ->DR gene | 1212.97 |
| Rv0014c | pknB | β-lactam^s^ gene | Rv0682 | rpsL | DR gene | STRING | β-lactam^s^ gene ->DR gene | 1213.229 |
| Rv0667 | rpoB | β-lactam^s^ gene | Rv1218c | NA | DR gene | STRING | β-lactam^s^ gene ->DR gene | 1213.23 |
| Rv0682 | rpsL | β-lactam^s^ gene | Rv2145c | wag31 | DR gene | STRING | β-lactam^s^ gene ->DR gene | 1215.455 |
| Rv0667 | rpoB | β-lactam^s^ gene | Rv3820c | NA | DR gene | STRING | β-lactam^s^ gene ->DR gene | 1216.939 |
| Rv0667 | rpoB | β-lactam^s^ gene | Rv1258c | NA | DR gene | STRING | β-lactam^s^ gene ->DR gene | 1216.97 |
| Rv0667 | rpoB | β-lactam^s^ gene | Rv1521 | NA | DR gene | STRING | β-lactam^s^ gene ->DR gene | 1218.548 |
| Rv0667 | rpoB | β-lactam^s^ gene | Rv3826 | NA | DR gene | STRING | β-lactam^s^ gene ->DR gene | 1218.548 |
| Rv0667 | rpoB | β-lactam^s^ gene | Rv2844 | NA | DR gene | STRING | β-lactam^s^ gene ->DR gene | 1219.321 |
| Rv0667 | rpoB | β-lactam^s^ gene | Rv3635 | NA | DR gene | STRING | β-lactam^s^ gene ->DR gene | 1219.98 |
| Rv0682 | rpsL | β-lactam^s^ gene | Rv2147c | Rv2147c | DR gene | STRING | β-lactam^s^ gene ->DR gene | 1220.413 |
| Rv0682 | rpsL | β-lactam^s^ gene | Rv2151c | ftsQ | DR gene | STRING | β-lactam^s^ gene ->DR gene | 1221.137 |
| Rv0682 | rpsL | β-lactam^s^ gene | Rv2726c | dapF | DR gene | STRING | β-lactam^s^ gene ->DR gene | 1222.351 |
| Rv0682 | rpsL | β-lactam^s^ gene | Rv1433 | NA | DR gene | STRING | β-lactam^s^ gene ->DR gene | 1223.099 |
| Rv0667 | rpoB | β-lactam^s^ gene | Rv3818 | NA | DR gene | STRING | β-lactam^s^ gene ->DR gene | 1223.853 |
| Rv0682 | rpsL | β-lactam^s^ gene | Rv3627c | Rv3627c | DR gene | STRING | β-lactam^s^ gene ->DR gene | 1224.058 |
| Rv0667 | rpoB | β-lactam^s^ gene | Rv2721c | NA | DR gene | STRING | β-lactam^s^ gene ->DR gene | 1226.357 |
| Rv0682 | rpsL | β-lactam^s^ gene | Rv2154c | ftsW | DR gene | STRING | β-lactam^s^ gene ->DR gene | 1228.66 |
| Rv0682 | rpsL | β-lactam^s^ gene | Rv2155c | murD | DR gene | STRING | β-lactam^s^ gene ->DR gene | 1230.03 |
| Rv0682 | rpsL | β-lactam^s^ gene | Rv2864c | Rv2864c | DR gene | STRING | β-lactam^s^ gene ->DR gene | 1230.193 |
| Rv0682 | rpsL | β-lactam^s^ gene | Rv2156c | murX | DR gene | STRING | β-lactam^s^ gene ->DR gene | 1230.194 |
| Rv0030 | Rv0030 | β-lactam^s^ gene | Rv0667 | rpoB | DR gene | STRING | β-lactam^s^ gene ->DR gene | 1230.197 |
| Rv0682 | rpsL | β-lactam^s^ gene | Rv3682 | ponA2 | DR gene | STRING | β-lactam^s^ gene ->DR gene | 1230.249 |
| Rv0682 | rpsL | β-lactam^s^ gene | Rv2158c | murE | DR gene | STRING | β-lactam^s^ gene ->DR gene | 1230.302 |
| Rv0682 | rpsL | β-lactam^s^ gene | Rv1024 | Rv1024 | DR gene | STRING | β-lactam^s^ gene ->DR gene | 1230.338 |
| Rv0682 | rpsL | β-lactam^s^ gene | Rv2152c | murC | DR gene | STRING | β-lactam^s^ gene ->DR gene | 1231.277 |
| Rv0682 | rpsL | β-lactam^s^ gene | Rv2730 | NA | DR gene | STRING | β-lactam^s^ gene ->DR gene | 1231.57 |
| Rv0682 | rpsL | β-lactam^s^ gene | Rv2981c | ddlA | DR gene | STRING | β-lactam^s^ gene ->DR gene | 1231.629 |
| Rv0682 | rpsL | β-lactam^s^ gene | Rv2163c | pbpB | DR gene | STRING | β-lactam^s^ gene ->DR gene | 1231.761 |
| Rv0682 | rpsL | β-lactam^s^ gene | Rv2157c | murF | DR gene | STRING | β-lactam^s^ gene ->DR gene | 1231.865 |
| Rv0682 | rpsL | β-lactam^s^ gene | Rv2752c | NA | DR gene | STRING | β-lactam^s^ gene ->DR gene | 1232.395 |
| Rv0050 | ponA1 | β-lactam^s^ gene | Rv0682 | rpsL | DR gene | STRING | β-lactam^s^ gene ->DR gene | 1233.03 |
| Rv0116c | ldtA | β-lactam^s^ gene | Rv0682 | rpsL | DR gene | STRING | β-lactam^s^ gene ->DR gene | 1233.07 |
| Rv0682 | rpsL | β-lactam^s^ gene | Rv3727 | NA | DR gene | STRING | β-lactam^s^ gene ->DR gene | 1233.951 |
| Rv0682 | rpsL | β-lactam^s^ gene | Rv2911 | dacB2 | DR gene | STRING | β-lactam^s^ gene ->DR gene | 1234.441 |
| Rv0682 | rpsL | β-lactam^s^ gene | Rv3330 | dacB1 | DR gene | STRING | β-lactam^s^ gene ->DR gene | 1234.666 |
| Rv0682 | rpsL | β-lactam^s^ gene | Rv2256c | Rv2256c | DR gene | STRING | β-lactam^s^ gene ->DR gene | 1234.863 |
| Rv0682 | rpsL | β-lactam^s^ gene | Rv1025 | Rv1025 | DR gene | STRING | β-lactam^s^ gene ->DR gene | 1235.013 |
| Rv0682 | rpsL | β-lactam^s^ gene | Rv2094c | tatA | DR gene | STRING | β-lactam^s^ gene ->DR gene | 1235.22 |
| Rv0667 | rpoB | β-lactam^s^ gene | Rv3065 | NA | DR gene | STRING | β-lactam^s^ gene ->DR gene | 1236.784 |
| Rv0682 | rpsL | β-lactam^s^ gene | Rv2223c | Rv2223c | DR gene | STRING | β-lactam^s^ gene ->DR gene | 1236.863 |
| Rv0011c | Rv0011c | β-lactam^s^ gene | Rv0682 | rpsL | DR gene | STRING | β-lactam^s^ gene ->DR gene | 1237.763 |
| Rv0112 | gca | β-lactam^s^ gene | Rv0682 | rpsL | DR gene | STRING | β-lactam^s^ gene ->DR gene | 1238.333 |
| Rv0682 | rpsL | β-lactam^s^ gene | Rv1846c | blaI | DR gene | STRING | β-lactam^s^ gene ->DR gene | 1238.763 |
| Rv0682 | rpsL | β-lactam^s^ gene | Rv1338 | murI | DR gene | STRING | β-lactam^s^ gene ->DR gene | 1239.808 |
| Rv0682 | rpsL | β-lactam^s^ gene | Rv2069 | sigC | DR gene | STRING | β-lactam^s^ gene ->DR gene | 1240.586 |
| Rv0194 | Rv0194 | β-lactam^s^ gene | Rv0682 | rpsL | DR gene | STRING | β-lactam^s^ gene ->DR gene | 1241.069 |
| Rv0682 | rpsL | β-lactam^s^ gene | Rv1456c | Rv1456c | DR gene | STRING | β-lactam^s^ gene ->DR gene | 1242.194 |
| Rv0682 | rpsL | β-lactam^s^ gene | Rv0849 | NA | DR gene | STRING | β-lactam^s^ gene ->DR gene | 1242.466 |
| Rv0192 | Rv0192 | β-lactam^s^ gene | Rv0682 | rpsL | DR gene | STRING | β-lactam^s^ gene ->DR gene | 1243.56 |
| Rv0682 | rpsL | β-lactam^s^ gene | Rv2518c | NA | DR gene | STRING | β-lactam^s^ gene ->DR gene | 1244.391 |
| Rv0682 | rpsL | β-lactam^s^ gene | Rv1503c | Rv1503c | DR gene | STRING | β-lactam^s^ gene ->DR gene | 1246.464 |
| Rv0682 | rpsL | β-lactam^s^ gene | Rv1847 | Rv1847 | DR gene | STRING | β-lactam^s^ gene ->DR gene | 1247.581 |
| Rv0682 | rpsL | β-lactam^s^ gene | Rv3677c | NA | DR gene | STRING | β-lactam^s^ gene ->DR gene | 1247.895 |
| Rv0682 | rpsL | β-lactam^s^ gene | Rv2927c | Rv2927c | DR gene | STRING | β-lactam^s^ gene ->DR gene | 1249.493 |
| Rv0682 | rpsL | β-lactam^s^ gene | Rv3107c | NA | DR gene | STRING | β-lactam^s^ gene ->DR gene | 1250.06 |
| Rv0406c | Rv0406c | β-lactam^s^ gene | Rv0682 | rpsL | DR gene | STRING | β-lactam^s^ gene ->DR gene | 1250.346 |
| Rv0667 | rpoB | β-lactam^s^ gene | Rv1665 | NA | DR gene | STRING | β-lactam^s^ gene ->DR gene | 1254.283 |
| Rv0682 | rpsL | β-lactam^s^ gene | Rv2509 | NA | DR gene | STRING | β-lactam^s^ gene ->DR gene | 1255.991 |
| Rv0682 | rpsL | β-lactam^s^ gene | Rv1884c | NA | DR gene | STRING | β-lactam^s^ gene ->DR gene | 1261.96 |
| Rv0682 | rpsL | β-lactam^s^ gene | Rv1218c | NA | DR gene | STRING | β-lactam^s^ gene ->DR gene | 1262.145 |
| Rv0682 | rpsL | β-lactam^s^ gene | Rv3820c | NA | DR gene | STRING | β-lactam^s^ gene ->DR gene | 1266.603 |
| Rv0682 | rpsL | β-lactam^s^ gene | Rv1258c | NA | DR gene | STRING | β-lactam^s^ gene ->DR gene | 1266.639 |
| Rv0682 | rpsL | β-lactam^s^ gene | Rv1521 | NA | DR gene | STRING | β-lactam^s^ gene ->DR gene | 1267.863 |
| Rv0682 | rpsL | β-lactam^s^ gene | Rv3826 | NA | DR gene | STRING | β-lactam^s^ gene ->DR gene | 1267.863 |
| Rv0682 | rpsL | β-lactam^s^ gene | Rv3635 | NA | DR gene | STRING | β-lactam^s^ gene ->DR gene | 1268.87 |
| Rv0682 | rpsL | β-lactam^s^ gene | Rv2844 | NA | DR gene | STRING | β-lactam^s^ gene ->DR gene | 1269.026 |
| Rv0682 | rpsL | β-lactam^s^ gene | Rv3818 | NA | DR gene | STRING | β-lactam^s^ gene ->DR gene | 1273.629 |
| Rv0682 | rpsL | β-lactam^s^ gene | Rv2721c | NA | DR gene | STRING | β-lactam^s^ gene ->DR gene | 1275.215 |
| Rv0030 | Rv0030 | β-lactam^s^ gene | Rv0682 | rpsL | DR gene | STRING | β-lactam^s^ gene ->DR gene | 1281.262 |
| Rv0682 | rpsL | β-lactam^s^ gene | Rv3065 | NA | DR gene | STRING | β-lactam^s^ gene ->DR gene | 1285.983 |
| Rv0667 | rpoB | β-lactam^s^ gene | Rv2389c | NA | DR gene | STRING | β-lactam^s^ gene ->DR gene | 1291.89 |
| Rv0682 | rpsL | β-lactam^s^ gene | Rv1665 | NA | DR gene | STRING | β-lactam^s^ gene ->DR gene | 1303.67 |
| Rv0682 | rpsL | β-lactam^s^ gene | Rv2389c | NA | DR gene | STRING | β-lactam^s^ gene ->DR gene | 1336.582 |
| Rv2069 | sigC | DR gene | Rv3124 | moaR1 | β-lactam^s^ gene | GRN | DR gene-> β-lactam^s^ gene | 1613.121 |
| Rv0682 | rpsL | DR gene | Rv2069 | sigC | β-lactam^s^ gene | GRN | DR gene-> β-lactam^s^ gene | 1703.401 |
| Rv1484 | inhA | DR gene | Rv2069 | sigC | β-lactam^s^ gene | GRN | DR gene-> β-lactam^s^ gene | 1704.29 |
| Rv0667 | rpoB | DR gene | Rv2069 | sigC | β-lactam^s^ gene | GRN | DR gene-> β-lactam^s^ gene | 1705.097 |
| Rv2069 | sigC | DR gene | Rv3139 | NA | β-lactam^s^ gene | GRN | DR gene-> β-lactam^s^ gene | 1706.246 |
| Rv2069 | sigC | DR gene | Rv2243 | fabD | β-lactam^s^ gene | GRN | DR gene-> β-lactam^s^ gene | 1706.549 |
| Rv2069 | sigC | DR gene | Rv2764c | NA | β-lactam^s^ gene | GRN | DR gene-> β-lactam^s^ gene | 1707.039 |
| Rv0341 | iniB | DR gene | Rv2069 | sigC | β-lactam^s^ gene | GRN | DR gene-> β-lactam^s^ gene | 1707.53 |
| Rv2069 | sigC | DR gene | Rv2428 | NA | β-lactam^s^ gene | GRN | DR gene-> β-lactam^s^ gene | 1707.697 |
| Rv2069 | sigC | DR gene | Rv3125c | NA | β-lactam^s^ gene | GRN | DR gene-> β-lactam^s^ gene | 1708.966 |
| Rv0006 | gyrA | DR gene | Rv2069 | sigC | β-lactam^s^ gene | GRN | DR gene-> β-lactam^s^ gene | 1709.212 |
| Rv1772 | Rv1772 | DR gene | Rv2069 | sigC | β-lactam^s^ gene | GRN | DR gene-> β-lactam^s^ gene | 1709.332 |
| Rv2069 | sigC | DR gene | Rv3854c | NA | β-lactam^s^ gene | GRN | DR gene-> β-lactam^s^ gene | 1709.415 |
| Rv2069 | sigC | DR gene | Rv2242 | Rv2242 | β-lactam^s^ gene | GRN | DR gene-> β-lactam^s^ gene | 1709.51 |
| Rv1483 | fabG1 | DR gene | Rv2069 | sigC | β-lactam^s^ gene | GRN | DR gene-> β-lactam^s^ gene | 1709.69 |
| Rv1908c | katG | DR gene | Rv2069 | sigC | β-lactam^s^ gene | GRN | DR gene-> β-lactam^s^ gene | 1709.753 |
| Rv0005 | gyrB | DR gene | Rv2069 | sigC | β-lactam^s^ gene | GRN | DR gene-> β-lactam^s^ gene | 1709.847 |
| Rv2069 | sigC | DR gene | Rv2846c | NA | β-lactam^s^ gene | GRN | DR gene-> β-lactam^s^ gene | 1710.026 |
| Rv1854c | ndh | DR gene | Rv2069 | sigC | β-lactam^s^ gene | GRN | DR gene-> β-lactam^s^ gene | 1710.134 |
| Rv2069 | sigC | DR gene | Rv3264c | manB | β-lactam^s^ gene | GRN | DR gene-> β-lactam^s^ gene | 1710.187 |
| Rv0343 | iniC | DR gene | Rv2069 | sigC | β-lactam^s^ gene | GRN | DR gene-> β-lactam^s^ gene | 1710.417 |
| Rv0342 | iniA | DR gene | Rv2069 | sigC | β-lactam^s^ gene | GRN | DR gene-> β-lactam^s^ gene | 1710.519 |
| Rv1909c | furA | DR gene | Rv2069 | sigC | β-lactam^s^ gene | GRN | DR gene-> β-lactam^s^ gene | 1710.533 |
| Rv2069 | sigC | DR gene | Rv3919c | gid | β-lactam^s^ gene | GRN | DR gene-> β-lactam^s^ gene | 1710.604 |
| Rv2069 | sigC | DR gene | Rv3794 | embA | β-lactam^s^ gene | GRN | DR gene-> β-lactam^s^ gene | 1710.624 |
| Rv1592c | Rv1592c | DR gene | Rv2069 | sigC | β-lactam^s^ gene | GRN | DR gene-> β-lactam^s^ gene | 1710.815 |
| Rv0129c | fbpC | DR gene | Rv2069 | sigC | β-lactam^s^ gene | GRN | DR gene-> β-lactam^s^ gene | 1711.024 |
| Rv2069 | sigC | DR gene | Rv3795 | embB | β-lactam^s^ gene | GRN | DR gene-> β-lactam^s^ gene | 1711.21 |
| Rv2069 | sigC | DR gene | Rv2245 | kasA | β-lactam^s^ gene | GRN | DR gene-> β-lactam^s^ gene | 1711.643 |
| Rv2069 | sigC | DR gene | Rv3793 | NA | β-lactam^s^ gene | GRN | DR gene-> β-lactam^s^ gene | 1712.262 |
| Rv1694 | tlyA | DR gene | Rv2069 | sigC | β-lactam^s^ gene | GRN | DR gene-> β-lactam^s^ gene | 1712.456 |
| Rv2069 | sigC | DR gene | Rv3266c | rmlD | β-lactam^s^ gene | GRN | DR gene-> β-lactam^s^ gene | 1712.538 |
| Rv2069 | sigC | DR gene | Rv2247 | accD6 | β-lactam^s^ gene | GRN | DR gene-> β-lactam^s^ gene | 1712.645 |
| Rv1267c | embR | DR gene | Rv2069 | sigC | β-lactam^s^ gene | GRN | DR gene-> β-lactam^s^ gene | 1712.996 |
| Rv0340 | Rv0340 | DR gene | Rv2069 | sigC | β-lactam^s^ gene | GRN | DR gene-> β-lactam^s^ gene | 1713.372 |
| Rv0667 | rpoB | β-lactam^s^ gene | Rv2160c | NA | DR gene | GRN | β-lactam^s^ gene ->DR gene | 2718.248 |
| Rv0667 | rpoB | β-lactam^s^ gene | Rv1665 | NA | DR gene | GRN | β-lactam^s^ gene ->DR gene | 2724.411 |
| Rv0667 | rpoB | β-lactam^s^ gene | Rv2307B | Rv2307B | DR gene | GRN | β-lactam^s^ gene ->DR gene | 2725.361 |
| Rv0667 | rpoB | β-lactam^s^ gene | Rv2145c | wag31 | DR gene | GRN | β-lactam^s^ gene ->DR gene | 2728.283 |
| Rv0667 | rpoB | β-lactam^s^ gene | Rv2981c | ddlA | DR gene | GRN | β-lactam^s^ gene ->DR gene | 2729.581 |
| Rv0667 | rpoB | β-lactam^s^ gene | Rv2543 | lppA | DR gene | GRN | β-lactam^s^ gene ->DR gene | 2730.304 |
| Rv0667 | rpoB | β-lactam^s^ gene | Rv1753c | NA | DR gene | GRN | β-lactam^s^ gene ->DR gene | 2730.614 |
| Rv0667 | rpoB | β-lactam^s^ gene | Rv2544 | lppB | DR gene | GRN | β-lactam^s^ gene ->DR gene | 2730.618 |
| Rv0667 | rpoB | β-lactam^s^ gene | Rv2911 | dacB2 | DR gene | GRN | β-lactam^s^ gene ->DR gene | 2730.644 |
| Rv0667 | rpoB | β-lactam^s^ gene | Rv0755c | NA | DR gene | GRN | β-lactam^s^ gene ->DR gene | 2730.716 |
| Rv0667 | rpoB | β-lactam^s^ gene | Rv1303 | Rv1303 | DR gene | GRN | β-lactam^s^ gene ->DR gene | 2731.037 |
| Rv0667 | rpoB | β-lactam^s^ gene | Rv2389c | NA | DR gene | GRN | β-lactam^s^ gene ->DR gene | 2731.662 |
| Rv0667 | rpoB | β-lactam^s^ gene | Rv2069 | sigC | DR gene | GRN | β-lactam^s^ gene ->DR gene | 2732.094 |
| Rv0667 | rpoB | β-lactam^s^ gene | Rv3826 | NA | DR gene | GRN | β-lactam^s^ gene ->DR gene | 2732.31 |
| Rv0667 | rpoB | β-lactam^s^ gene | Rv3159c | NA | DR gene | GRN | β-lactam^s^ gene ->DR gene | 2732.435 |
| Rv0667 | rpoB | β-lactam^s^ gene | Rv2726c | dapF | DR gene | GRN | β-lactam^s^ gene ->DR gene | 2732.562 |
| Rv0667 | rpoB | β-lactam^s^ gene | Rv3823c | mmpL8 | DR gene | GRN | β-lactam^s^ gene ->DR gene | 2732.838 |
| Rv0667 | rpoB | β-lactam^s^ gene | Rv1308 | atpA | DR gene | GRN | β-lactam^s^ gene ->DR gene | 2732.901 |
| Rv0667 | rpoB | β-lactam^s^ gene | Rv1338 | murI | DR gene | GRN | β-lactam^s^ gene ->DR gene | 2732.919 |
| Rv0667 | rpoB | β-lactam^s^ gene | Rv2927c | Rv2927c | DR gene | GRN | β-lactam^s^ gene ->DR gene | 2732.977 |
| Rv0667 | rpoB | β-lactam^s^ gene | Rv2223c | Rv2223c | DR gene | GRN | β-lactam^s^ gene ->DR gene | 2733.011 |
| Rv0667 | rpoB | β-lactam^s^ gene | Rv3818 | NA | DR gene | GRN | β-lactam^s^ gene ->DR gene | 2733.142 |
| Rv0667 | rpoB | β-lactam^s^ gene | Rv1310 | atpD | DR gene | GRN | β-lactam^s^ gene ->DR gene | 2733.261 |
| Rv0667 | rpoB | β-lactam^s^ gene | Rv1522c | NA | DR gene | GRN | β-lactam^s^ gene ->DR gene | 2733.286 |
| Rv0667 | rpoB | β-lactam^s^ gene | Rv2864c | Rv2864c | DR gene | GRN | β-lactam^s^ gene ->DR gene | 2733.459 |
| Rv0667 | rpoB | β-lactam^s^ gene | Rv2276 | NA | DR gene | GRN | β-lactam^s^ gene ->DR gene | 2733.516 |
| Rv0667 | rpoB | β-lactam^s^ gene | Rv2093c | tatC | DR gene | GRN | β-lactam^s^ gene ->DR gene | 2733.557 |
| Rv0667 | rpoB | β-lactam^s^ gene | Rv2752c | NA | DR gene | GRN | β-lactam^s^ gene ->DR gene | 2733.557 |
| Rv0667 | rpoB | β-lactam^s^ gene | Rv2722 | NA | DR gene | GRN | β-lactam^s^ gene ->DR gene | 2733.597 |
| Rv0667 | rpoB | β-lactam^s^ gene | Rv2256c | Rv2256c | DR gene | GRN | β-lactam^s^ gene ->DR gene | 2733.599 |
| Rv0667 | rpoB | β-lactam^s^ gene | Rv3921c | Rv3921c | DR gene | GRN | β-lactam^s^ gene ->DR gene | 2733.779 |
| Rv0667 | rpoB | β-lactam^s^ gene | Rv1025 | Rv1025 | DR gene | GRN | β-lactam^s^ gene ->DR gene | 2733.846 |
| Rv0667 | rpoB | β-lactam^s^ gene | Rv1949c | NA | DR gene | GRN | β-lactam^s^ gene ->DR gene | 2733.846 |
| Rv0667 | rpoB | β-lactam^s^ gene | Rv2730 | NA | DR gene | GRN | β-lactam^s^ gene ->DR gene | 2734.012 |
| Rv0667 | rpoB | β-lactam^s^ gene | Rv2198c | NA | DR gene | GRN | β-lactam^s^ gene ->DR gene | 2734.114 |
| Rv0667 | rpoB | β-lactam^s^ gene | Rv1306 | atpF | DR gene | GRN | β-lactam^s^ gene ->DR gene | 2734.147 |
| Rv0667 | rpoB | β-lactam^s^ gene | Rv2104c | NA | DR gene | GRN | β-lactam^s^ gene ->DR gene | 2734.469 |
| Rv0667 | rpoB | β-lactam^s^ gene | Rv1305 | atpE | DR gene | GRN | β-lactam^s^ gene ->DR gene | 2734.594 |
| Rv0667 | rpoB | β-lactam^s^ gene | Rv2926c | Rv2926c | DR gene | GRN | β-lactam^s^ gene ->DR gene | 2734.616 |
| Rv0667 | rpoB | β-lactam^s^ gene | Rv3727 | NA | DR gene | GRN | β-lactam^s^ gene ->DR gene | 2734.65 |
| Rv0667 | rpoB | β-lactam^s^ gene | Rv2721c | NA | DR gene | GRN | β-lactam^s^ gene ->DR gene | 2734.786 |
| Rv0667 | rpoB | β-lactam^s^ gene | Rv2094c | tatA | DR gene | GRN | β-lactam^s^ gene ->DR gene | 2734.808 |
| Rv0667 | rpoB | β-lactam^s^ gene | Rv2773c | dapB | DR gene | GRN | β-lactam^s^ gene ->DR gene | 2734.808 |
| Rv0667 | rpoB | β-lactam^s^ gene | Rv2068c | NA | DR gene | GRN | β-lactam^s^ gene ->DR gene | 2735.105 |
| Rv0667 | rpoB | β-lactam^s^ gene | Rv2518c | NA | DR gene | GRN | β-lactam^s^ gene ->DR gene | 2735.162 |
| Rv0667 | rpoB | β-lactam^s^ gene | Rv2107 | NA | DR gene | GRN | β-lactam^s^ gene ->DR gene | 2735.218 |
| Rv0112 | gca | β-lactam^s^ gene | Rv0667 | rpoB | DR gene | GRN | β-lactam^s^ gene ->DR gene | 2735.338 |
| Rv0667 | rpoB | β-lactam^s^ gene | Rv1497 | NA | DR gene | GRN | β-lactam^s^ gene ->DR gene | 2735.409 |
| Rv0667 | rpoB | β-lactam^s^ gene | Rv0806c | NA | DR gene | GRN | β-lactam^s^ gene ->DR gene | 2735.453 |
| Rv0667 | rpoB | β-lactam^s^ gene | Rv1307 | atpH | DR gene | GRN | β-lactam^s^ gene ->DR gene | 2735.577 |
| Rv0667 | rpoB | β-lactam^s^ gene | Rv2163c | pbpB | DR gene | GRN | β-lactam^s^ gene ->DR gene | 2735.647 |
| Rv0667 | rpoB | β-lactam^s^ gene | Rv0867c | NA | DR gene | GRN | β-lactam^s^ gene ->DR gene | 2735.739 |
| Rv0667 | rpoB | β-lactam^s^ gene | Rv1309 | atpG | DR gene | GRN | β-lactam^s^ gene ->DR gene | 2735.751 |
| Rv0667 | rpoB | β-lactam^s^ gene | Rv3903c | NA | DR gene | GRN | β-lactam^s^ gene ->DR gene | 2735.916 |
| Rv0667 | rpoB | β-lactam^s^ gene | Rv3682 | ponA2 | DR gene | GRN | β-lactam^s^ gene ->DR gene | 2736.146 |
| Rv0667 | rpoB | β-lactam^s^ gene | Rv2150c | ftsZ | DR gene | GRN | β-lactam^s^ gene ->DR gene | 2736.322 |
| Rv0667 | rpoB | β-lactam^s^ gene | Rv3820c | NA | DR gene | GRN | β-lactam^s^ gene ->DR gene | 2736.327 |
| Rv0667 | rpoB | β-lactam^s^ gene | Rv3627c | Rv3627c | DR gene | GRN | β-lactam^s^ gene ->DR gene | 2736.343 |
| Rv0667 | rpoB | β-lactam^s^ gene | Rv1009 | NA | DR gene | GRN | β-lactam^s^ gene ->DR gene | 2736.436 |
| Rv0194 | Rv0194 | β-lactam^s^ gene | Rv0667 | rpoB | DR gene | GRN | β-lactam^s^ gene ->DR gene | 2736.462 |
| Rv0667 | rpoB | β-lactam^s^ gene | Rv1456c | Rv1456c | DR gene | GRN | β-lactam^s^ gene ->DR gene | 2736.478 |
| Rv0667 | rpoB | β-lactam^s^ gene | Rv2525c | NA | DR gene | GRN | β-lactam^s^ gene ->DR gene | 2736.486 |
| Rv0113 | gmhA | β-lactam^s^ gene | Rv0667 | rpoB | DR gene | GRN | β-lactam^s^ gene ->DR gene | 2736.564 |
| Rv0667 | rpoB | β-lactam^s^ gene | Rv2156c | murX | DR gene | GRN | β-lactam^s^ gene ->DR gene | 2736.822 |
| Rv0667 | rpoB | β-lactam^s^ gene | Rv2943 | NA | DR gene | GRN | β-lactam^s^ gene ->DR gene | 2736.871 |
| Rv0116c | ldtA | β-lactam^s^ gene | Rv0667 | rpoB | DR gene | GRN | β-lactam^s^ gene ->DR gene | 2736.872 |
| Rv0667 | rpoB | β-lactam^s^ gene | Rv1304 | atpB | DR gene | GRN | β-lactam^s^ gene ->DR gene | 2736.96 |
| Rv0667 | rpoB | β-lactam^s^ gene | Rv1846c | blaI | DR gene | GRN | β-lactam^s^ gene ->DR gene | 2736.99 |
| Rv0667 | rpoB | β-lactam^s^ gene | Rv2147c | Rv2147c | DR gene | GRN | β-lactam^s^ gene ->DR gene | 2737.077 |
| Rv0667 | rpoB | β-lactam^s^ gene | Rv1258c | NA | DR gene | GRN | β-lactam^s^ gene ->DR gene | 2737.099 |
| Rv0667 | rpoB | β-lactam^s^ gene | Rv1810 | NA | DR gene | GRN | β-lactam^s^ gene ->DR gene | 2737.225 |
| Rv0667 | rpoB | β-lactam^s^ gene | Rv1521 | NA | DR gene | GRN | β-lactam^s^ gene ->DR gene | 2737.262 |
| Rv0667 | rpoB | β-lactam^s^ gene | Rv2151c | ftsQ | DR gene | GRN | β-lactam^s^ gene ->DR gene | 2737.349 |
| Rv0030 | Rv0030 | β-lactam^s^ gene | Rv0667 | rpoB | DR gene | GRN | β-lactam^s^ gene ->DR gene | 2737.501 |
| Rv0667 | rpoB | β-lactam^s^ gene | Rv3065 | NA | DR gene | GRN | β-lactam^s^ gene ->DR gene | 2737.622 |
| Rv0667 | rpoB | β-lactam^s^ gene | Rv2155c | murD | DR gene | GRN | β-lactam^s^ gene ->DR gene | 2737.719 |
| Rv0667 | rpoB | β-lactam^s^ gene | Rv1503c | Rv1503c | DR gene | GRN | β-lactam^s^ gene ->DR gene | 2737.759 |
| Rv0667 | rpoB | β-lactam^s^ gene | Rv2373c | dnaJ2 | DR gene | GRN | β-lactam^s^ gene ->DR gene | 2737.946 |
| Rv0667 | rpoB | β-lactam^s^ gene | Rv2181 | NA | DR gene | GRN | β-lactam^s^ gene ->DR gene | 2738.016 |
| Rv0667 | rpoB | β-lactam^s^ gene | Rv1884c | NA | DR gene | GRN | β-lactam^s^ gene ->DR gene | 2738.098 |
| Rv0011c | Rv0011c | β-lactam^s^ gene | Rv0667 | rpoB | DR gene | GRN | β-lactam^s^ gene ->DR gene | 2738.233 |
| Rv0406c | Rv0406c | β-lactam^s^ gene | Rv0667 | rpoB | DR gene | GRN | β-lactam^s^ gene ->DR gene | 2738.489 |
| Rv0667 | rpoB | β-lactam^s^ gene | Rv1730c | NA | DR gene | GRN | β-lactam^s^ gene ->DR gene | 2738.512 |
| Rv0667 | rpoB | β-lactam^s^ gene | Rv1433 | NA | DR gene | GRN | β-lactam^s^ gene ->DR gene | 2738.519 |
| Rv0667 | rpoB | β-lactam^s^ gene | Rv0849 | NA | DR gene | GRN | β-lactam^s^ gene ->DR gene | 2738.718 |
| Rv0050 | ponA1 | β-lactam^s^ gene | Rv0667 | rpoB | DR gene | GRN | β-lactam^s^ gene ->DR gene | 2738.735 |
| Rv0014c | pknB | β-lactam^s^ gene | Rv0667 | rpoB | DR gene | GRN | β-lactam^s^ gene ->DR gene | 2739.126 |
| Rv0015c | pknA | β-lactam^s^ gene | Rv0667 | rpoB | DR gene | GRN | β-lactam^s^ gene ->DR gene | 2739.146 |
| Rv0667 | rpoB | β-lactam^s^ gene | Rv1847 | Rv1847 | DR gene | GRN | β-lactam^s^ gene ->DR gene | 2739.35 |
| Rv0667 | rpoB | β-lactam^s^ gene | Rv2154c | ftsW | DR gene | GRN | β-lactam^s^ gene ->DR gene | 2739.502 |
| Rv0667 | rpoB | β-lactam^s^ gene | Rv3107c | NA | DR gene | GRN | β-lactam^s^ gene ->DR gene | 2739.646 |
| Rv0667 | rpoB | β-lactam^s^ gene | Rv3677c | NA | DR gene | GRN | β-lactam^s^ gene ->DR gene | 2739.799 |
| Rv0667 | rpoB | β-lactam^s^ gene | Rv3330 | dacB1 | DR gene | GRN | β-lactam^s^ gene ->DR gene | 2739.826 |
| Rv0667 | rpoB | β-lactam^s^ gene | Rv2152c | murC | DR gene | GRN | β-lactam^s^ gene ->DR gene | 2739.996 |
| Rv0667 | rpoB | β-lactam^s^ gene | Rv2224c | NA | DR gene | GRN | β-lactam^s^ gene ->DR gene | 2740.054 |
| Rv0667 | rpoB | β-lactam^s^ gene | Rv2158c | murE | DR gene | GRN | β-lactam^s^ gene ->DR gene | 2740.129 |
| Rv0667 | rpoB | β-lactam^s^ gene | Rv1218c | NA | DR gene | GRN | β-lactam^s^ gene ->DR gene | 2740.314 |
| Rv0667 | rpoB | β-lactam^s^ gene | Rv2157c | murF | DR gene | GRN | β-lactam^s^ gene ->DR gene | 2740.322 |
| Rv0667 | rpoB | β-lactam^s^ gene | Rv2844 | NA | DR gene | GRN | β-lactam^s^ gene ->DR gene | 2740.322 |
| Rv0667 | rpoB | β-lactam^s^ gene | Rv3635 | NA | DR gene | GRN | β-lactam^s^ gene ->DR gene | 2740.514 |
| Rv0667 | rpoB | β-lactam^s^ gene | Rv2258c | Rv2258c | DR gene | GRN | β-lactam^s^ gene ->DR gene | 2740.812 |
| Rv0667 | rpoB | β-lactam^s^ gene | Rv2450c | NA | DR gene | GRN | β-lactam^s^ gene ->DR gene | 2741.301 |
| Rv0667 | rpoB | β-lactam^s^ gene | Rv2509 | NA | DR gene | GRN | β-lactam^s^ gene ->DR gene | 2741.488 |
